# Coupling uranyl upcycling with photoelectrochemical urea degradation for radioactive organic wastewater management

**DOI:** 10.1038/s41467-026-72145-w

**Published:** 2026-04-17

**Authors:** Huihui Jin, Zewen Shen, Chumin Yan, Yang Liu, Yingying Tian, Hongliang Bao, Zhuoyu Ji, Yezi Hu, Guixia Zhao, Xiangke Wang, Xiubing Huang

**Affiliations:** 1 College of Environmental Science and Engineering, North China Electric Power University, Beijing, P. R. China; 2 Beijing Advanced Innovation Center for Materials Genome Engineering, Beijing Key Laboratory of Function Materials for Molecule & Structure Construction, School of Materials Science and Engineering, University of Science and Technology Beijing, Beijing, P. R. China; 3 Key Laboratory of Interfacial Physics and Technology, Shanghai Institute of Applied Physics, Chinese Academy of Sciences, Shanghai, P. R. China

**Keywords:** Pollution remediation, Electrocatalysis

## Abstract

Radioactive organic wastewater management concerning metal source recovery and organic matter degradation conforms well to the notion of circular economy and environmental protection, but the concurrent conversion in an integrated process is challenging and has been rarely studied. Here, we introduce a high-performance photoelectrochemical system for uranyl extraction and the simultaneous degradation of co-existing urea to manage the treatment of uranyl-containing radioactive organic wastewater generated from the production of nuclear fuel for fourth-generation high-temperature gas-cooled reactor. Cost-efficient self-standing Ni-modified TiO_2_ nanotube arrays (Ni/TiO_2_) and β-cyclodextrin polymer (P-CDP@CC) were used as photoanode and cathode, respectively. Concurrent photoanodic urea degradation and cathodic uranyl reduction towards UO_2_ from low-level radioactive organic wastewater demonstrated a uranyl extraction efficiency of 99.08% and the urea degradation efficiency of nearly 100% under a current density of 4.5 mA/cm^2^ within 8 hours in a single-compartment electrolysis cell. This work provides an effective, environmentally benign approach to upcycle uranium resources and purify organic pollutants from complicated wastewater matrix.

## Abstract (web summary)

Radioactive organic wastewater poses environmental challenges. Here, authors develop a photoelectrochemical system, Ni-modified TiO_2_ nanotube arrays photoanode and β-cyclodextrin polymer cathode, that simultaneously achieves over 99% uranyl extraction and nearly complete urea removal.

## Introduction

Nuclear power is a reliable low-carbon energy source, preventing approximately 66 Gt of global CO_2_ emissions over the past 50 years^1,2^. Until 2022, it accounted for approximately 4.72% electricity generation in China and for 11% in worldwide^3,4^. While nuclear industry development brings convenience, it also annually discharged the large quantities of wastewater during mining^5^, nuclear fuel manufacturing^6^, and spent fuel reprocessing processes^7^, which are characterized by radioactivity and chemical toxicity, posing a dual threat to human health and the ecological environment^8–11^. As a typical wastewater generated from the production of nuclear fuel for fourth-generation high-temperature gas-cooled reactor, uranyl-containing radioactive organic wastewater contains a quantity of uranyl (50 ~ 100 ppm), high ammonium nitrate (~800 ppm) and high urea (~12,000 ppm)^12,13^. Although plenty of research focused on exploring how to efficiently remove uranyl or the coexisting organic matter from wastewater^14–16^, developing the uranyl extraction coupled with organic degradation in an integrated process is still lacking so far^17^. The competition, complexation, and antagonistic effects between uranyl and co-existing organic matter, which affect uranyl adsorption, migration and uranyl redox potentials^18^, have posed great challenges to conventional treatment technologies such as adsorption, ion exchange, and chemical precipitation.

Electrochemical method and photoelectrochemical (PEC) method have significant potential for the pollutants treatment by virtue of the cathodic reductions and anodic oxidation reactions^19–22^. For example, O’Connor et al. constructed the electrochemical system that couples a cathode for Cu and Eu efficient recovery with an anode for oxygen evolution reaction (OER)^23^. With the combination of photo-excitation, which offsets some of the electrical energy required for electrochemical, decreasing the requirement of bias voltage^24,25^. Zeng et al. introduce an efficient photoelectrochemical platform for concurrent Cu recovery and water oxidation from a real electroplating wastewater^26^. He et al. applied an FTO glass cathode and a TiO_2_ photoanode for uranyl reduction to UO_2_ and oxygen evolution reaction, respectively^27^, requiring large additional bias and higher energy consumption. Compared with OER, the simultaneous urea oxidation reaction (UOR) is considered as an ideal alternative half reaction due to the lower theoretical potential of 0.37 V^28,29^, which is the rightly perfect desired coupling reaction for the management of the targeted radioactive organic wastewater.

To extremely diminish the cost for electrode preparation, here, we have proposed a photoelectrolysis strategy to construct high-efficiency photoanode and cathode, i.e., the respective self-standing Ni/TiO_2_ and β-cyclodextrin polymer (P-CDP@CC), for concurrent urea degradation and uranyl extraction from complicated wastewater matrix in a single-compartment electrolysis cell. In this system, the Ni/TiO_2_ photoanode promotes interfacial charge transfer, boosting the formation of surface-bound hydroxyl radicals (·OH), thus accelerating urea degradation kinetics with reduced energy consumption, while the hydroxyl groups of the P-CDP@CC cathode enable efficient binding to uranyl, and thus resulted in in situ uranyl extraction as UO_2_. By utilization of UOR rather than the OER at the photoanode, uranyl extraction efficiency of 99.08% and a simultaneous urea degradation efficiency of nearly 100% under a current density of 4.5 mA/cm^2^ was realized with decreased overall voltage in two-electrode system. This study potentially provides an efficient way for the concurrent uranyl resource recovery and organic pollutant treatment from a complicated low-level uranyl-containing radioactive organic wastewater matrix.

## Results

### Materials synthesis and characterization

**Fig. 1:**
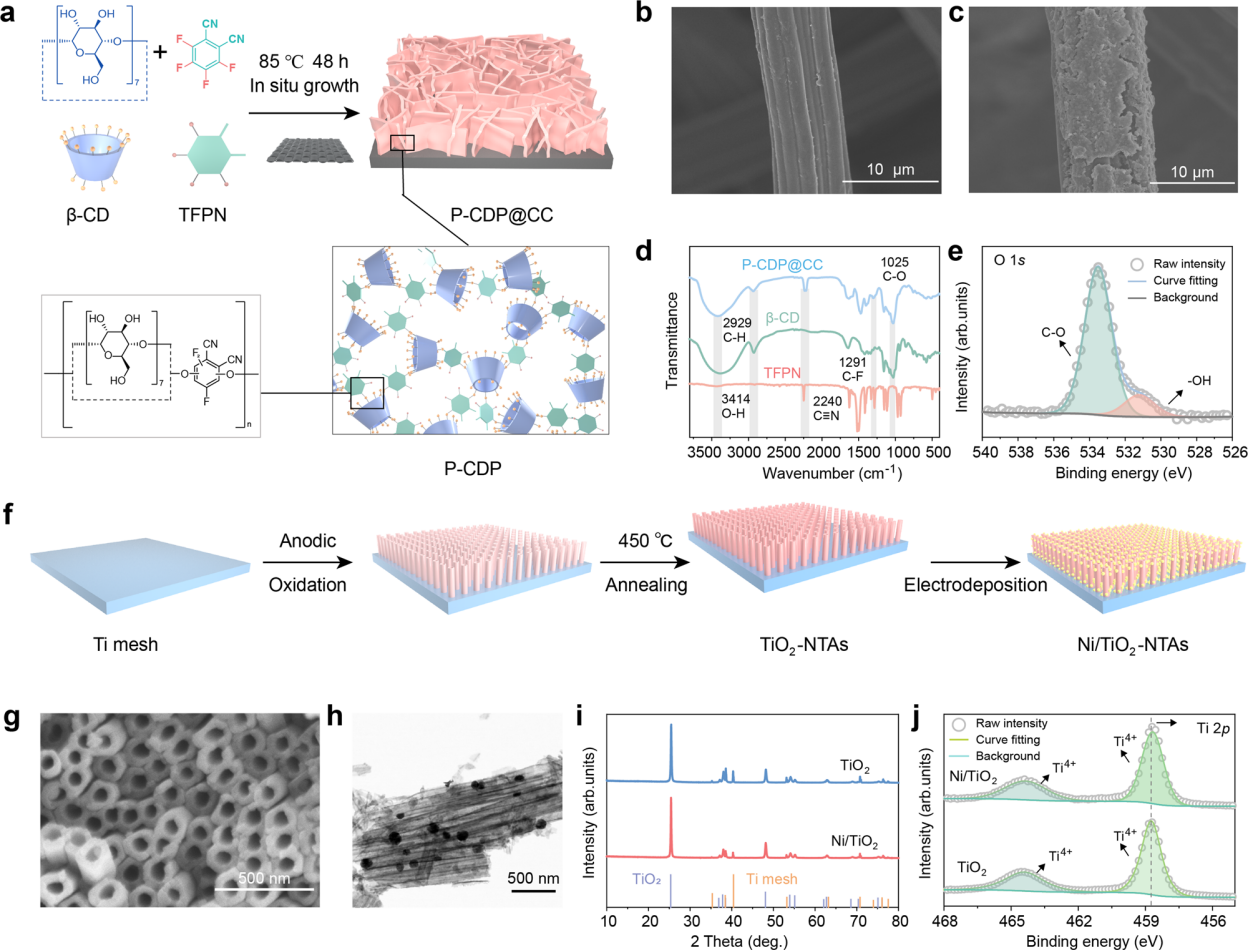
Fabrication and characterization of the P-CDP@CC, TiO_2_ and Ni/TiO_2_. **a** Schematic diagram of the fabricating process for the P-CDP@CC. **b**, **c** SEM images of carbon cloth and P-CDP@CC. **d** FT-IR spectra of P-CDP@CC compared with starting materials, respectively. **e** O 1 *s* XPS spectra of P-CDP@CC. **f** The schematic illustration for TiO_2_ and Ni/TiO_2_. **g**, **h** SEM and TEM images of Ni/TiO_2_. **i** XRD patterns of TiO_2_ and Ni/TiO_2_. **j** XPS spectra of Ti 2*p* for TiO_2_ and Ni/TiO_2_.

As shown in Fig. 1a, we develop the in situ growth of a polyarylether-based polymer (P-CDP) on the carbon cloth to fabricate a self-standing P-CDP@CC cathode material. Scanning electron microscopy (SEM) images of the carbon cloth and P-CDP@CC show that irregular folded lamellar structures were uniformly grown on the smooth surface of the carbon cloth to form the yellow material P-CDP@CC (Fig. 1b, c and Supplementary Fig. 1). As shown by Fourier transfer infrared (FT-IR) spectra in Fig. 1d, the characteristic peaks at 3414, 2929 and 1025 cm^−^^1^ ascribed respectively to –OH, -CH and C-O stretching vibrations^30^, providing the presence of β-Cyclodextrin (β-CD) moieties in P-CDP@CC. The stretching vibrations of C ≡ N and C-F at 2240 and 1291 cm^−1^ belonging to 3,4,5,6-Tetrafluorophthalonitrile (TFPN) can also be observed^31^, where the weakening of the peak intensity of C-F indicated the partial substitution, demonstrating that P-CDP was successfully loaded onto the carbon cloth substrate^32,33^. In addition, X-ray photoelectron spectroscopy (XPS) confirmed the successful polymerization of P-CDP@CC (Fig. 1e and Supplementary Fig. 2), and the existence of abundant oxygen-containing functional groups on P-CDP@CC electrode synergistically facilitated uranyl fixation^30^. Determined by X-ray diffraction (XRD), P-CDP@CC electrode showed broad diffraction peaks at 15 ~ 20° reveals an amorphous structure, which is the typical characteristic of polymer (Supplementary Fig. 3). Thereafter, as shown in the split peak fitting spectra of O 1 *s* and C 1 *s*, there exist abundant C-OH and C = O groups on the surface of carbon cloth (Supplementary Fig. 4). Therefore, the possible interaction between polyarylether-based polymer and carbon cloth can be: (1) hydrogen bonds between C-OH groups on the surface of carbon cloth and -OH groups on polyarylether-based polymer; (2) hydrogen bonds between C-OH groups on the surface of carbon cloth and F atoms from TFPN.

As a photoanode for urea degradation, a Ni/TiO_2_ nanotube array was employed. The TiO_2_ nanotube was prepared by the anodizing method with a Ti mesh as the substrate. Subsequently, the Ni nanoparticles were loaded to the surface of TiO_2_ nanotube arrays by electrodeposition (Fig. 1f), then the electrodeposition conditions were optimized, and the optimized Ni/TiO_2_ photoanode was characterized (Supplementary Fig. 5 and Supplementary Table 1). SEM images showed that the Ti mesh substrate of Ni/TiO_2_ electrode was neatly covered with nanotubes with a uniform diameter of about 85 nm (Fig. 1g). For Ni/TiO_2_ electrode, Ni particles with an average diameter of ca. 50 nm are attached on the surface of the nanotube (Fig. 1h). Additionally, the energy-dispersive spectroscopy (EDS) mapping images of Ni/TiO_2_ show the well dispersed particles without aggregation (Supplementary Fig. 6). These results demonstrate that the Ni nanoparticles are deposited on the surface of TiO_2_ nanotube. XRD pattern indicated anatase TiO_2_ (PDF # 04-0477) without additional diffraction peaks resulting from Ni nanoparticles (Fig. 1i).

Moreover, the chemical composition and valence states of Ni/TiO_2_ are investigated by XPS. Only two Ti 2*p* peaks centered at 464.44 and 458.74 eV with a splitting of 5.7 eV are observed in TiO_2_, which are typical Ti (IV) 2*p*_1/2_ and Ti (IV) 2*p*_3/2_ peaks in anatase TiO_2_^34^. For Ni/TiO_2_, the Ti 2*p*_1/2_ and Ti 2*p*_3/2_ peaks of Ti^4+^ appear at 464.37 and 458.65 eV, respectively, which slightly shift to low binding energy (Fig. 1j). In the Ni 2*p* spectrum of Ni/TiO_2_, the Ni 2*p*_1/2_ and Ni 2*p*_3/2_ peaks of Ni^2+^ species observed at 856.14 and 873.82 eV, respectively. Additionally, Ni^0^ was identified by binding energies of 852.90 and 869.17 eV (Supplementary Fig. 7). In the meantime, the O 1 *s* spectrum of Ni/TiO_2_ was deconvoluted into two peaks at 529.85 and 531.16 eV, assigned as M-O species (M is Ti or Ni) and surface OH species^35^, respectively, which moved to lower binding energies compared to TiO_2_, indicating the variation of microelectronic environments for the O species upon the formation of Ni/TiO_2_^36^ (Supplementary Fig. 8). The results of XPS further demonstrate the successful loading of Ni nanoparticles to the surface of TiO_2_ nanotube.

### Electrochemical upcycling of uranyl with self-standing P-CDP@CC electrode

**Fig. 2:**
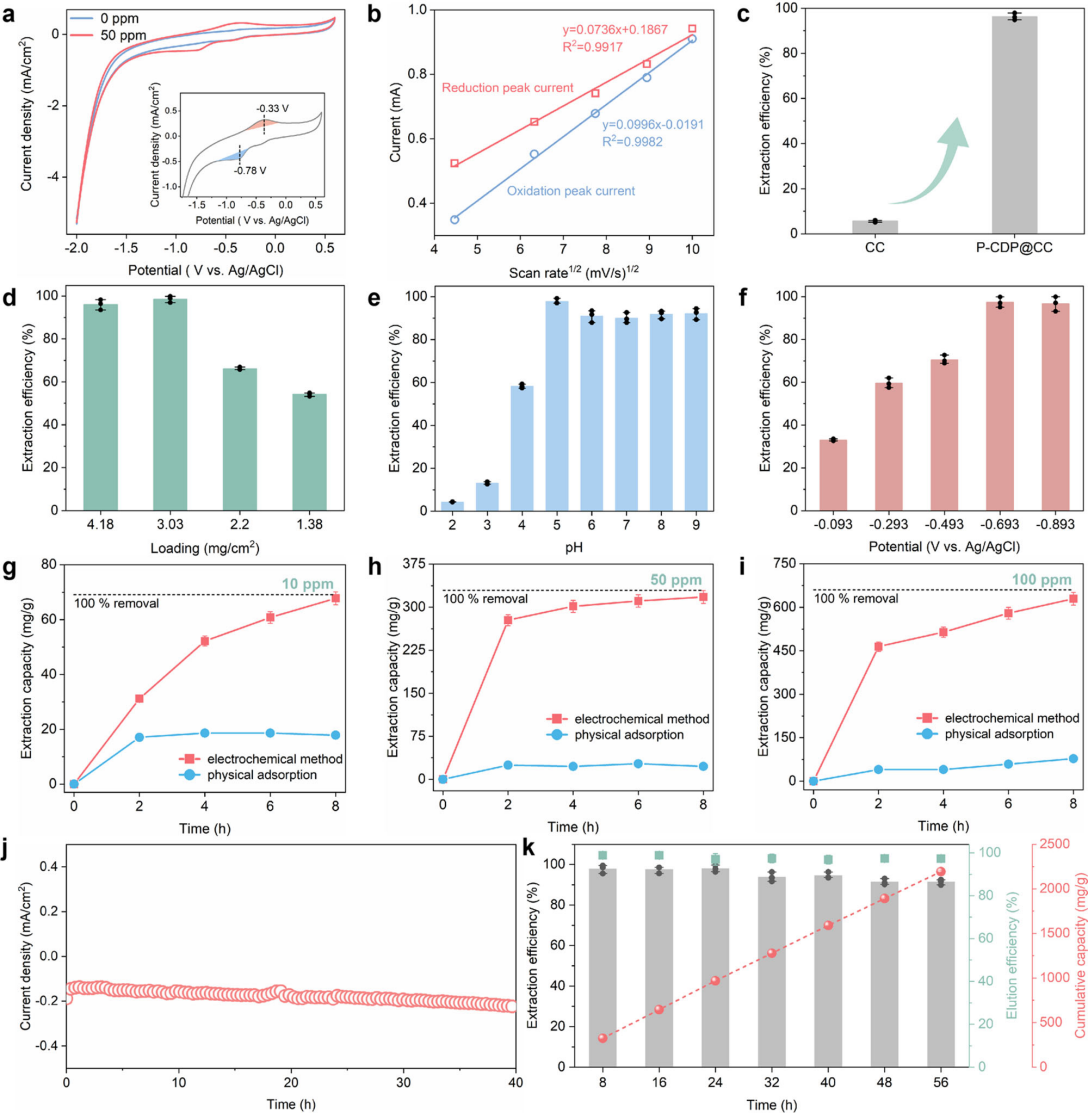
Electrochemical uranyl extraction of P-CDP@CC electrode. **a** CV curves of P-CDP@CC electrode with various uranyl concentrations at a scan rate of 50 mV/s. The illustration is an enlarged view of the selected region. **b** Relationship between reduction (oxidation) peak currents and the square root of scan rate. **c** The electrochemical uranyl extraction efficiency on P-CDP@CC and bare carbon cloth electrodes. Electrochemical extraction of uranyl on P-CDP@CC electrode **d** with different loading mass, **e** under different pH conditions, **f** at different potentials. Comparison of uranyl extraction with the electrochemical method and the physical adsorption with various uranyl concentrations of **g** ~ 10 ppm, **h** ~ 50 ppm, **i** ~ 100 ppm. **j** Chronoamperometry curve of P-CDP@CC electrode at −0.693 V vs. Ag/AgCl. **k** The recycling test of P-CDP@CC.(Potential = −0.693 V vs. Ag/AgCl, C_U_ = 50 ppm), (uranyl extraction efficiency: gray, elution efficiency: green, cumulative capacity: red). The error bars in **c-i** and **k** denote the standard deviation where *n* = 3.

Cyclic voltammetry (CV) curves of P-CDP@CC electrode in 0.1 M NaNO_3_ in the absence and presence of 50 ppm uranyl were measured (Fig. 2a). In 0.1 M NaNO_3_ electrolyte, no peaks appeared in the CV curve. While in the electrolyte with addition of uranyl, the CV curve of P-CDP@CC electrode exhibited significant oxidation and reduction peaks at −0.33 V and −0.78 V vs. Ag/AgCl, indicating the redox reactions of uranyl. Furthermore, Fig. 2b and Supplementary Fig. 9 showed the linear relations between the oxidation/reduction peak current and the square root of scanning rates, indicating that both the reductive conversion and the oxidative conversion were diffusion-controlled reaction^37^.

As shown in Fig. 2c, 96.0% of uranyl was extracted with P-CDP@CC electrode within 8 hours, which was significantly superior to that of bare carbon cloth. With the electrode mass loading increased from 1.38 to 3.03 mg/cm^2^, the uranyl extraction efficiency improved from 54.86 % to 98.91 % (Fig. 2d). However, the extraction was slightly impeded at a mass loading of 4.18 mg/cm^2^, which is supposed due to the sluggish electron/ion transportation^38^. The pH not only controls uranyl species in solution, but also affects the protonation of the hydroxyl groups at electrode surface, thus the influence of pH on the electrolytic uranyl extraction over P-CDP@CC electrode was further evaluated. As illustrated in Fig. 2e, when pH is as low as 3.0, only 13% of uranyl is extracted, attributing to the protonation of the hydroxyl groups on the P-CDP@CC electrode, leading to electrostatic repulsion between electrode surface and the uranyl cations. At pH 4.0, the extraction sharply improved to 58.13%, and the optimal extraction of 96.99% is obtained at pH 5.0. With pH increases to 6 and 9, the extraction remained consistently above 90%, which was supposed due to that the repulsion effect is largely weakened since electrode surface tends to be electrically neutral charged. Hence, pH value of the electrolyte was set to 5 for further studies. Increasing the applied voltage from −0.093 V to −0.893 V vs. Ag/AgCl, the electrochemical uranyl extraction over the P-CDP@CC electrode rose from 32.62 % to 96.99% (Fig. 2f). Moreover, as the initial concentration of uranyl varied from 10 to 100 ppm, the electrochemical uranyl extraction performance was substantially higher than physical adsorption, and the difference became greater with a higher initial uranyl concentration (Fig. 2g–i). An extraction efficiency of 90.99% and an extraction capacity of 627.45 mg/g were achieved with an initial concentration of 100 ppm. Subsequently, with 0.1 M Na_2_CO_3_ electrolyte and 0.3 V vs. Ag/AgCl reverse bias, 99.86% of immobilized U on the electrode can be oxidized into soluble U(VI), thereby achieving uranyl ultra-efficiently recovery (Supplementary Figs. 10, 11). The long-term utilization of such P-CDP@CC electrode for electrochemical uranyl extraction showed the slowly decayed electro-activity (Fig. 2j). Additionally, after 7 consecutive extraction-elution cycles, more than 90 % of extraction and elution efficiency for uranyl can still be achieved, leading to an accumulated extraction capacity of 2192.90 mg/g (Fig. 2k). The FT-IR and SEM characterizations of P-CDP@CC electrode after extraction-elution cycling test suggest the high stability of P-CDP@CC (Supplementary Fig. 12). Notably, in the presence of several co-existing ions, the proportion of U reached 99.15% among the metal elements in precipitate after electrochemical extraction, which was much higher than other interfering ions (Supplementary Fig. 13), indicating that the P-CDP@CC electrode exhibited high anti-interfering ability to co-existing ions.

**Fig. 3:**
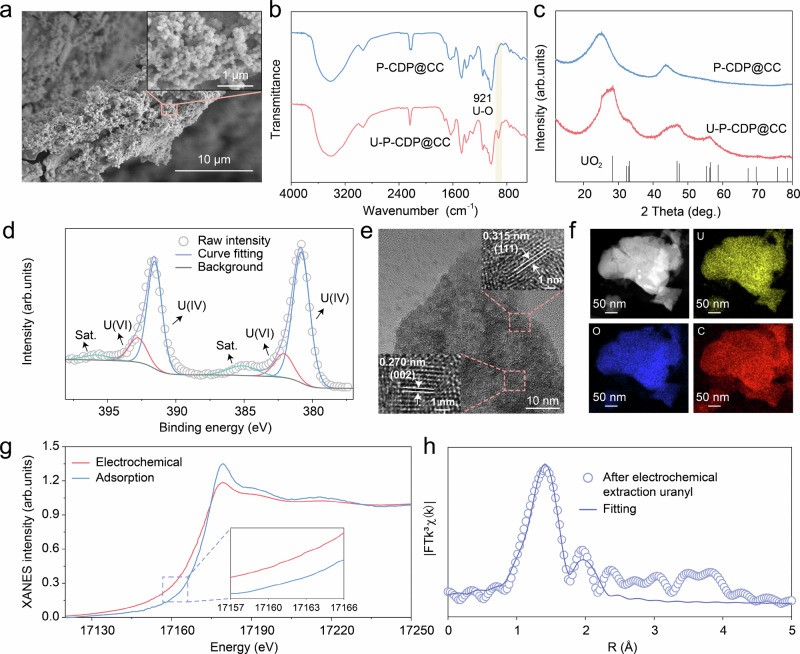
Interpretation of electrochemical extraction mechanism of uranyl. **a** SEM image of P-CDP@CC electrode after electrochemical uranyl extraction (C_U_ = 1000 ppm). Insets show the morphology of the electrodeposited particles at a higher magnification. **b** FT-IR spectrum and (**c**) XRD pattern comparison before and after electrochemical uranyl extraction. **d** U 4 *f* XPS spectrum and **e** HRTEM image of P-CDP@CC electrode after electrochemical uranyl extraction. Inset: magnified HRTEM image. **f** EDS measurement of P-CDP@CC electrode after electrochemical uranyl extraction. **g** Comparison of U L_3_-edge XANES spectra for P-CDP@CC after uranyl adsorption and uranyl electrochemical extraction. Inset: magnified pre-edge XANES region. **h** EXAFS R-space and corresponding fitting curves for P-CDP@CC after uranyl extraction.

To thoroughly clarify the extraction mechanism, the precipitate after reaction was characterized by SEM, XRD, XPS, FT-IR, and HRTEM. SEM image of the resulting electrode showed the presence of small nanoparticles sized around 150 nm (Fig. 3a). Furthermore, a new peak at 921 cm^−^^1^ belonging to the bond U = O arisen for P-CDP@CC electrode after electrochemical uranyl extraction (Fig. 3b). As indicated by XRD patterns, the extracted uranyl on the electrode surface was ascribed to UO_2_ (PDF#36-0089) (Fig. 3c). X-ray photoelectron spectrum (XPS) U 4 *f* distinctly designated coexisting U(VI) and U(IV) on the electrode, indicating the uranyl extraction underwent the electrochemical reduction process (Fig. 3d and Supplementary Fig. 14). Simultaneously, the HRTEM image in Fig. 3e revealed two lattice fringe spacings of 0.315 nm and 0.270 nm, which can be ascribed to the (111) and (002) planes in UO_2_ (PDF#36-0089). Accordingly, the energy-dispersive spectroscopy (EDS) results confirmed a homogeneous distribution of U for P-CDP@CC electrode (Fig. 3f).

The X-ray absorption near-edge structure (XANES) and the Fourier transform (FT) k^3^-weighted extended X-ray absorption fine structure (EXAFS) were further performed to probe the uranyl species evolution and coordination environment in P-CDP@CC. The U L_3_-edge XANES spectra showed that the adsorption edge position of electrochemical products had a slight shift towards the low-E side, indicating the uranyl extraction underwent the electrochemical reduction process (Fig. 3g). Furthermore, the FT curve had two main peaks at 1.4 and 1.9 Å, which could readily be assigned to the U-O_ax_ and U-O_eq_ scattering paths, respectively. Fitting the spectrum confirmed that in P-CDP@CC, a uranyl center is coordinated by two axial oxygen atoms and four oxygen atoms from hydroxyl groups (Fig. 3h, Supplementary Fig. 15 and Supplementary Table 2).

Based on the above, the mechanism of the P-CDP@CC electrode primarily includes the following steps (Supplementary Fig. 16): positively charged uranyl ions in the solution are electroadsorbed onto the hydroxyl groups of the cathodic P-CDP@CC, where adsorbed uranyl is electroreduced to UO_2_ and deposited onto the P-CDP@CC electrode surface.

### Photoelectrochemical degradation urea

**Fig. 4:**
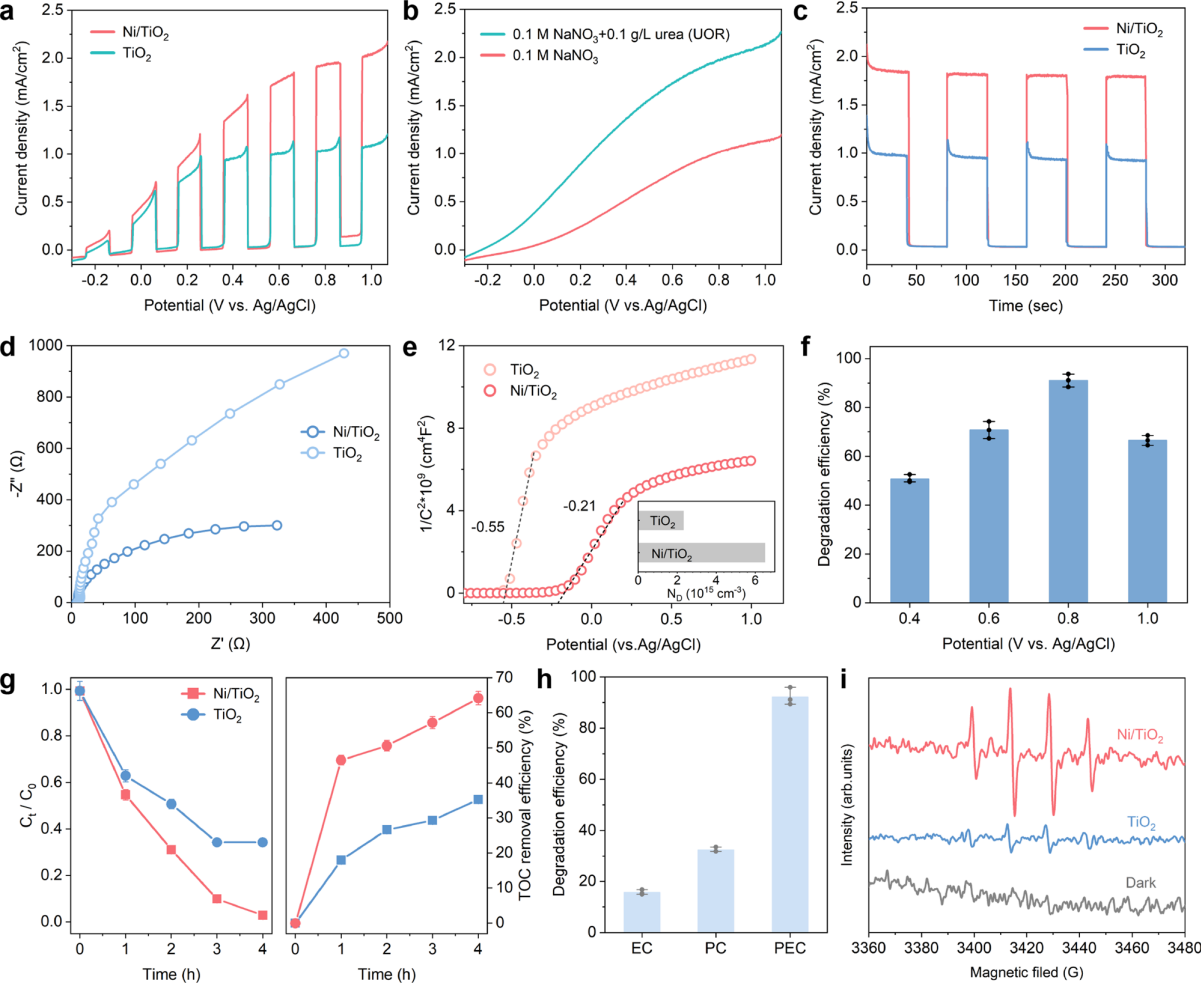
Photoelectrochemical urea degradation performance of TiO_2_ and Ni/TiO_2_ electrodes. **a** Photocurrent-potential curves of TiO_2_ and Ni/TiO_2_ photoanode under chopped illumination. **b** LSV curves of Ni/TiO_2_ photoanode in the absence and presence of 100 ppm urea under illumination. **c** Transient photocurrent responses of TiO_2_ and Ni/TiO_2_ photoanode at 0.8 V vs. Ag/AgCl. **d** Nyquist plots for TiO_2_ and Ni/TiO_2_ photoanode in 0.1 M NaNO_3_ under illumination. **e** Mott-Schottky plots of TiO_2_ and Ni/TiO_2_. Inset: Carrier densities of TiO_2_ and Ni/TiO_2_. **f** PEC urea degradation on Ni/TiO_2_ photoanode at different potentials under illumination (Potential = 0.8 V vs. Ag/AgCl, C_urea_ = 100 ppm). **g** Urea degradation and TOC removal efficiency on TiO_2_ and Ni/TiO_2_ (Potential=0.8 V vs. Ag/AgCl, C_urea_ = 100 ppm). **h** Comparison of urea degradation with the PC, EC and PEC. **i** EPR spectra of the TiO_2_ and Ni/TiO_2_ photoanode with DMPO used as s spin-trapping agent. The error bars in (**f**–**h**) denote the standard deviation where *n* = 3.

By using linear sweep voltammetry (LSV) under irradiation of a 300 W xenon lamp in 0.1 M NaNO_3_ solution containing 100 ppm urea, the J-V curves of TiO_2_ and Ni/TiO_2_ photoanode were measured. Figure 4a shows that the oxidation current density of the TiO_2_ and Ni/TiO_2_ photoanodes increased with increasing the applied anodic potential, among which the Ni/TiO_2_ exhibited higher photocurrent density, reaching 1.928 mA/cm^2^ at 0.8 V vs. Ag/AgCl. The results indicate that Ni nanoparticles promote substantial electrocatalytic activity for the urea degradation reaction. Note that, the obviously reduced photocurrent density of Ni/TiO_2_ in 0.1 M NaNO_3_ aqueous electrolyte suggested a more preferred urea degradation reaction than the water oxidation reaction (Fig. 4b). As displayed in Fig. 4c, the photocurrent intensity of Ni/TiO_2_ is much higher than that of TiO_2_. To gain a deeper insight into the modulation of the supported Ni nanoparticles on PEC degradation reaction, the electrochemical impedance spectroscopy (EIS) and Mott-Schottky curves were assessed utilizing a three-electrode arrangement. Compared to TiO_2_, the Ni/TiO_2_ photoanode show greatly enhanced carrier densities (N_D_)^39^(Fig. 4e). The EIS plots indicated the fastest charge transfer kinetics across the Ni/TiO_2_ photoanode and electrolyte interface (Fig. 4d). The charge transfer resistance of Ni/TiO_2_ under light conditions is noticeably smaller than that under dark condition^40^ (Supplementary Fig. 17). Furthermore, the lowest characteristic peak frequency (< 100 Hz) observed for Ni/TiO_2_ in the Bode plot disclosed the longest charge carrier lifetime (τ_e_) in Ni/TiO_2_^41^ (Supplementary Fig. 18 and Supplementary Table 3). In the meantime, the Ni/TiO_2_ photoanode also shows an enhance electron lifetime (τ_n_), which was obtained from the open-circuit voltage decay (OCVD) measurement^42^ (Supplementary Fig. 19). Consequently, the introduction of Ni nanoparticle effectively inhibits photogenerated charge recombination. Additionally, the double-layer capacitance (C_dl_), reflecting the electrochemically active surface area (ECSA) of the photoanodes, was measured by CV in 0.1 M NaNO_3_ (Supplementary Fig. 20a, b). As shown in Supplementary Fig. 20c, the slope of the Ni/TiO_2_ photoanode exhibited a C_dl_ of 5.32 mF/cm^2^ under illumination, higher than the C_dl_ of 3.43 mF/cm^2^ for the TiO_2_ photoanode. Notably, the Ni/TiO_2_ had a substantially smaller Tafel slope than TiO_2_, indicating a rapid electron transfer rate and superior reaction kinetics (Supplementary Fig. 21).

The PEC urea degradation was conducted in a signal-compartment cell with a graphite rod and Ag/AgCl electrode serving as the counter and reference electrode, respectively. Within the range of 0.4-0.8 V vs. Ag/AgCl, the urea degradation efficiency increases with rising potential, and the optimal degradation of 91.11% is obtained at 0.8 V vs. Ag/AgCl (Fig. 4f). At 1.0 V vs. Ag/AgCl, OER competed and thus slightly decreased the degradation^43^. Furthermore, the Ni/TiO_2_ photoanode showed a high degradation efficiency of 97.07%, while the TiO_2_ photoanode showed only 65.52% during 4 hours continuous illumination at 0.8 V vs. Ag/AgCl (Fig. 4g). As shown in Fig. 4g and Supplementary Fig. 22, Ni/TiO_2_ displays a total organic carbon (TOC) and total nitrogen (TN) removal efficiency of 64.21% and 64.77% in PEC urea degradation at 0.8 V vs. Ag/AgCl over 4 h, which is higher than that obtained for TiO_2_ of 35.29% and 55.28%. These results demonstrate a facilitated effect from the supported Ni nanoparticles for urea degradation at TiO_2_. Simultaneously, a series of intermediates were identified through ion chromatography (IC) and the colorimetric method. Supplementary Fig. 23 shows the intermediates comprised nitrate ions (NO_2_^−^), nitrate (NO_3_^−^), ammonia (NH_4_^+^), carbonate (CO_3_^2^^−^), and cyanate (OCN^−^). Specifically, the concentration of NO_3_^-^-N displays a monotonically increasing tendency along with the reaction time, and the maximum concentration of 8.76 mg/L is obtained at 4 hours. Notably, the concentrations of NO_2_^−^-N and NH_4_^+^-N undergo an initial increase and lateral decline, indicating that NO_2_^−^ and NH_4_^+^ as intermediates were accumulated progressively at the initial stage and would further transform into products such as NO_3_^−^, N_2_, and NH_3_. In addition, the concentration of OCN^−^ and CO_3_^2^^−^ in the PEC system showed upswing trends as the reaction proceeded. Moreover, compared to photocatalysis (PC) and electrocatalysis (EC), PEC urea degradation showed an obvious advantage, confirming a strong synergistic effect (Fig. 4h).

To disclose the underlying mechanism, we further carried out radical quenching experiments. As shown in Supplementary Table 4, when Na_2_SO_3_, was used as a hole scavenger, the degradation almost ceased, confirming the important role of the photogenerated holes in PEC urea degradation. In the meantime, when tert-butanol (TBA) was used as a scavenger for ·OH, the degradation efficiency of urea was markedly reduced. Furthermore, electron paramagnetic resonance (EPR) spectra were obtained under light illumination with 5,5-dimethyl-1-poyrroline-N-oxide (DMPO) as a trapping agent. As shown in Fig. 4i, for Ni/TiO_2,_ the typical quarter signal for the DMPO-·OH adduct displayed, which was significantly stronger than that recorded in TiO_2_ system. These findings imply that the urea degradation is jointly participated by photogenerated holes and ·OH radicals.

According to the above analysis, we proposed the probable mechanism for PEC urea degradation on the Ni/TiO_2_ photoanode (Supplementary Fig. 24), which includes both indirect degradation by ·OH radicals and direct degradation by photogenerated holes. For the indirect pathway, ·OH would be generated through water oxidation by the photogenerated holes transferred to the interface of the Ni/TiO_2_ photoanode. ·OH converts one of the amino groups in urea to a nitroso group and then to a nitro group, leading to the formation of nitroformamide (NH_2_CONO_2_), which then undergoes hydrolysis to releases NO_2_^−^ and COONH_2_^−^^44,45^. NO_2_^−^ can be further rapidly transformed into NO_3_^−^ by ·OH. The resulting COONH_2_^−^ can undergo two different oxidation pathways. One is that COONH_2_^−^ is decomposed into NH_4_^+^, which was further oxidized to N_2_^46^; the other is that it is decomposed into CO_3_^2^^−^ and NO_3_^−^ by ·OH. For the direct pathway, urea decomposes into ammonia (NH_3_) and cyanate ions (OCN^−^) with the aid of photogenerated holes^47^. Subsequently NH_3_ is further oxidized to NO_2_^−^ or N_2_^48^, while OCN^-^ is oxidized ultimately to produce NH_3_, NO_3_^−^ and CO_3_^2^^−^.

### PEC urea degradation with simultaneous uranyl extraction in low-level radioactive organic wastewater

**Fig. 5:**
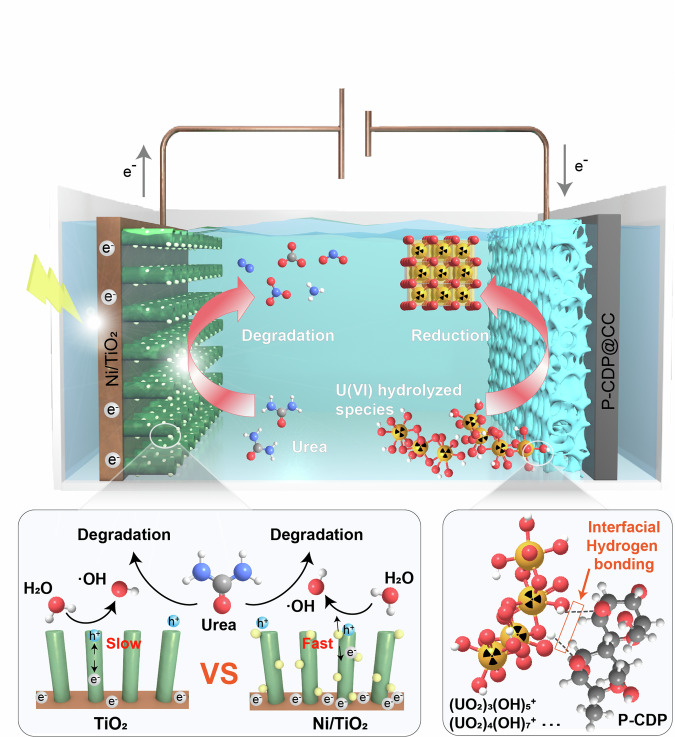
Possible reactive pathways of the bipolar coupling system. Ni/TiO_2_ photoanode is combined with P-CDP@CC cathode to achieve simultaneous urea degradation and uranyl extraction.

**Fig. 6:**
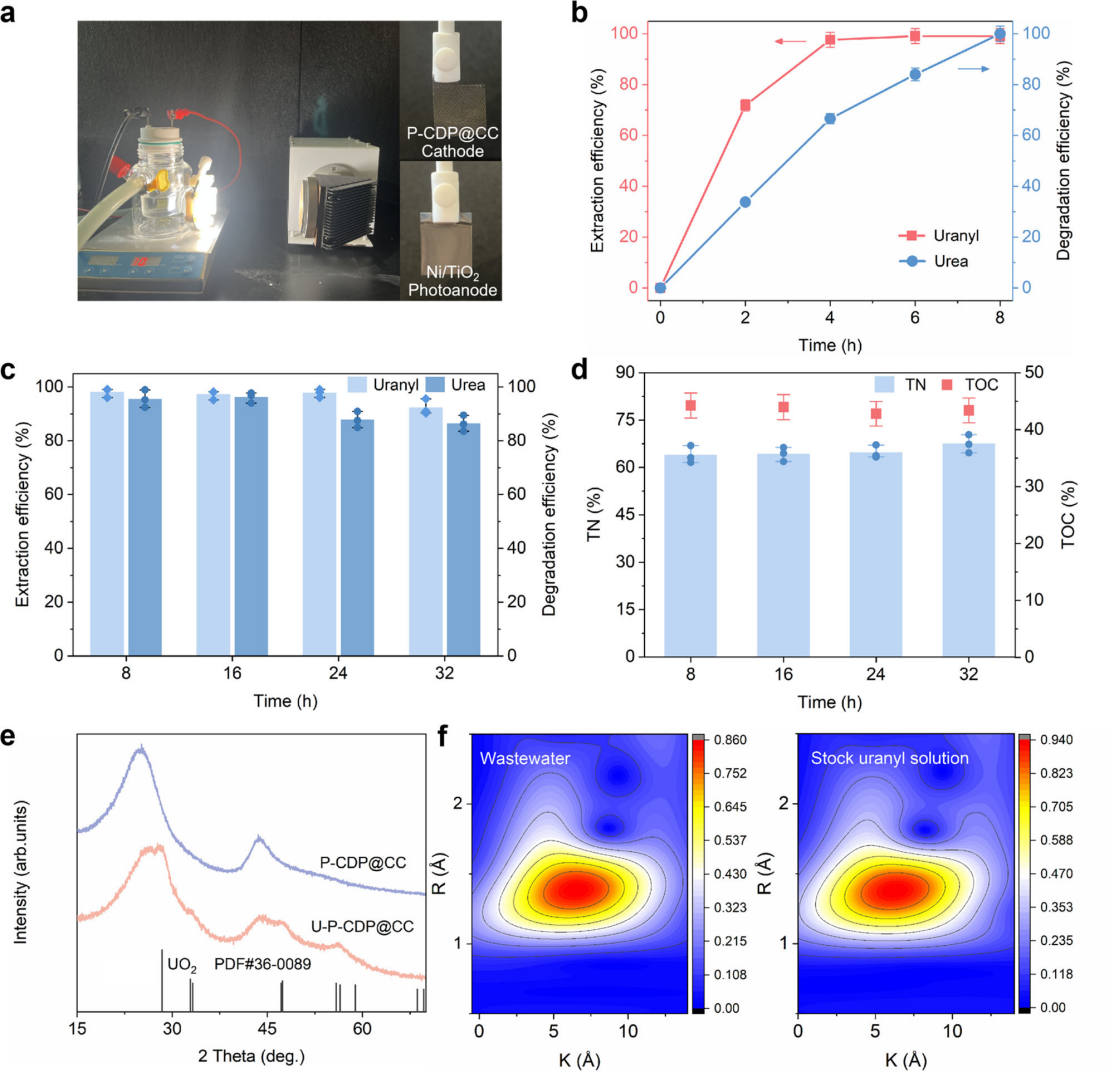
Simultaneous urea degradation and uranyl extraction from low-level radioactive organic wastewater. **a** Photograph of the bipolar PEC coupling system. Inset: photographs of P-CDP@CC cathode and Ni/TiO_2_ photoanode. **b** Uranyl extraction and urea degradation curves in low-level radioactive organic wastewater. **c** The reusability of the bipolar PEC system. **d** TOC and TN removal efficiency in the recycling test. **e** XRD patterns of P-CDP@CC after uranyl extraction. **f** WT contour plots for P-CDP@CC loaded with uranyl from stock uranyl solution and low-level radioactive organic wastewater. The error bars in (**b**–**d**) denote the standard deviation where *n* = 3.

We further combined the Ni/TiO_2_ photoanode with the P-CDP@CC cathode in a single-cell reaction for simultaneous urea degradation and uranyl extraction from low-level uranyl-containing radioactive organic wastewater (Supplementary Table 5 and Supplementary Fig. 25). Figures 5,6a illustrates the operational scheme for the bipolar coupling system. In this set-up, the Ni/TiO_2_ photoanode firstly absorbed solar light and produced electron and hole pairs. The introduction of Ni nanoparticles facilitated the separation of photogenerated holes and electrons, thus promoting efficient h^+^ transfer to interface of the photoanode, further boosting H_2_O oxidation to ·OH. Urea can be activated by hole or ·OH, which was degrade by reactive radicals. Simultaneously, photogenerated electrons and the electric current generation through the power source reach the P-CDP@CC cathode. U(VI) hydrolyzed species were electrochemically absorbed on the hydroxyl groups of the P-CDP@CC. The adsorbed uranyl was reduced to UO_2_ and deposited onto the P-CDP@CC electrode surface.

Figure 6b and Supplementary Figs. 26–28 demonstrated exceptional uranyl extraction efficiency of 99.08% and urea degradation efficiency of nearly 100% at a current density of 4.5 mA/cm^2^ and cell voltage of 3 V, which is far higher than that of those standalone technologies, i.e., adsorption and photocatalytic processes. Moreover, regardless of the initial high concentration of NH_4_^+^ and NO_3_^−^, the uranyl extraction efficiency and urea degradation remained consistently above 90% (Supplementary Fig. 29), indicating that the NH_4_^+^ and NO_3_^-^ concentrations did not exhibit an obvious influence on the uranyl extraction and urea degradation in coupling system.

After that, the interaction between the two processes of uranyl extraction and urea degradation was further systematically elucidated. It was reported that uranyl would complex with urea to form complexes such as UO_2_(NO_3_)·2(NH_2_)_2_CO, UO_2_(NO_3_)·4(NH_2_)_2_CO·H_2_O and UO_2_(NO_3_)·5(NH_2_)_2_CO·H_2_O under highly acidic conditions^49^. Considering the fact that the pH of the concerned radioactive organic wastewater fluctuates after near 9.0, thus the existence of such complexes from uranyl and urea in coupling system were excluded. Furthermore, with the urea concentration varied from 100 ppm to 12000 ppm, slight decrease in the uranyl extraction efficiency was observed in the initial 2 h (Supplementary Fig. 30a), which was ascribed to the competitive urea and uranyl adsorption on P-CDP@CC electrode surface. However, when the reaction time was prolonged to 4 h and then 8 h, regardless of the initial concentration of urea, the uranyl extraction efficiency by P-CDP@CC electrode remained consistently above 90%, indicating that the excessive urea could only interfere the initial uranyl reduction kinetics and with the persistent electric field, the uranyl could be reduced and grown into UO_2_ nanoparticle even when urea concentration is high as 12,000 ppm (Supplementary Fig. 30b). Thereafter, the urea degradation performances in the case with and without 50 ppm uranyl were compared. Obviously from the comparison, in the absence of uranyl, a degradation efficiency of 97.07% was achieved after 4 h, whereas an obvious suppression was demonstrated in the presence of uranyl (Supplementary Fig. 31). Such phenomenon may be attributed to the fact that the urea binding sites on TiO_2_ would be occupied by uranyl, which inhibits the efficient urea degradation. It’s noted that urea degradation is faster in kinetics than uranyl extraction, therefore, despite the inhibited urea degradation in the presence of uranyl, the coupled anodic oxidation and cathodic reduction are matched quite well in this couple system.

Continuously cycled operation for 32 h, the uranyl extraction and urea degradation efficiency still reached upon ~90% (Fig. 6c). As shown in Supplementary Fig. 32, 0.1 M Na_2_CO_3_ was used as the elution solution with 0.8 V reverse bias, the elution efficiency for uranyl can be kept as high as ca. 90% after 32 hours. Accordingly, the removal efficiency of TOC and TN remained consistently at around 40% and 65% over 32 h (Fig. 6d). The aforementioned results further substantiate that the bipolar coupling system for radioactive organic wastewater management shown remarkable cycle stability, enabling it to function consistently over extended periods (Supplementary Figs. 33–35). XRD pattern of resulted P-CDP@CC cathode confirmed the formation of UO_2_ (Fig. 6e). The satisfied growth of UO_2_ in the complex organic matrix suggested that the chelating of uranyl is not interfered by the waste matrix as revealed by EXAFS wavelet-transform (WT) analysis (Fig. 6f). Inductively coupled plasma optical emission spectrometry (ICP-OES) results showed that U proportion in the precipitate after electrochemical extraction is high as 99.40%, while other co-existing ions proportion below 0.30%, which agreed well with aforementioned analysis (Supplementary Fig. 36). In addition, the corresponding distribution coefficient (K_d_) was calculated up to 7.01 × 10^5^ mL/g (a K_d_ value exceeding 1.0×  10^5^ mL/g is usually considered as excellent material), demonstrating extremely outstanding advantage of the proposed coupled system for uranyl extraction. Subsequently, we also evaluated the enrichment factor of the electrochemical system^50^, which was 2249 for U.

Economic evaluation considering materials costs and energy consumption showed that with such a bipolar PEC coupling system, the overall cost was around 82 USD for the extraction of 1 kg U and the degradation of 1 kg urea (Supplementary Table 6), much lower than that with other electrochemical methods and physical adsorption methods reported in the literature (Supplementary Table 7).

## Discussion

In this study, we demonstrated uranyl extraction with simultaneous urea degradation using a Ni/TiO_2_ photoanode and a P-CDP@CC cathode for radioactive organic wastewater management. Via substitution of OER with UOR, the high-performance PEC cell enabled a superior uranyl extraction efficiency and urea removal efficiency with required voltage. On the one hand, the introduction of Ni nanoparticles in the Ni/TiO_2_ photoanode enhanced the efficiency of electron-hole separation, boosting the formation of surface-bound ·OH radicals, thus accelerating urea degradation kinetics with reduced energy consumption. On the other hand, the hydroxyl groups of the P-CDP@CC cathode enable efficient binding to uranyl, featuring satisfactory resistance to interference from co-existing urea and thus resulted in in situ uranyl extraction as UO_2_. Using this approach, a uranyl extraction efficiency of 99.08% and a urea degradation efficiency of nearly 100% from low-level uranyl-containing radioactive organic wastewater after 8 h were achieved. From pollution control and resource recovery perspectives, coupling uranyl upcycling and the co-existing urea degradation from complicated wastewater matrix has multiple sustainability gains. This work also provides a valuable reference paradigm for the highly efficient metal resource recovery and ultra-efficient organic pollutants management from complicated wastewater systems.

## Methods

### Materials

All reagents were purchased from commercial suppliers and used without further purification unless stated otherwise. β-Cyclodextrin (98%), 3,4,5,6-Tetrafluorophthalonitrile (97%), Potassium carbonate (K_2_CO_3_, 99%) and N,N-Dimethylformamide (DMF, 99.5%) were purchased from Innochem. Tetrahydrofuran (THF, 99.9%), Ammonium fluoride (NH_4_F, 99.99%), Ethylene glycol (99.9%), Nickel (II) chloride hexahydrate (NiCl_2_·6H_2_O, 98%) and Boric acid (H_3_BO_3_, 99.5%) were all provided by Aladdin. Carbon cloth and Ti mesh were purchased from Beijing Tianmei Hexing Technology Co., Ltd. Ultra-pure water was prepared from the Millipore system (18.25 MΩ·cm).

### Characterization

SEM images were displayed on a Hitachi SU 8100 Scanning Electron Microscope (Japan, 5 kV, 10 μA). SEM sample preparation: the electrode was cut and fixed on the sample stage with conductive tape. TEM, HRTEM and EDS images were recorded on a Talos F200X G2 instrument operating at an accelerating voltage of 200 kV. The EDS analysis was acquired using a 20 μs dwell time per pixel. TEM sample preparation: the electrode material was peeled off the electrode and dispersed in ethanol, and drops of the suspension were deposited on carbon-coated copper grids and dried in air. XRD analysis was performed using a Rigaku smartLab SE X-ray diffractometer equipped with a Cu Kα source (small angle X-ray scattering data collected on a Bruker D8 advance diffractometer was used to the deviation) with a step size of 0.01°. FT-IR spectroscopy was determined from SHIMAD IRTracer-100. XPS analysis was performed through Thermo Scientific Nexsa G2, equipped with a monochromatic Al Kα X-ray source. Electrochemical measurements were displayed on the electrochemical workstation (CHI760E, CHI Instruments, Shanghai, China). EXAFS data were collected from the Shanghai Synchrotron Radiation Facility (SSPF), China. EPR spectra were recorded at 293 K with a Bruker EMXnano259 spectrometer, operated at 9.62 GHZ with 12.59 mW power and modulation at 100 kHz/1 G. TN and TOC were measured by Multi N/C 3100 TOC/TN analyzer.

### Pre-treatment of carbon cloth

The carbon cloth was soaked and sonicated in anhydrous ethanol and Milli-Q water, respectively for minutes, then dried in an oven at 80 °C before use.

### Preparation of P-CDP@CC cathode

A mixture of β-cyclodextrin (0.200 g, 0.176 mmol), 3,4,5,6-Tetrafluorophthalonitrile (0.100 g, 0.500 mmol), and K_2_CO_3_ (0.300 g, 2.170 mmol) was loaded into a Pyrex tube, and the above-prepared carbon cloth was added to the tube. The tube was flushed with Ar gas for 5 min, then an anhydrous THF/DMF mixture (9:1 v/v, 8 mL) was added and the tube was purged with Ar for an additional 20 min, and the mixture was placed at 85 °C for 48 h. The loaded carbon cloth was washed with N, N-Dimethylformamide (DMF), water, and tetrahydrofuran (THF). Finally, the product was collected and dried at 80 °C overnight under vacuum and named as P-CDP@CC.

### Preparation of TiO_2_ NRAs photoanode

The preparation of the TiO_2_ nanotube arrays (NRAs) electrode followed the electrochemical anodic oxidation methods according to the previous report^51^. First, the surface of the Ti mesh was treated in isopropyl alcohol (30 mL), ethanol (30 mL), and water (30 mL) for ultrasonic cleaning for about 20 min. Then, the Ti mesh (2 × 2 cm^2^) was electrochemically oxidized at 60 V for 2 h in a mixed solution containing 0.278 g NH_4_F, 49 mL ethylene glycol, and 1 mL Milli-Q water. Finally, the Ti mesh was calcined in the tube furnace at 450 °C for 3 h to obtain the TiO_2_ NRAs photoanode.

### Preparation of Ni/TiO_2_ NRAs photoanode

The electrodeposition was carried out in a standard three-electrode electrochemical cell (100 mL) containing TiO_2_ NRAs as the working electrode, a graphite rod as the counter electrode, and an Ag/AgCl as the reference electrode^52^. The electrolyte bath contained 0.01 M NiCl_2_·6H_2_O and 0.1 M H_3_BO_3_. The constant potential electrodeposition was then carried out at −1.5 V vs. Ag/AgCl. The optimized deposition time of Ni has been determined to be 130 s. After deposited, the work electrode was carefully withdrawn from the electrolyte, rinsed with water and ethanol, and dried in air. The mass of Ni deposited on the TiO_2_ were determined by ICP-OES.

### Electrochemical uranyl extraction

The electrochemical uranyl extraction experiments were measured by the electrochemical workstation (Chenhua Shanghai, China) in a single-chamber reactor (50 mL) with a three-electrode system. P-CDP@CC (1×1 cm^2^, 3.03 mg/cm^2^), Ag/AgCl (6 × 140 mm) and graphite rod (6×90 mm) were used as the working electrode, reference electrode and graphite rod, respectively. The electrochemical uranyl extraction performances were measured in an aqueous solution containing 50 ppm of uranyl and 0.1 M of NaNO_3_, and the pH was moderated by adding HNO_3_ and NaOH. The influence of pH of the solution (2 to 9), loading amounts (1.38 to 4.18 mg/cm^2^), initial concentration of uranyl (from 10 to 100 ppm), and interfering ions (Ca^2+^, Mg^2+^, Sr^2+^, Al^3+^, Fe^3+^, and SiO_3_^2^^−^) on the electrochemical uranyl extraction performance were carried out in detail. The concentrations of interfering ions were measured by ICP-OES. To evaluate the extraction capability of the P-CDP@CC electrode, constant voltage (−0.693 V vs Ag/AgCl) was applied for the steady extraction of uranyl. In the long-term uranyl extraction test, after one run of electro-extraction, the used P-CDP@CC electrode was regenerated by elution with 0.1 M Na_2_CO_3_ (8 mL) applied reverse bias 0.3 V vs. Ag/AgCl for 20 min. The resulted P-CDP@CC electrode was then reused for another electro-extraction experiment. The residual uranyl concentration in the electrolyte was determined by Arsenazo Ⅲ with UV-vis spectrophotometry at 650 nm. CV tests were performed in 0.1 M NaNO_3_ electrolyte with and without 50 ppm uranyl over a potential range of −2 V to 0.6 V vs. Ag/AgCl with scan rates of 10 mV/s, 20 mV/s, 40 mV/s, 50 mV/s, 60 mV/s, 80 mV/s, and 100 mV/s.

### Photoelectrochemical urea degradation

The PEC performance of the prepared photoanode was evaluated in a single-chamber reaction (100 mL) under irradiation of a 300 W xenon lamp (Perfectlight CEL-HXF300). The prepared Ni/TiO_2_ photoanode (2 × 2 cm^2^), graphite rod (6 × 90 mm), and Ag/AgCl electrode (6 × 140 mm) were used as working electrode, counter electrode, and reference electrode, respectively, which were connected to an electrochemical workstation (CHI 760). The working and counter electrode was spaced 1.5 cm, and the light source was 10 cm away from the electrolytic cell. The reaction solution was 40 mL of 0.1 M NaNO_3_ aqueous solution containing 100 ppm urea, and the pH was moderated by adding HNO_3_ and NaOH. PEC urea degradation reaction was performed at 25 °C by holding the illuminated photoanode at 0.8 V vs. Ag/AgCl for 4 h. The urea concentration was measured by p-dimethylaminobenzaldehyde with UV-vis spectrophotometry at 422 nm^53^. Meanwhile, at required intervals, the reaction solution was sampled and diluted, and the concentrations of TN and TOC were measured by the elemental analyzer method (Temperature: 800°C, Oxygen flow rate: 160 mL/min). LSV curves were measured at a scan rate of 10 mV/s, ranging from −0.79 V to 1.07 V vs. Ag/AgCl under irradiation of a 300 W xenon lamp. LSV measurements were conducted with 90% iR compensation. The photocurrent-time curves were investigated under irradiation at an applied potential of 0.8 V vs.Ag/AgCl. EIS were obtained under open-circuit potential with the frequency range from 10^-2^ to 10^4 ^Hz, and Mott-Schottky (MS) measurements were carried out at 1000 Hz. CV tests were performed at the non-faradic potential range of 0.271 ~ 0.371 V vs.Ag/AgCl with the scan rates of 5 mV/s, 10 mV/s, 20 mV/s, 40 mV/s, 60 mV/s, 80 mV/s, and 100 mV/s.

### PEC measurement in low-level radioactive organic wastewater

PEC measurements were performed in a standard two-electrode system with a single-compartment reactor under irradiation of a 300 W xenon lamp. The Ni/TiO_2_ (2 × 2 cm^2^) and P-CDP@CC (2 × 2 cm^2^) were used as photoanode and cathode, respectively, which were connected to a DC power source (SW17600SL 5 A). The detailed parameters of the low-level radioactive organic wastewater were shown in Supplementary Table 5. Uranyl extraction and urea degradation were investigated at 25 °C by holding the illuminated photoanode at 3.0 V for 8 h in low-level radioactive organic wastewater. The used P-CDP@CC cathode with uranyl uploaded was transferred into 0.1 M Na_2_CO_3_ elution solution, and a reverse bias of 0.8 V was applied for 20 min. The used Ni/TiO_2_ photoanode was successively cleaned in ethanol and water for minutes each, followed by drying in air before further use.

## Supplementary information

Supplementary Information file

Transparent Peer Review file

## Data Availability

The data supporting the findings of this study are available in the article and Supplementary Information files or available from the corresponding authors upon request. The numerical values for the data shown in all the Figures are provided in the attached Source Data file. Source data are provided with this paper (10.6084/m9.figshare.30022465).
