# Supplementary material for: Coupling uranyl upcycling with photoelectrochemical urea degradation for radioactive organic wastewater management
Source: Nat Commun. 2026 Apr 17;17:5329. doi: 10.1038/s41467-026-72145-w (PMC13272934; doi:10.1038/s41467-026-72145-w)
Supplement: Supplementary file 1 — Supplementary Information file [file 41467_2026_72145_MOESM1_ESM.pdf]

# Supplementary Information for

# Coupling uranyl upcycling with photoelectrochemical urea degradation for radioactive organic wastewater management

Huihui Jin[1,2], Zewen Shen[1,2], Chumin Yan[1,2], Yang Liu[1], Yingying Tian[2], Hongliang Bao[3], Zhuoyu Ji[1], Yezi Hu[1*], Guixia Zhao[1*], Xiangke Wang[1*], Xiubing Huang[2*]

[1]College of Environmental Science and Engineering, North China Electric Power University, Beijing 102206, P. R. China

[2]Beijing Advanced Innovation Center for Materials Genome Engineering, Beijing Key Laboratory of Function Materials for Molecule & Structure Construction, School of Materials Science and Engineering, University of Science and Technology Beijing, Beijing 100083, P. R. China

[3]Key Laboratory of Interfacial Physics and Technology, Shanghai Institute of Applied Physics, Chinese Academy of Sciences, Shanghai 201800, P. R. China

* Corresponding authors: huyz@ncepu.edu.cn (Yezi Hu), guixiazhao@ncepu.edu.cn (Guixia Zhao), xkwang@ncepu.edu.cn (Xiangke Wang), xiubinghuang@ustb.edu.cn (Xiubing Huang)

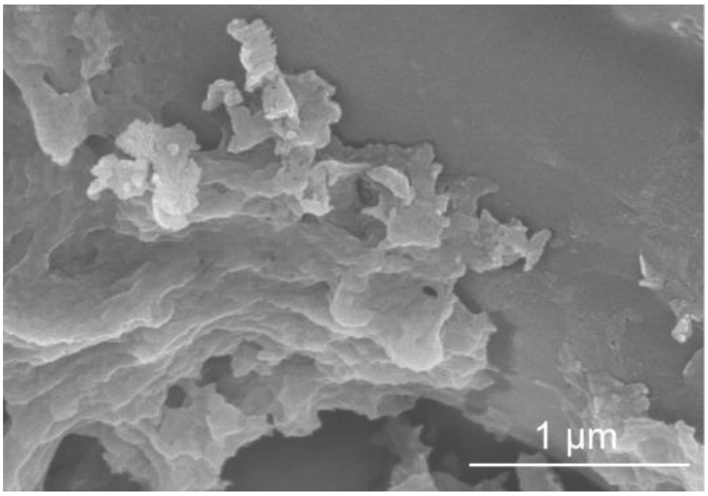


**Supplementary Fig. 1 |** SEM image of P-CDP@CC.

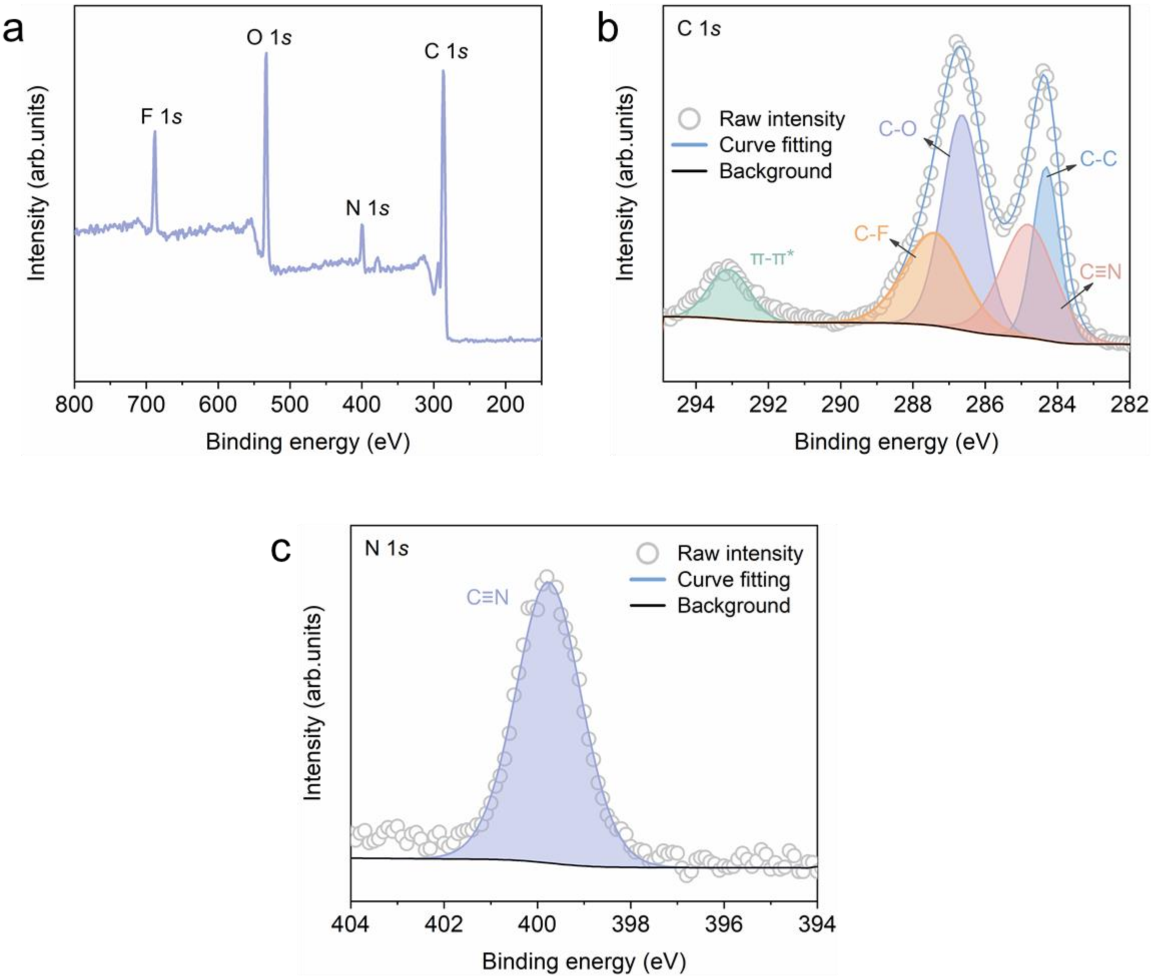


**Supplementary Fig. 2 | The surface chemical state analysis of P-CDP@CC.** XPS spectrum of (a) wide scan and high resolution spectrum of (b) C 1*s*,and (c) N 1*s* for P-CDP@CC.

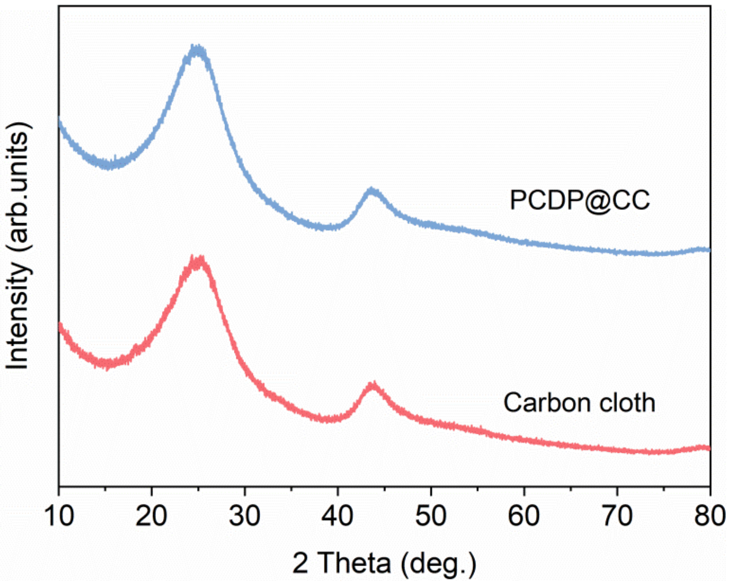


**Supplementary Fig. 3 |** XRD pattern of P-CDP@CC and carbon cloth

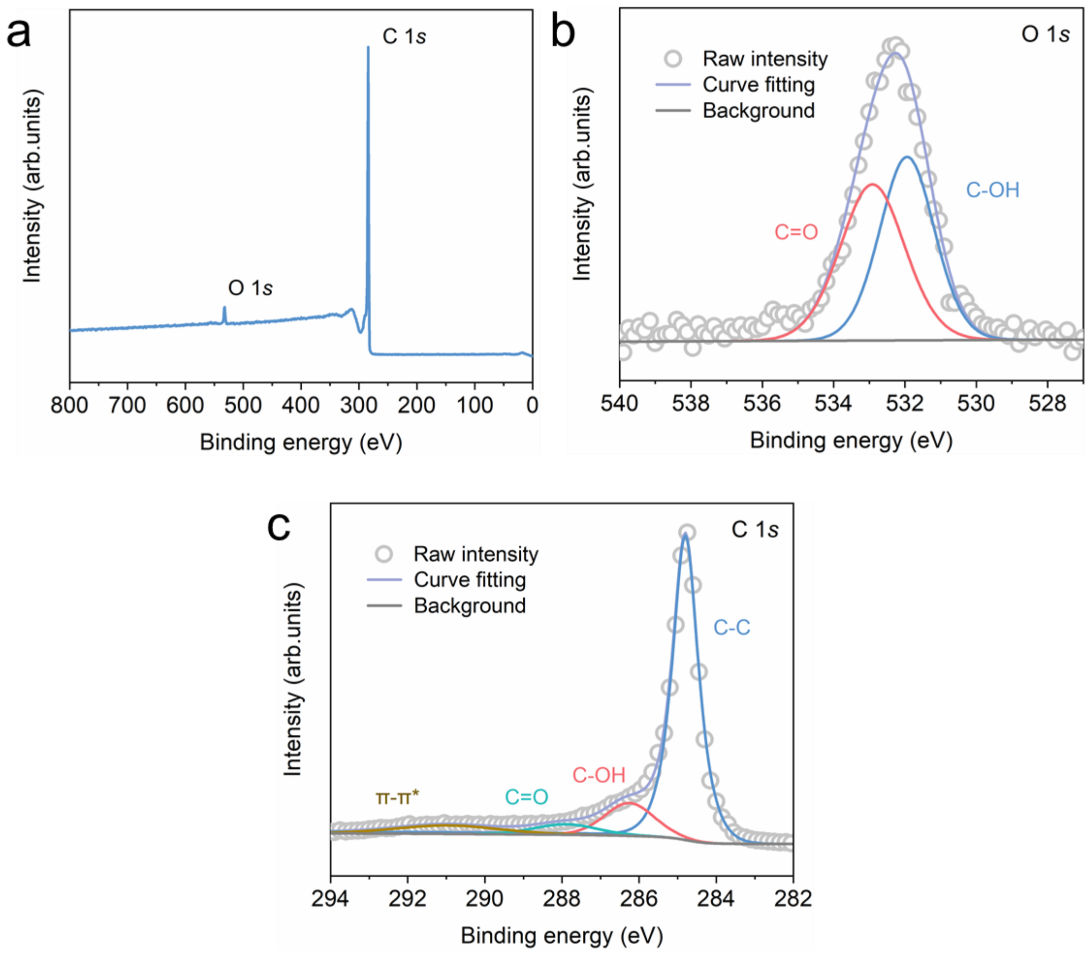


**Supplementary Fig. 4 | The surface chemical state analysis of carbon cloth.** (a) XPS survey scan of carbon cloth. (b) O 1*s* and (c) C 1*s* spectrum of carbon cloth.

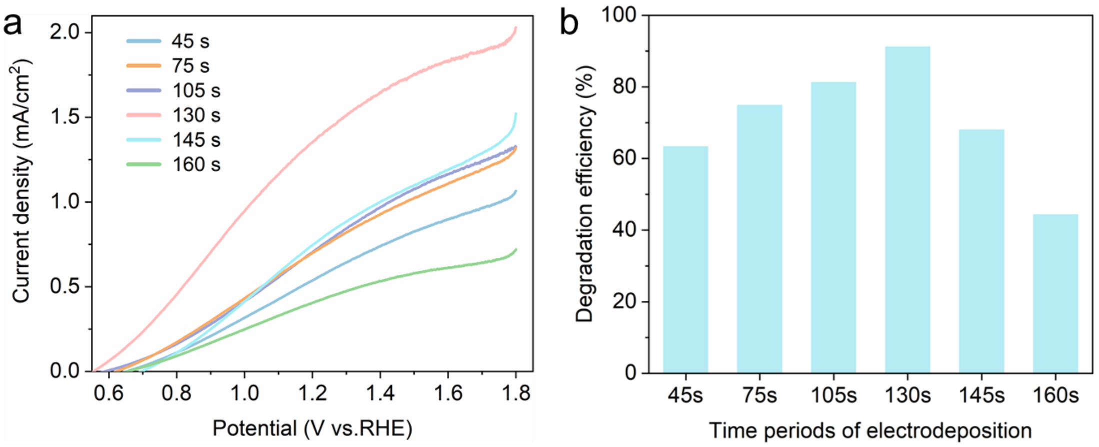


**Supplementary Fig. 5 | Urea degradation performance of Ni/$TiO_2$ in different electrodeposition periods.** (a) LSV curves of the Ni/$TiO_2$ electrode by electrodeposition in different periods. (b) Degradation efficiency of urea on the electrode by electrodeposition in different periods.

The results showed the optimal urea degradation of 91.11 % at the deposition time of 130 s with Ni loading content of 0.045 %, thus electrodeposited time was set to 130 s for further studies.

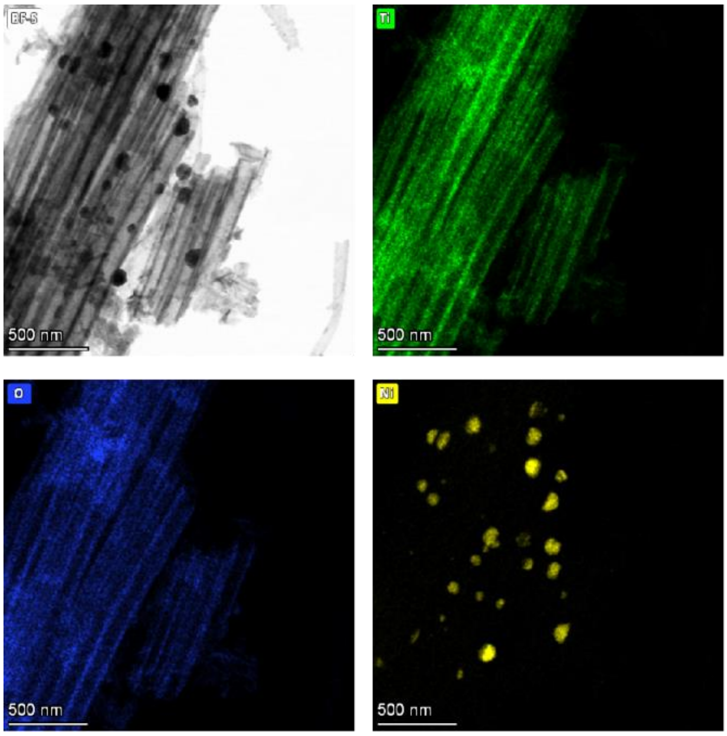


**Supplementary Fig. 6 |** EDS analysis of $Ni/TiO_2$.

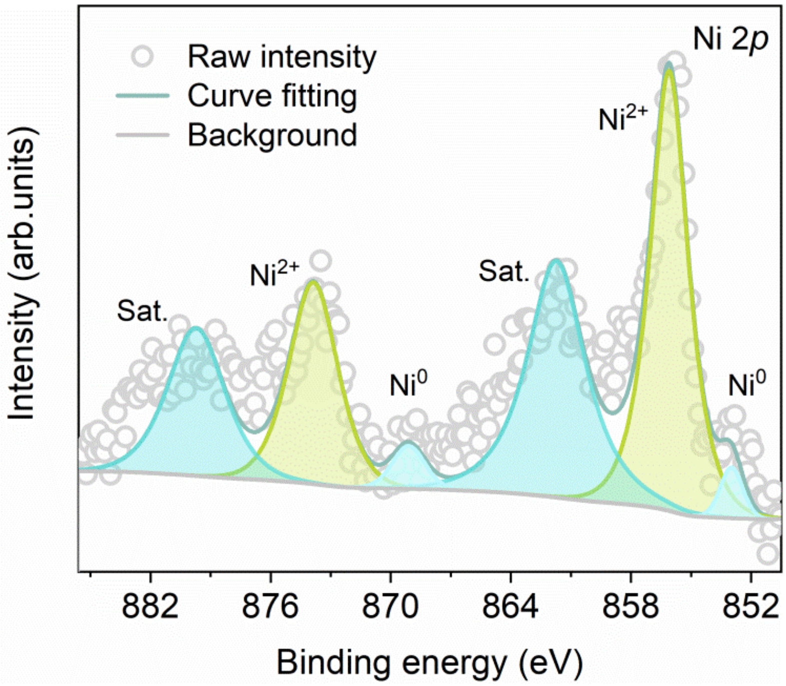


**Supplementary Fig. 7 |** High-resolution XPS spectra of Ni 2*p* signal for Ni/$TiO_2$.

The Ni 2*p* signals confirm the presence of both $Ni^0$ and $Ni^{2+}$ on the surface of the Ni/$TiO_2$. This dual presence is not surprising given the spontaneous oxidation of metallic Ni to $Ni^{2+}$ upon exposure to air.

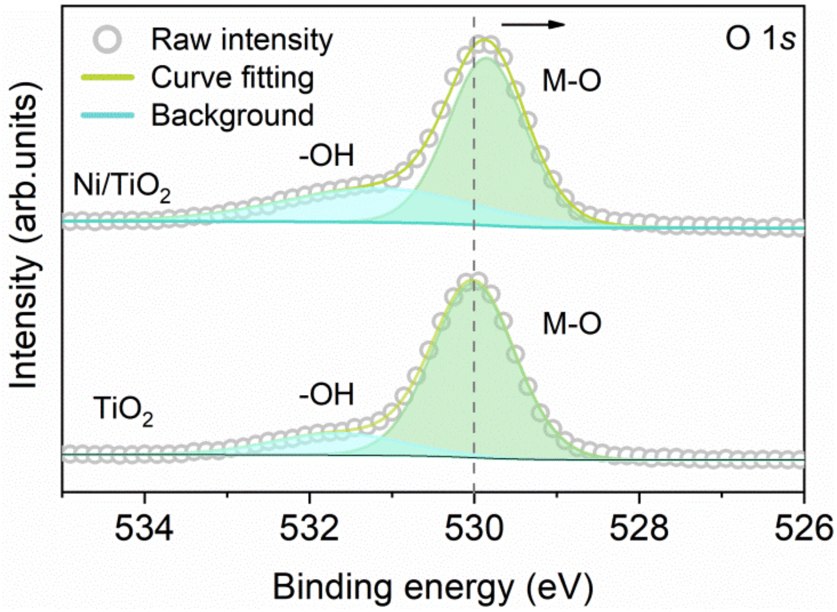


**Supplementary Fig. 8 |** High-resolution XPS spectra of O 1*s* signal for $Ni/TiO_2$.

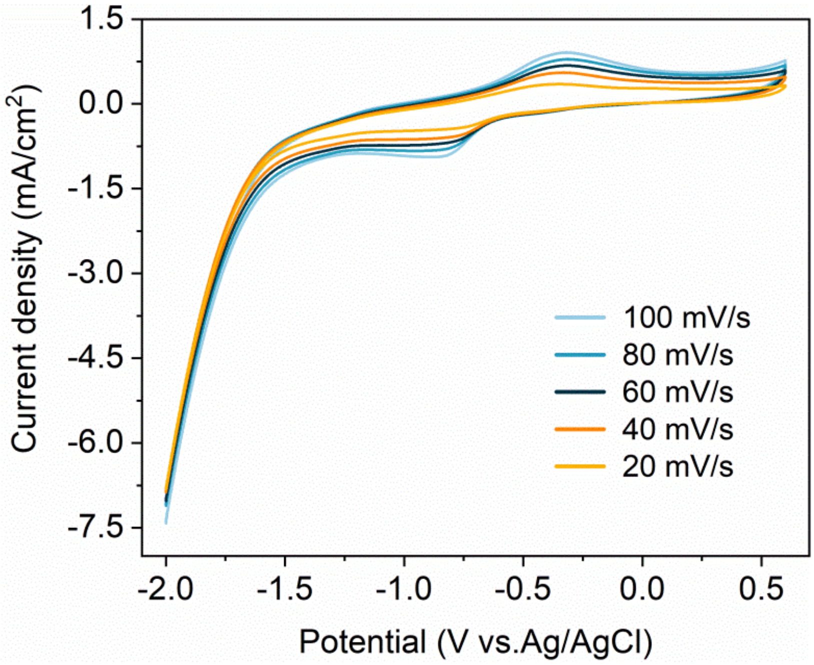


**Supplementary Fig. 9 |** CV curves under P-CDP@CC (Scan rate: 20~100 mV/s)

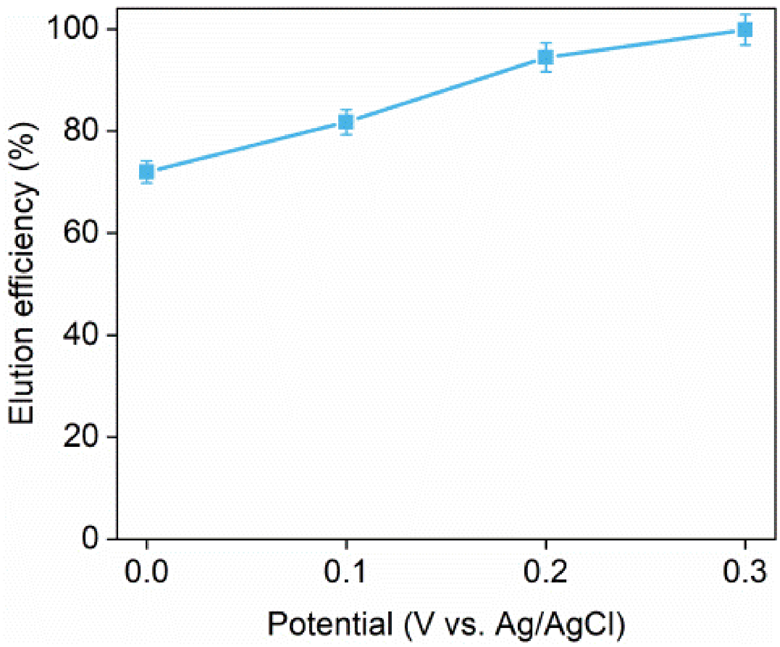


**Supplementary Fig. 10 |** Uranyl elution efficiency on the used P-CDP@CC electrode at different potentials. Error bars represent standard deviation of three measurements.

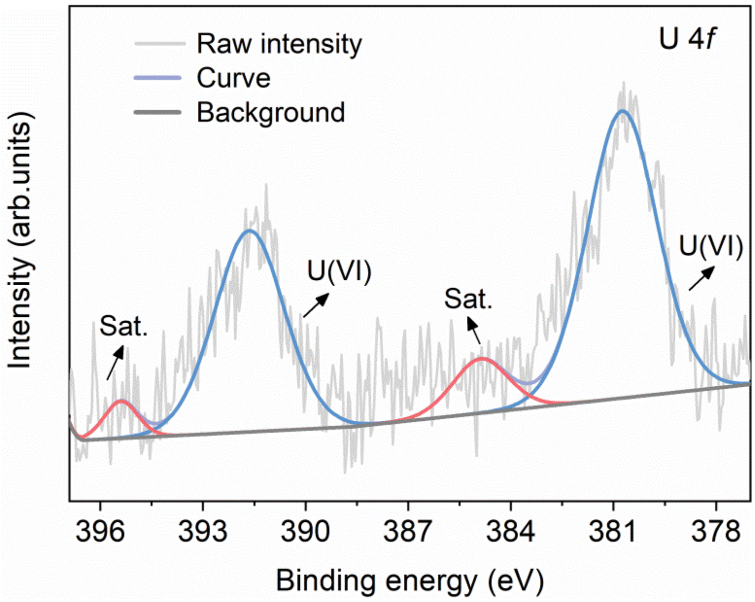


**Supplementary Fig. 11 |** U 4*f* spectra of P-CDP@CC electrode after elution process.

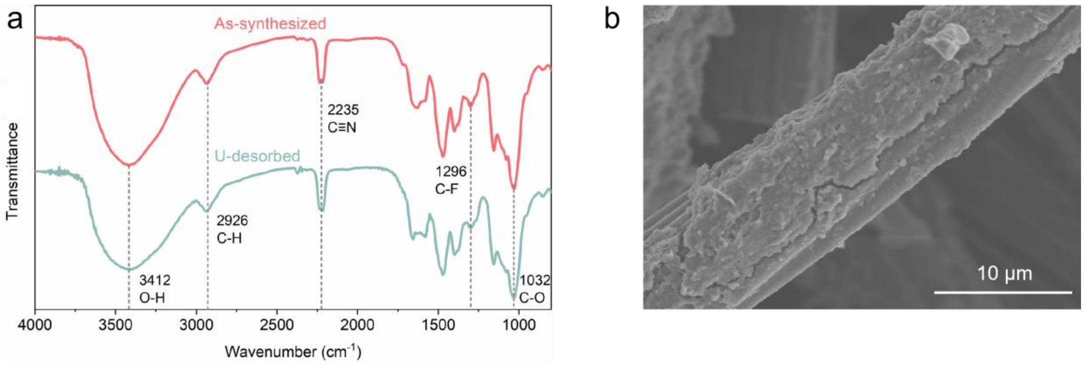


**Supplementary Fig. 12 | The stability of P-CDP@CC electrode.** (a) FTIR (b) and SEM image of the P-CDP@CC electrode after cycling.

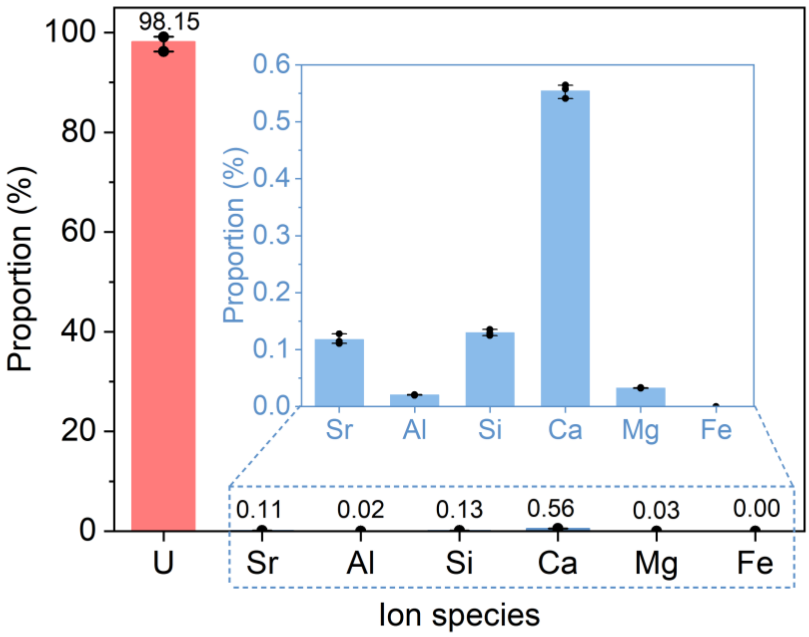


**Supplementary Fig. 13 |** The proportion of uranyl among the metal species in the precipitate after electronic extraction. The illustration is an enlarged view of the selected region. Error bars represent standard deviation of three measurements.

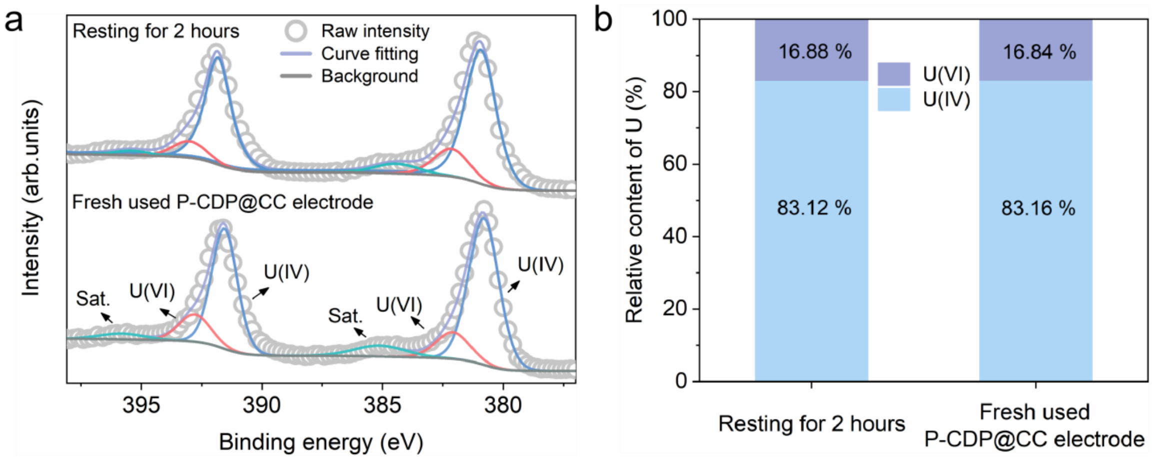


**Supplementary Fig. 14 | The valence state of the used P-CDP@CC electrode.** (a) XPS spectra of U 4*f* for the used P-CDP@CC electrode before and after resting. (b) The content of different valence states of uranyl (U(VI) and U(IV)) for the used P-CDP@CC electrode before and after resting.

Compared with the fresh used P-CDP@CC electrode, XPS results showed no obvious change of uranium (IV) ratio on the surface of the electrode after resting for 2 hours, demonstrating that the cathodic product can be remained quite well, even long-time resting under open cell. Furthermore, it's also confirmed that only with positive bias during the elution process, the extracted tetravalent uranyl dioxide on P-CDP@CC electrode was found to be oxidized back to soluble U(VI). Therefore, we believe that in our electrochemical reduction process, re-oxidation of the cathodic product is negligible.

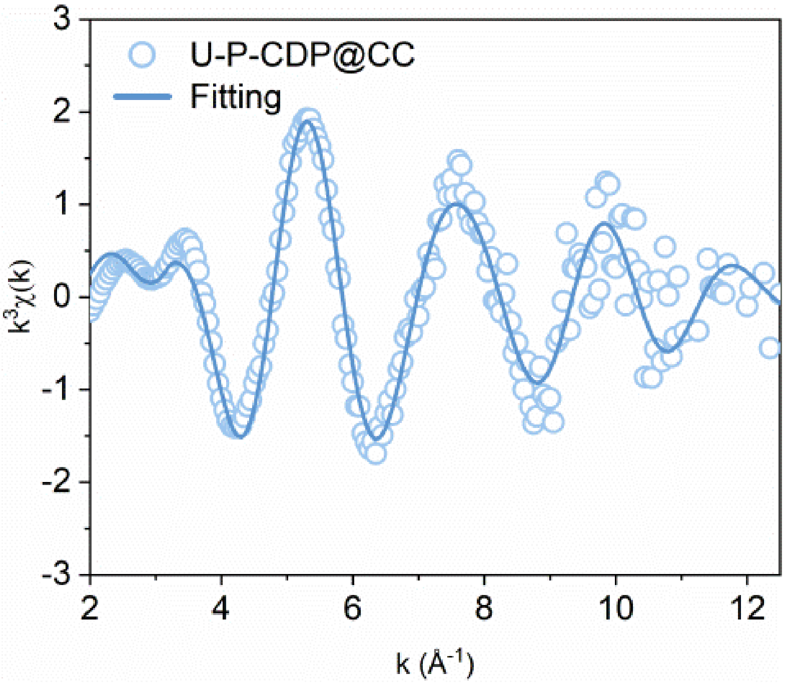


**Supplementary Fig. 15 |** Corresponding k-space fitting curve for the P-CDP@CC after uranyl extraction.

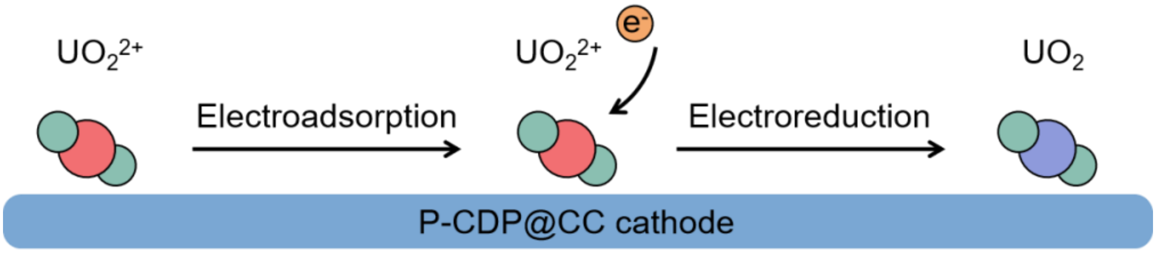


**Supplementary Fig. 16 |** A diagram of the P-CDP@CC electrode reaction mechanism.

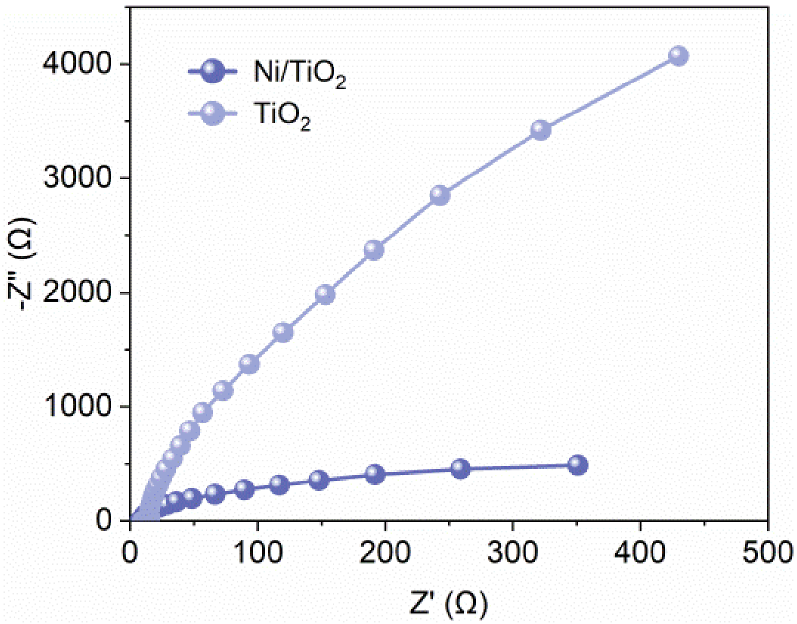


**Supplementary Fig. 17 |** Nyquist plots of $TiO_2$ and Ni/$TiO_2$ photoanodes recorded in 0.1 M $NaNO_3$ under dark at open-circuit voltage.

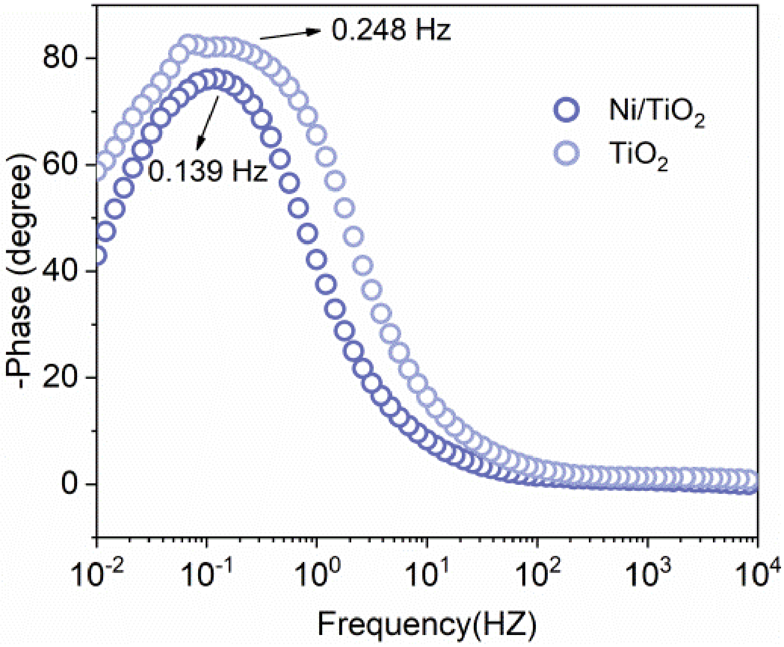


**Supplementary Fig.18 |** Bode plots of $TiO_2$ and Ni/$TiO_2$ photoanodes under irradiation of a 300 W xenon lamp at open-circuit voltage.

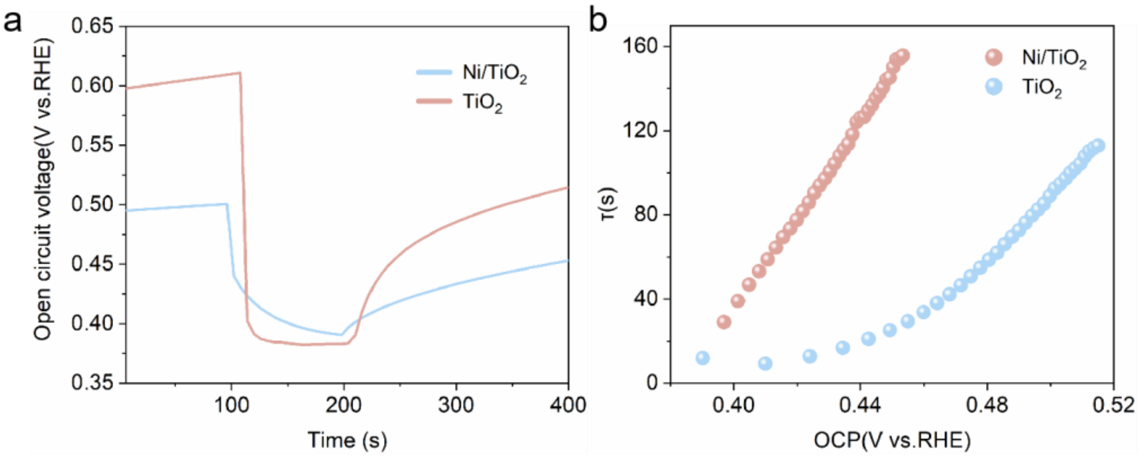


**Supplementary Fig. 19 |** (a) Open-circuit voltage decay (OCVD) of $TiO_2$ and Ni/$TiO_2$. (b) The electron lifetime for $TiO_2$ and Ni/$TiO_2$.

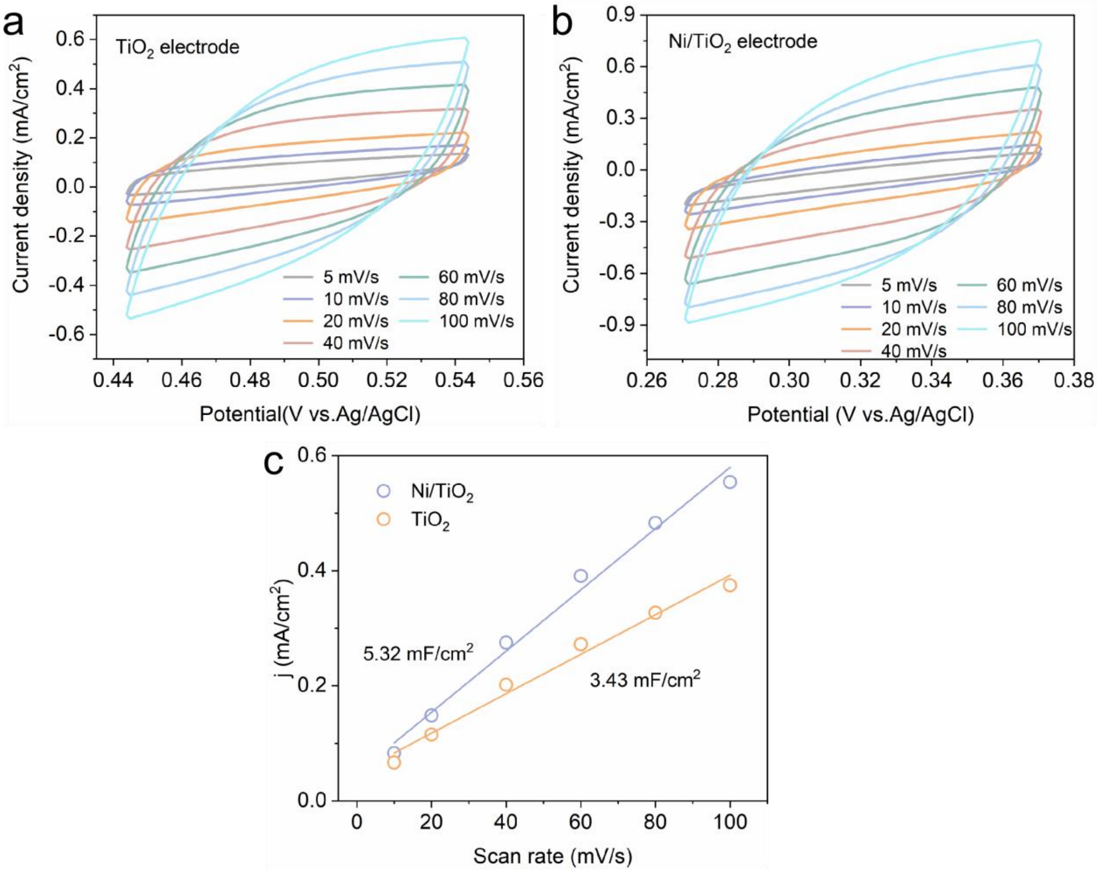


**Supplementary Fig. 20 |** (a, b) CV curves recorded at different scan rates in the non-faradaic capacitance current range. (c) The double-layer capacitance ($C_{dl}$) for $TiO_2$ and Ni/$TiO_2$. ($TiO_2$: $R^2$=0.98, Ni/$TiO_2$: $R^2$=0.99).

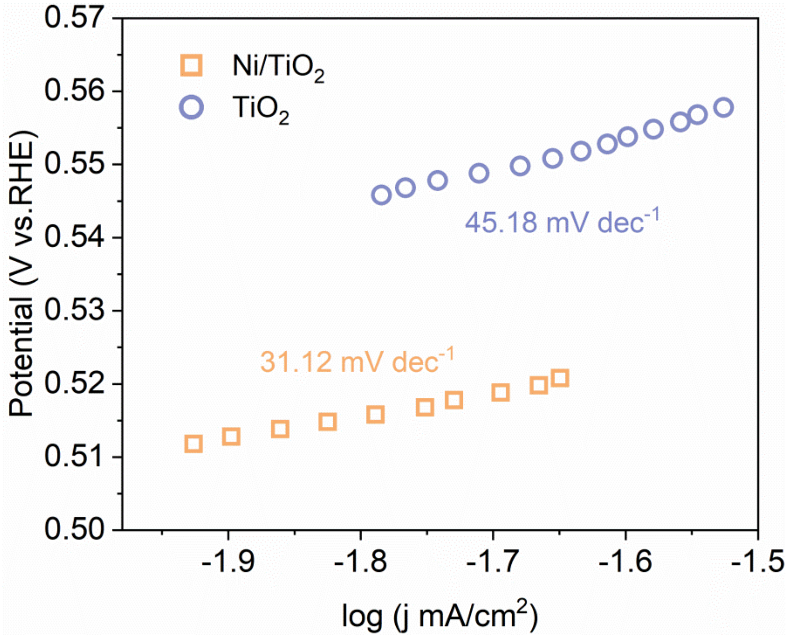


**Supplementary Fig. 21 |** Tafel slopes of $TiO_2$ and Ni/$TiO_2$.

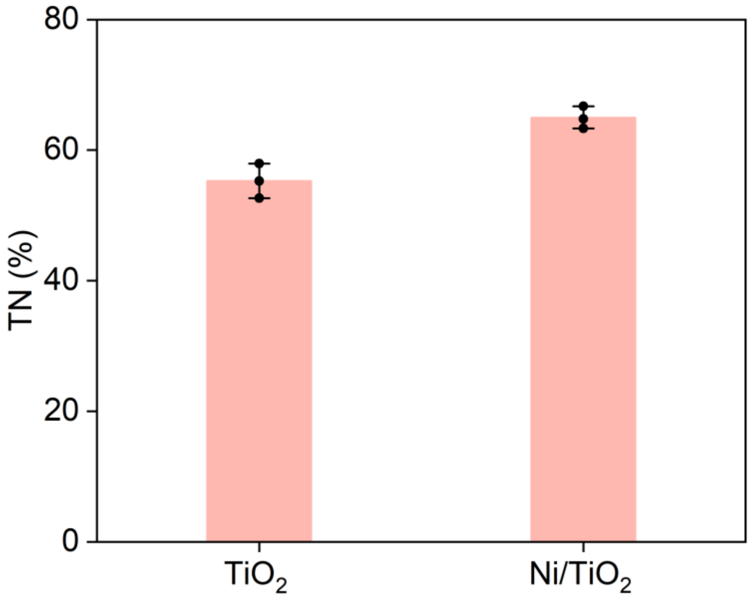


**Supplementary Fig. 22 |** N-total removal during the PEC degradation processes on $TiO_2$ and Ni/$TiO_2$. (Potential: 0.8 V vs. Ag/AgCl, 300 W xenon lamp). Error bars represent standard deviation of three measurements.

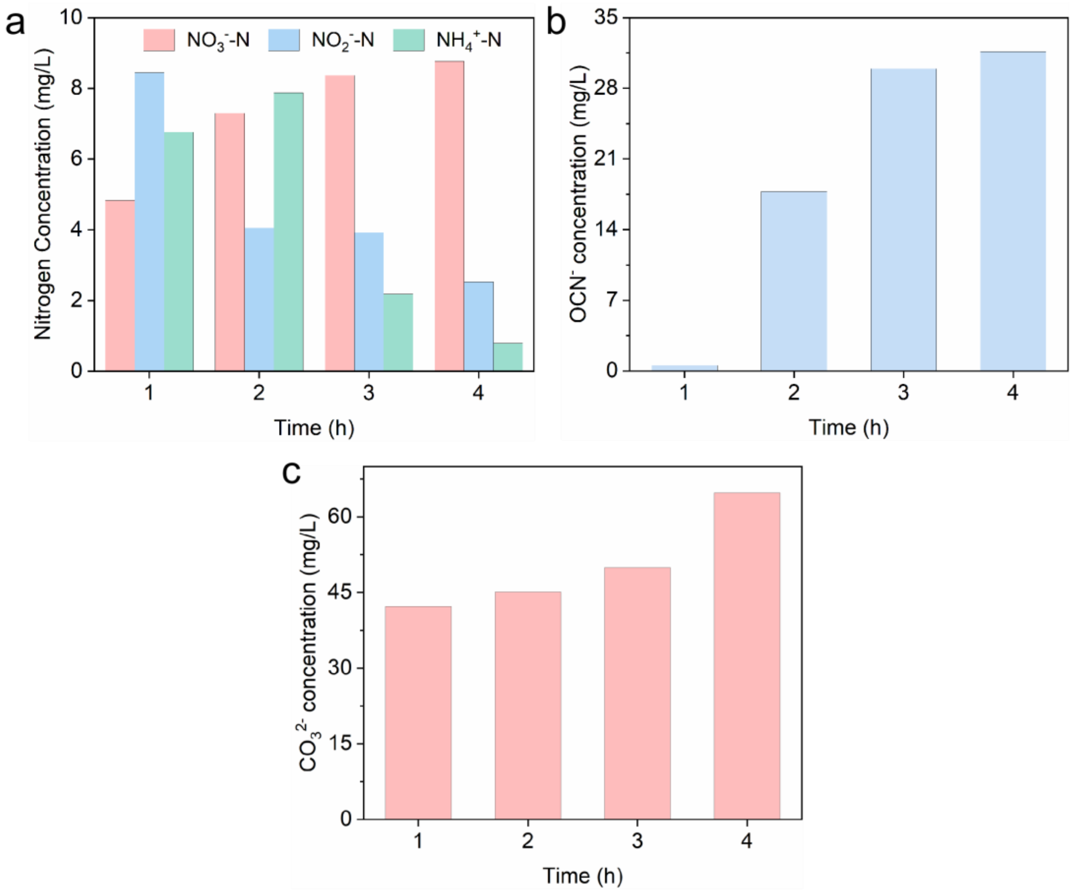


**Supplementary Fig. 23 | Concentration of produced nitrogen species and carbon species.** (a-b) The concentration of produced nitrogen species for Ni/$TiO_2$. (b) The concentration of produced carbon species for Ni/$TiO_2$.

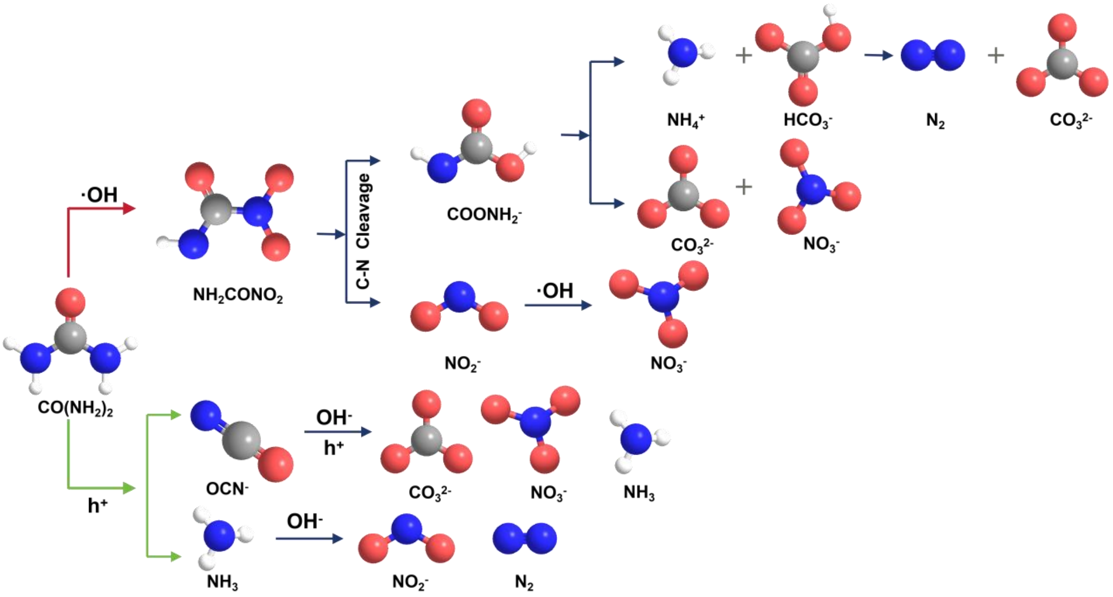


**Supplementary Fig. 24 |** A diagram of the Ni/$TiO_2$ photoanode reaction mechanism.

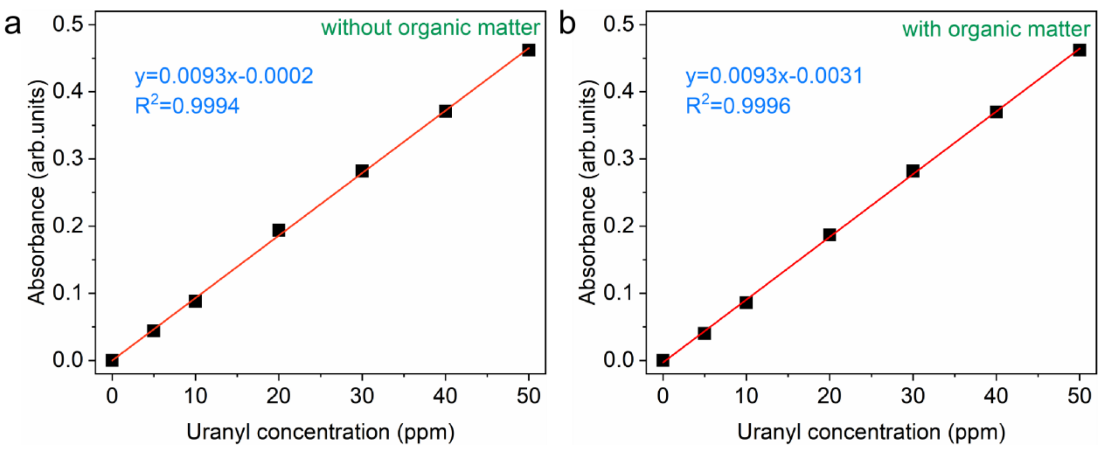


**Supplementary Fig. 25 |** The standard curves of uranyl concentration-absorbance (a) without organic matter and (b) with organic matter.

The standard curves of uranyl both in the presence and in the absence of organic matter have been compared. Notably, the $R^2$ values for the curves are 0.9996 and 0.9994, respectively, indicating a high degree of correlation and accuracy. Obviously from the comparison, it is confirmed that the quantitative analysis of uranyl remains unaffected by the presence of organic matter.

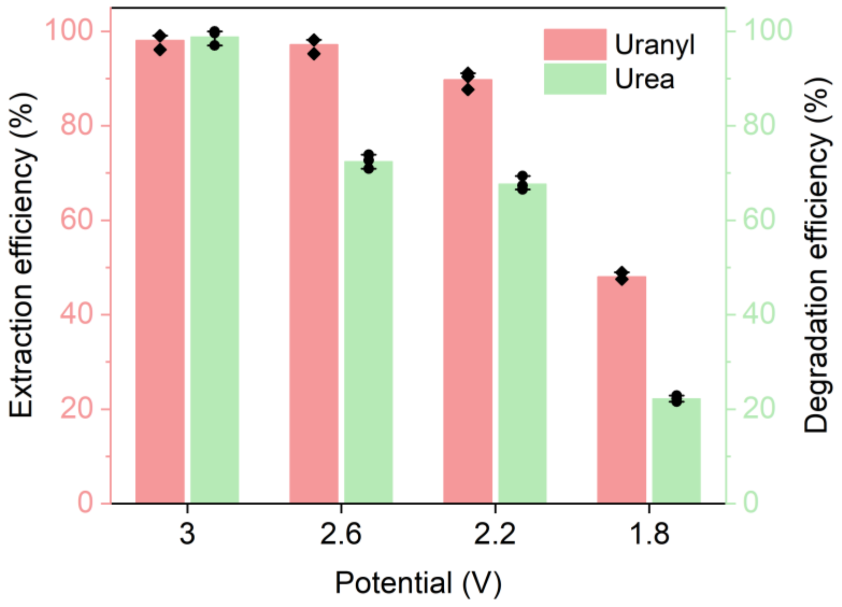


**Supplementary Fig. 26 |** Uranyl extraction efficiency and urea degradation efficiency in low-level radioactive organic wastewater at different cell voltages. Error bars represent standard deviation of three measurements.

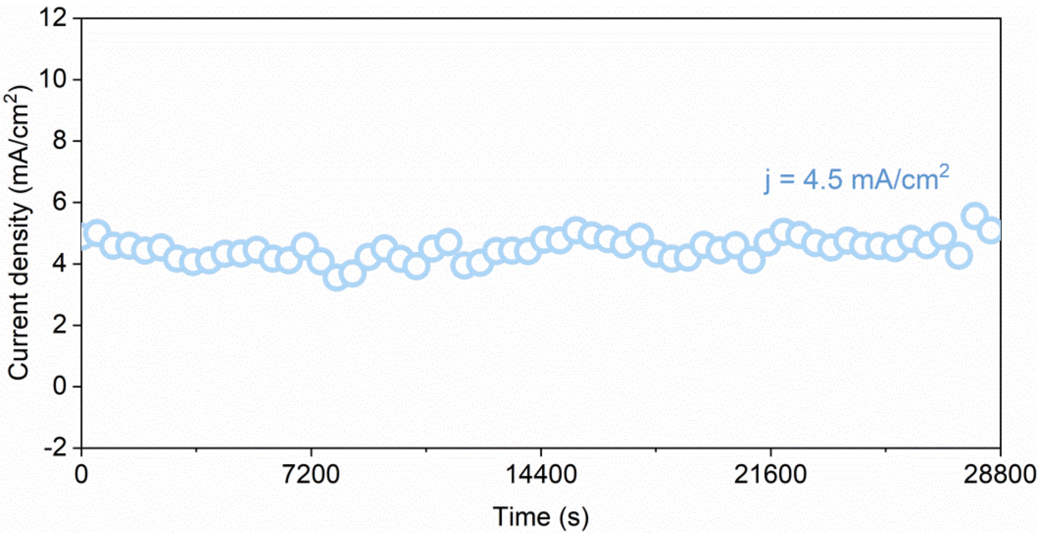


**Supplementary Fig. 27 |** Chronoamperometry curve of the coupling system recorded at 3 V for 8 h.

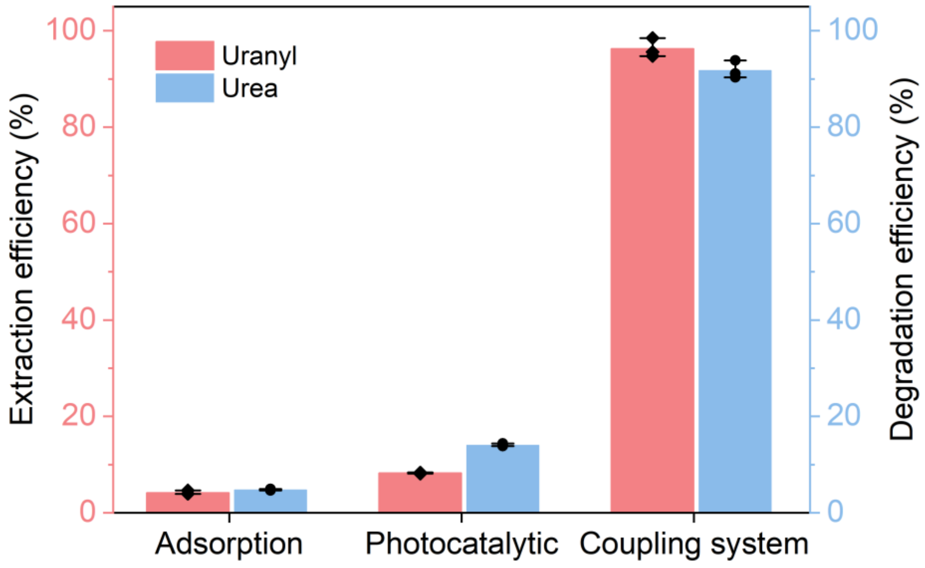


**Supplementary Fig. 28 |** Comparison of uranyl extraction efficiency with the adsorption, photocatalytic and coupling system. Error bars represent standard deviation of three measurements.

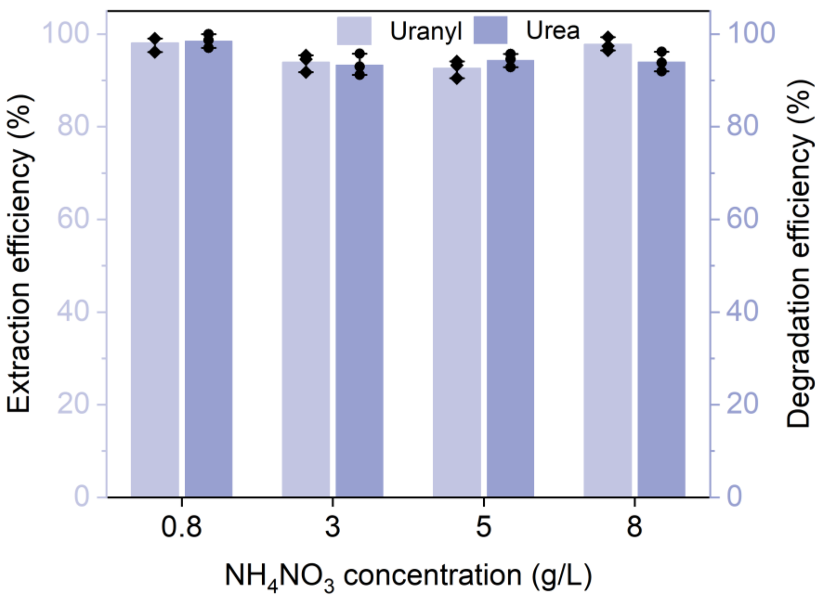


**Supplementary Fig. 29 |** Uranyl extraction efficiency and urea degradation efficiency in coupling system as the $NH_4NO_3$ concentrations varied from 0.8 g/L to 8 g/L. Error bars represent standard deviation of three measurements.

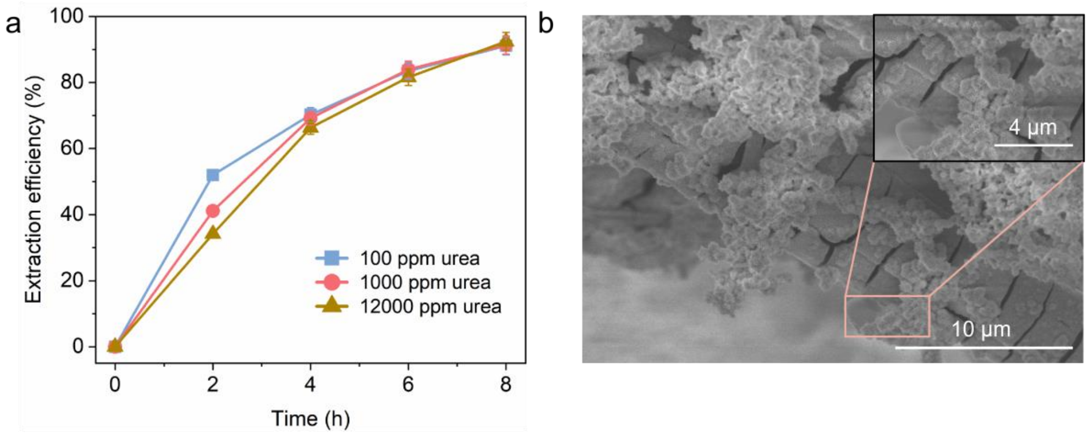


**Supplementary Fig. 30 | Uranyl extraction performance of P-CDP@CC with different urea concentrations. (a)** Uranyl extraction efficiency with the urea concentration varied from 100 ppm to 12000 ppm.(b) SEM image of P-CDP@CC after uranyl extraction with 12000 ppm urea. The illustration is an enlarged view of the selected region.

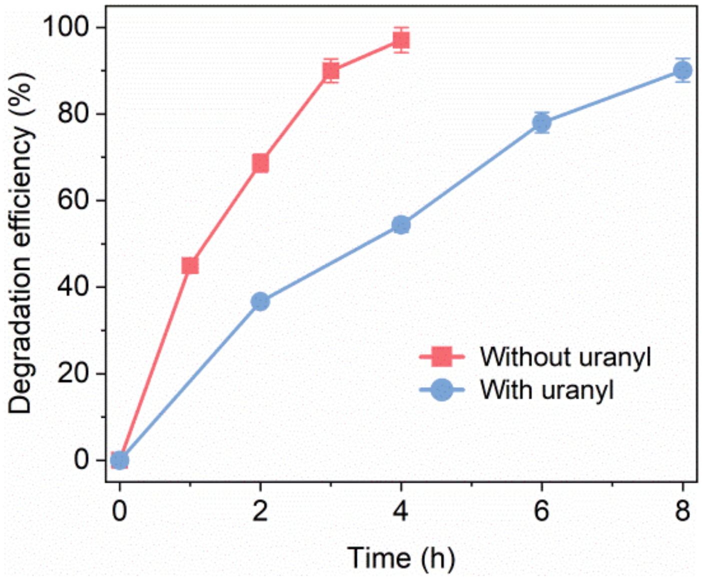


**Supplementary Fig. 31 |** Urea degradation efficiency of Ni/$TiO_2$ in the absence and presence of 50 ppm uranyl. Error bars represent standard deviation of three measurements.

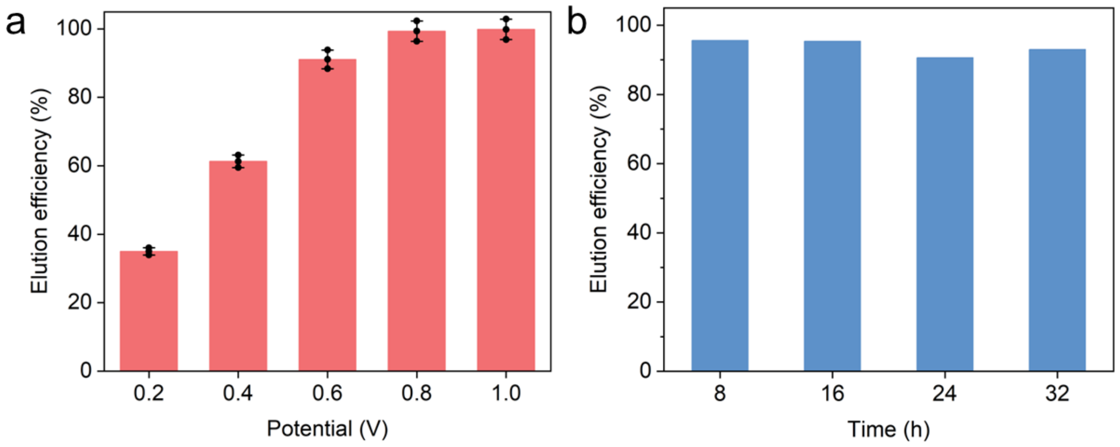


**Supplementary Fig. 32 | The elution performance of P-CDP@CC.** (a) Uranyl elution efficiency on P-CDP@CC at different reverse bias. Error bars represent standard deviation of three measurements. (b) Desorption cycle performance of P-CDP@CC.

With the reverse bias increased from 0.2 to 0.8 V, the uranyl elution efficiency improved from 35 % to 99.34 %. When the applied potential exceeded 0.8 V, the elution efficiency slightly increased. Considering the cost and to avoid water oxidation, we employed 0.8 V as the elution potential.

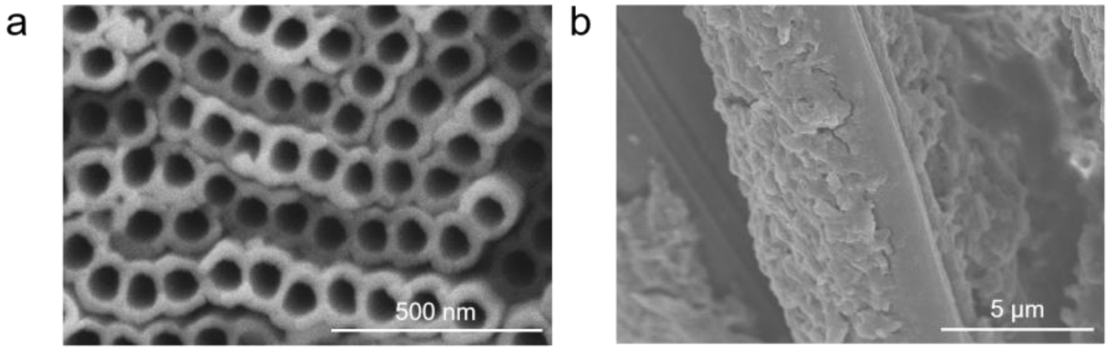


**Supplementary Fig. 33 | The stability of photoanode and cathode.** SEM images of (a) Ni/$TiO_2$ photoanode and (b) P-CDP@CC cathode after cycling tests.

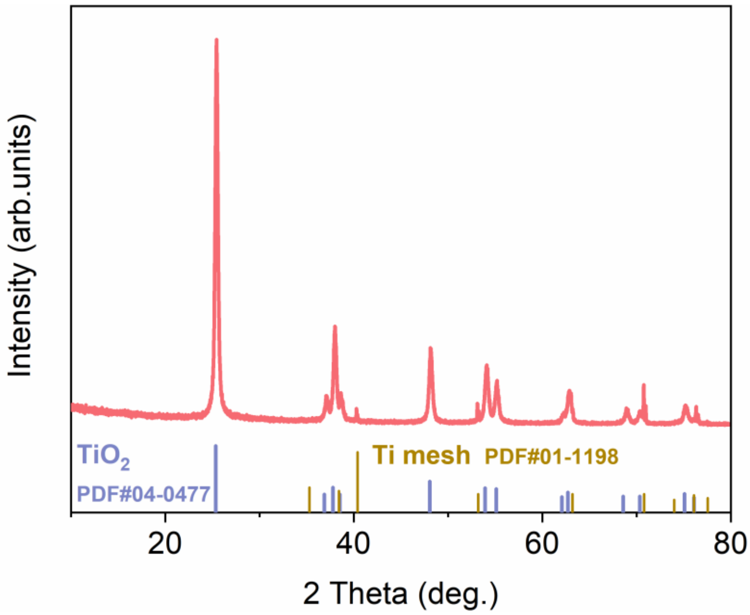


**Supplementary Fig. 34 |** XRD patterns of Ni/$TiO_2$ after cycling tests.

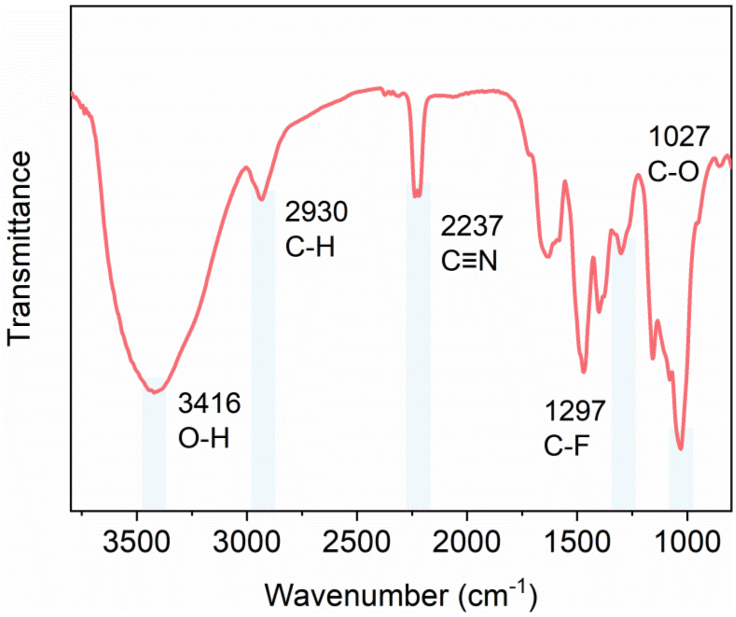


**Supplementary Fig. 35 |** FTIR spectra of P-CDP@CC after cycling tests.

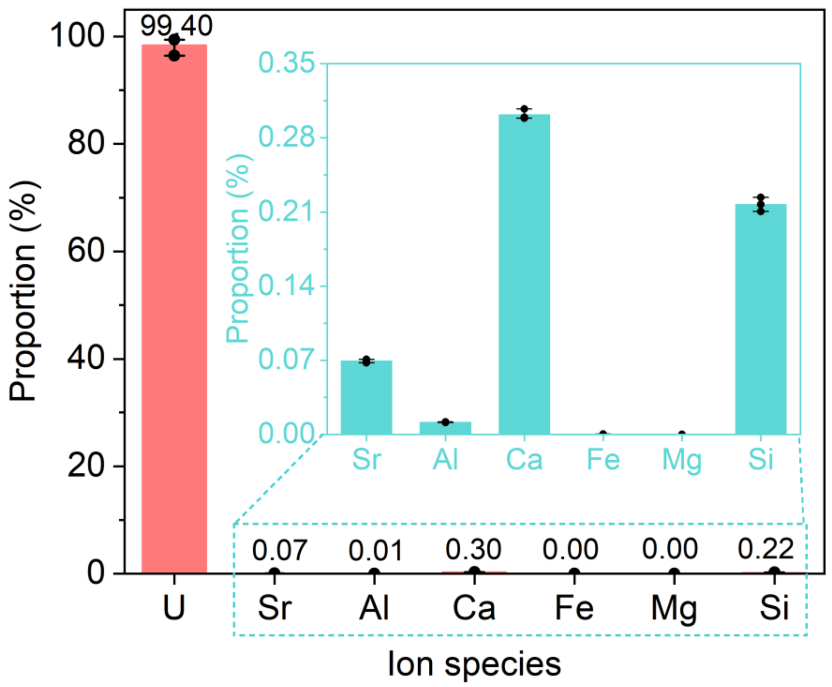


**Supplementary Fig. 36 |** The proportion of uranyl among the metal species in the precipitate after electronic extraction from complicated wastewater. The illustration is an enlarged view of the selected region. Error bars represent standard deviation of three measurements.

**Supplementary Table 1 |** Quantitative results for Ni loading content at different deposition times.

| Electrodeposition time | Loading amount |
|---|---|
| 45 s | 0.019 % |
| 75 s | 0.031 % |
| 130 s | 0.045 % |
| 160 s | 0.050 % |

**Supplementary Table 2 |** EXAFS fitting results of P-CDP@CC electrode after uranyl extraction.

| Sample | Path | CN | R/Å | $\sigma^2$/Å$^2$ | ΔE/eV | R-factor |
|---|---|---|---|---|---|---|
| P-CDP@CC electrode after uranyl extraction | U-$O_{ax}$ | 1.532 | 1.808 | 0.007 | 9.813 | 0.019 |
| | U-$O_{eq}$ | 1.724 | 2.185 | 0.015 | 9.813 | |
| | U-$O_{eq}$ | 1.915 | 2.410 | 0.011 | 9.813 | |

CN is the coordination number. R is the distance between absorber and backscatter atoms. $\sigma^2$ is the Debye-Waller factor. ΔE is the inner potential correction. R-factor is residual factor. U-$O_{ax}$ and U-$O_{eq}$ represent axial coordination and plane coordination, respectively.

**Supplementary Table 3 |** The $f_{max}$ and $\tau_e$ of $TiO_2$ and Ni/$TiO_2$.

| Electrode | $f_{max}$ (Hz) | $\tau_e$(ms) |
|---|---|---|
| $TiO_2$ | 0.248 | 641.754 |
| Ni/$TiO_2$ | 0.139 | 1144.999 |

**Supplementary Table 4 |** Urea Degradation efficiency of Ni/$TiO_2$ photoanode in the presence of various radical scavengers.

| Quencher | Degradation efficiency (%) |
|---|---|
| No addition | 91.109 |
| $Na_2SO_3$ | 0.392 |
| tert-butanol (TBA) | 46.038 |

**Supplementary Table 5 |** The detailed constituents of low-level radioactive organic wastewater.

| Constituents | Concentration (ppm) |
|---|---|
| U(VI) | 75.32 |
| Urea | 100 |
| $NH_4NO_3$ | 800~8000 |
| Ca | 15.271 |
| Mg | 4.977 |
| Si | 2.156 |
| Sr | 0.370 |
| Al | 0.370 |
| Fe | 0.094 |
| pH | 9 |

**Supplementary Table 6 |** Cost analysis results

| Electrode | | Photoanode Ni/$TiO_2$ | Cathode P-CDP@CC |
|---|---|---|---|
| Ligands cost (USD/g or USD/$cm^2$) | 3,4,5,6-Tetrafluorophthalonitrile ($10^{-3}$ USD/g) | / | 3.9 |
| | β-cyclodextrin ($10^{-3}$ USD/g) | / | 0.8 |
| | Ti sheet ($10^{-3}$ USD/$cm^2$) | 0.121 | / |
| Extraction/Degradation capacity ($10^{-3}$ g) | | 18.848 | 3.538 |
| Energy consumption (USD/g) | | 0.074 | 0.00005 |
| Cost for uranyl extraction /urea degradation (USD/g) | | 0.006 | 0.002 |
| Overall cost (USD/g) | | 0.082 | |

**Supplementary Table 7 |** A comparison of the performance and cost of the bipolar coupling system with those of the previously reported system.

| | | | Performance | | | |
|---|---|---|---|---|---|---|
| | Materials | Composition | Uranyl extraction efficiency/ adsorption capacity | Organic matter removal efficiency | Cost | Ref. |
| Adsorption Method | AL-PEI/GMS | 20 mg/L U | 95.6 % | / | 150 USD/ kg | 1 |
| | PPN-3 | 20 mg/L U+ 15 mg/L HA | 325.41 mg/g | / | 200 USD/ kg | 2 |
| | MCP-5 | 20 mg/L U+ 15 mg/L HA | 304.31 mg/g | / | 2360 USD/ kg | 3 |
| Electrochemical method | ZrN/copper foam | 23.7 mg/L U | 82.4 % | / | 85.7 USD/ kg | 4 |
| | G-CCA/PAO | 0.838 mg/L U | 97.98 % | / | 3150 USD/ kg | 5 |
| | PTFE | 0.259 mg/L U | 82.6 % | / | 747.9 USD/ kg | 6 |
| | PAECOF-AO @CC | 50 mg/L U | 93.80% | / | 500 USD/ kg | 7 |
| | Photoanode：Ni/$TiO_2$ Cathode:P-CDP@CC | 50 mg/L U+ 100 mg/L urea | 99.08 % (653.99 mg/g) | Nearly 100 % | 82 USD/ kg | This work |

## Supplementary References


1. Guo, L. *et al*. Ultrafast uranium recovery from high-salinity nuclear wastewater using amine-functionalized lignin microspheres. *J. Clean. Prod.* **388**, 136006 (2023).
2. Liu, Y. *et al.* Facile and scalable synthesis of functionalized hierarchical porous polymers for efficient uranium adsorption. *Water Res.* **257**, 121683 (2024).
3. Liu, Y. *et al.* Functionalized hydrogen-bonded organic superstructures via molecular self-assembly for enhanced uranium extraction. *J. Hazard. Mater.* **464**, 133002 (2024).
4. Wang, X. *et al.* Anion‐Exchange Membrane Electrolysis for Efficient Uranium Extraction from Saline Wastewater. *Adv. Mater.* **37**, e08705 (2025).
5. Wang, Y. *et al.* Unlocking the potential of cotton-derived carbon aerogel for uranium extraction from real radioactive wastewater: A path to amidoxime and polyguanidine modification. *Chem. Eng. J.* **519**, 165635 (2025).
6. Dai, Z. *et al*. Efficient extraction of uranium(VI) in aqueous solution by contact-electro-catalysis. *Chem. Eng. J.* **502**, 157893 (2024).
7. Jin, H. *et al.* Electrochemical upcycling of uranyl from radioactive organic wastewater with a self-standing covalent-organic framework electrode. *Nat Commun.* **16**, 3574 (2025).
