# Supplementary material for: Coupling uranyl upcycling with photoelectrochemical urea degradation for radioactive organic wastewater management
Source: Nat Commun. 2026 Apr 17;17:5329. doi: 10.1038/s41467-026-72145-w (PMC13272934; doi:10.1038/s41467-026-72145-w)
Supplement: Supplementary file 2 — Transparent Peer Review file [file 41467_2026_72145_MOESM2_ESM.pdf]

nature portfolio

Peer Review File

Coupling uranyl upcycling with photoelectrochemical urea degradation for radioactive organic wastewater management

Corresponding Author: Professor Xiubing Huang

This file contains all reviewer reports in order by version, followed by all author rebuttals in order by version.

Version 0:

Reviewer comments:

Reviewer #1

(Remarks to the Author)
In this manuscript, the authors introduced a new strategy for concurrent urea degradation and uranyl extraction from complicated wastewater matrix in a single-compartment electrolysis cell. This work innovatively proposed a photoelectrolysis method to reduce energy consumption via substitution of OER with UOR, avoiding the inefficient uranyl extraction and urea degradation from low-level radioactive organic wastewater, achieving a uranyl extraction efficiency of 99.08 % and urea degradation efficiency of nearly 100 % in a single-compartment electrolysis cell. Overall, it is a fundamentally interesting and important study, which will unlock the concurrent uranium resource recovery and organic pollutant treatment from a complicated wastewater matrix. However, there are still a few insufficient investigations and explanations from my perspective as listed following.
1、In Fig.2e, the authors have to make more detailed explanation for the decrease in extraction performance with pH 3 and pH 4.
2、The authors fabricated a self-standing P-CDP@CC electrode; thus, the authors are suggested to further discuss the interaction between polyarylether-based polymer and carbon cloth.
3、The authors loaded Ni nanoparticles onto the surface of TiO2 by electrodeposition. What is the Ni content on the Ni/TiO2? How does the author control Ni loading amount?
4、In the coupling system, why did the author apply the reverse bias of 0.8V during the elution process? The authors should make an explanation.
5、If the presence of uranyl from a complex wastewater affects the urea degradation in the coupling system? To prove these points, the authors have to add an additional controlled test.
6、The authors analyzed uranyl content after reaction in a complicated organic wastewater matrix. If the presence of organic matter would affect the quantitative analysis of uranyl? The authors should check the standard curve and compare the standard curves of uranyl with and without organic matter.
7、There are numerous errors of detail in the text, for example, XRD characterizes TiO2 as anatase, while XPS characterizes as rutile; in Fig. 2f, the authors should clearly state the optimal voltage for further studies; in the description of Cdl, the slope of TiO2 and Ni/TiO2 doesn't match the Supplementary Fig. 17c. Please double check the whole text.
8、To clarify that such a strategy is more promising for scale-up application, the authors should further compare the performance and cost of this work with other published works.

Reviewer #2

(Remarks to the Author)
This study reports a photoelectrochemical (PEC) system based on a Ni nanoparticle-modified TiO_2 photoanode and a P-CDP@CC cathode for simultaneous uranyl ion extraction and urea degradation in a single-compartment cell. The work tackles a relevant environmental issue by integrating resource recovery with pollutant removal, in line with circular economy principles. While the reaction kinetics are commendable, the underlying concept of uranium extraction itself is not novel at all, as it has been previously documented as follows.
1. “Simultaneously reducing UO22+ and abating organic pollutants by a self-driven photoelectrocatalytic system with urea modified carbon fibre cathode”, Chem. Eng. J. Volume 524, 169470
2. “Simultaneous removal and recovery of uranium from aqueous solution using TiO2 photoelectrochemical reduction method”, J. Radioanal. Nuclear Chem., Volume 313, pages 59–67, (2017)
This should be more suitable for a more specialized journal. Nevertheless, the following points require clarification and

stronger justification:

1. A mechanistic explanation should be provided for the notably lower extraction efficiency observed at pH = 3.
2. The treatment performance for both uranyl extraction and urea degradation should be assessed under acidic conditions representative of real uranium-containing effluents, to better demonstrate practical applicability.
3. The single-compartment cell design raises a fundamental concern regarding possible re-oxidation of the cathodic product. The manuscript should include a clear mechanistic discussion and supporting evidence showing how this issue is avoided in the present system.
4. The considerable gap between nearly complete urea degradation (~100%) and lower TOC/TN removal (~64%) suggests accumulation of intermediate products. Further analysis to identify these intermediates and propose a detailed degradation pathway is needed.
5. The rationale for the specific urea concentration used in the coupled system should be explained. In addition, the effect of varying urea concentrations on uranyl removal performance should be systematically investigated to elucidate the interaction between the two processes.
6. The potential interference of co-existing ions commonly found in real wastewater—particularly NO_3^- and NH_4^+—on both uranyl extraction and urea degradation has not been systematically evaluated.
7. The schematic of the "bipolar coupling system" (Fig. 5) is oversimplified. It should be improved to better illustrate the function of the material designs, such as the role of Ni nanoparticles on TiO_2 nanotubes in facilitating charge separation and enhancing urea oxidation kinetics.
8. The advantage of the coupled strategy should be underscored by direct performance comparisons with individual technologies, such as standalone adsorption or photocatalytic processes.
9. Experimental evidence is required to determine whether the valence state of U changes after dissolution of UO_2(s) in 0.1 M Na_2CO_3 solution, specifically whether eluted uranium is in the +4 or +6 state.
10. The study lacks a UO_2^{2+} selectivity assay. Selectivity against competing ions is essential in uranium extraction studies and must be included.
11. A comparison between the reduction of V and U should be provided, given that vanadium is a key competing cation in uranium extraction.
12. Appropriate correlation of distribution coefficients and an enrichment factor for uranium are currently lacking.
13. The system's performance should be discussed in the context of real conditions, where multiple uranium species, transition metals, and salts coexist. It is important to evaluate how the material would selectively extract trace U ions from a complex mixture of cations, especially when competing ions are present at much higher concentrations.
14. Unit formatting is inconsistent and must be standardized. Use standardized abbreviations throughout (e.g., "h" for hour, "mL" not "ml", "kg" not "Kg") in the text, figures, and tables.

Version 1:

Reviewer comments:

Reviewer #1

(Remarks to the Author)
The authors have addressed each of my questions one by one, and I currently have no further inquiries.

Reviewer #2

(Remarks to the Author)
My opinion remains the same. The concept of PEC system that simultaneously oxidizes organic pollutants and reduces uranyl ions has been well demonstrated in the literature. There is little novelty in this study. This should be more suitable to a specialized journal.

Open Access This Peer Review File is licensed under a Creative Commons Attribution 4.0 International License, which permits use, sharing, adaptation, distribution and reproduction in any medium or format, as long as you give appropriate credit to the original author(s) and the source, provide a link to the Creative Commons license, and indicate if changes were made.
In cases where reviewers are anonymous, credit should be given to 'Anonymous Referee' and the source.
The images or other third party material in this Peer Review File are included in the article's Creative Commons license, unless indicated otherwise in a credit line to the material. If material is not included in the article's Creative Commons license and your intended use is not permitted by statutory regulation or exceeds the permitted use, you will need to obtain permission directly from the copyright holder.
To view a copy of this license, visit https://creativecommons.org/licenses/by/4.0/

Point-by-Point Response to Reviewers' Comments

Reviewer #1 (Remarks to the Author):

In this manuscript, the authors introduced a new strategy for concurrent urea degradation and uranyl extraction from complicated wastewater matrix in a single-compartment electrolysis cell. This work innovatively proposed a photoelectrolysis method to reduce energy consumption via substitution of OER with UOR, avoiding the inefficient uranyl extraction and urea degradation from low-level radioactive organic wastewater, achieving a uranyl extraction efficiency of 99.08 % and urea degradation efficiency of nearly 100 % in a single-compartment electrolysis cell. Overall, it is a fundamentally interesting and important study, which will unlock the concurrent uranium resource recovery and organic pollutant treatment from a complicated wastewater matrix. However, there are still a few insufficient investigations and explanations from my perspective as listed following.

Response: We are grateful to the reviewer for taking the time to evaluate our work and greatly appreciate the positive comments and support of our work.

1、In Fig.2e, the authors have to make more detailed explanation for the decrease in extraction performance with pH 3 and pH 4.

Response: We appreciate the constructive comment from the reviewer. The pH not only controls uranyl species in solution, but also affects the protonation of the hydroxyl groups at electrode surface.

According to the literatures (*Nat. Commun. 2024, 15, 9124*; *Nat. Commun. 2025, 16, 2361*), uranyl mainly exists in the form of UO_2^{2+} at pH 3 and pH 4, while further alkaline condition (pH 5-8) would increase in the content ratio of the positively charged multinuclear hydroxide species of uranyl, such as $(UO_2)_2(OH)_2^{2+}$, $(UO_2)_3(OH)_5^{+}$,and $(UO_2)_4(OH)_7^{+}$, and at pH 8-10 the negatively charged uranyl species such as $(UO_2)_3(OH)_7^{-}$ and $UO_2(OH)_3^{-}$ are the prominent species.

Previous studies (*Nat. Commun. 2024, 15, 453*; *Nat. Sustain. 2022, 5, 71-80*) suggest that at low pH, hydroxyl groups on the P-CDP@CC electrode undergo protonation, leading to electrostatic repulsion between electrode surface and uranyl species. At higher pH, such repulsion effect is largely weakened since electrode surface tends to be electrically neutral charged.

Thus it turned out that with pH increasing, the uranyl extraction performance is increased as shown in **Fig. 2e**.

which is supposed due to the sluggish electron/ion transportation[38]. The pH not only controls uranyl species in solution, but also affects the protonation of the hydroxyl groups at electrode surface, thus the influence of pH on the electrolytic uranyl extraction over P-CDP@CC electrode was further evaluated. As illustrated in **Fig. 2e**, when pH is as low as 3.0, only 13 % of uranyl is extracted, attributing to the protonation of the hydroxyl groups on the P-CDP@CC electrode, leading to electrostatic repulsion between electrode surface and the uranyl cations. At pH 4.0, the extraction sharply improved to 58.13 %, and the optimal extraction of 96.99 % is obtained at pH 5.0. With pH increases to 6 and 9, the extraction remained consistently above 90%, which was supposed due to that the repulsion effect is largely weakened since electrode surface tends to be electrically neutral charged. Hence, pH value of the electrolyte was set to 5 for further studies. Increasing the applied

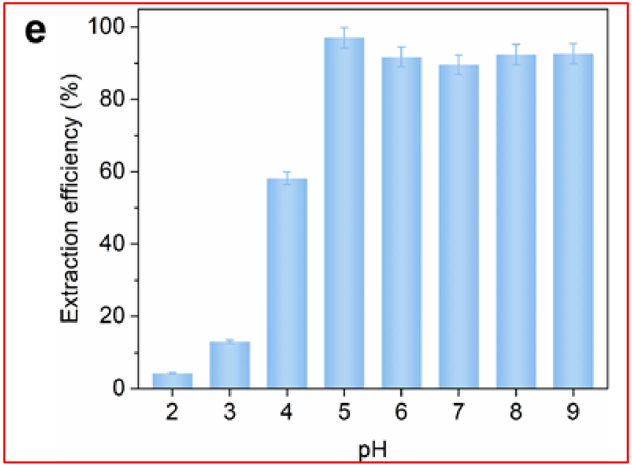

Fig. 2 | e Electrochemical extraction of uranyl on P-CDP@CC electrode under different pH conditions.

2、The authors fabricated a self-standing P-CDP@CC electrode; thus, the authors are suggested to further discuss the interaction between polyarylether-based polymer and carbon cloth.

Response: Thank you very much for the question. Owing to your constructive comment about the interaction between substrate and polyarylether-based polymer, we have supplemented the X-ray photoelectron spectroscopy (XPS) of carbon cloth to further check the surface functional groups. As shown in the split peak fitting spectra of O 1*s* and C 1*s*, there exist abundant C-OH and C=O groups

on the surface of carbon cloth (**Supplementary Fig.4**). Therefore, the possible interaction between polyarylether-based polymer and carbon cloth can be: (1) hydrogen bonds between C-OH groups on the surface of carbon cloth and OH groups on polyarylether-based polymer; (2) hydrogen bonds between C-OH groups on the surface of carbon cloth and F atoms from 3,4,5,6-tetrafluorophthalonitrile.

polymer (**Supplementary Fig. 3**). Thereafter, as shown in the split peak fitting spectra of O 1*s* and C 1*s*, there exist abundant C-OH and C=O groups on the surface of carbon cloth (**Supplementary Fig. 4**). Therefore, the possible interaction between polyarylether-based polymer and carbon cloth can be: (1) hydrogen bonds between C-OH groups on the surface of carbon cloth and OH groups on polyarylether-based polymer; (2) hydrogen bonds between C-OH groups on the surface of carbon cloth and F atoms from 3,4,5,6-tetrafluorophthalonitrile.

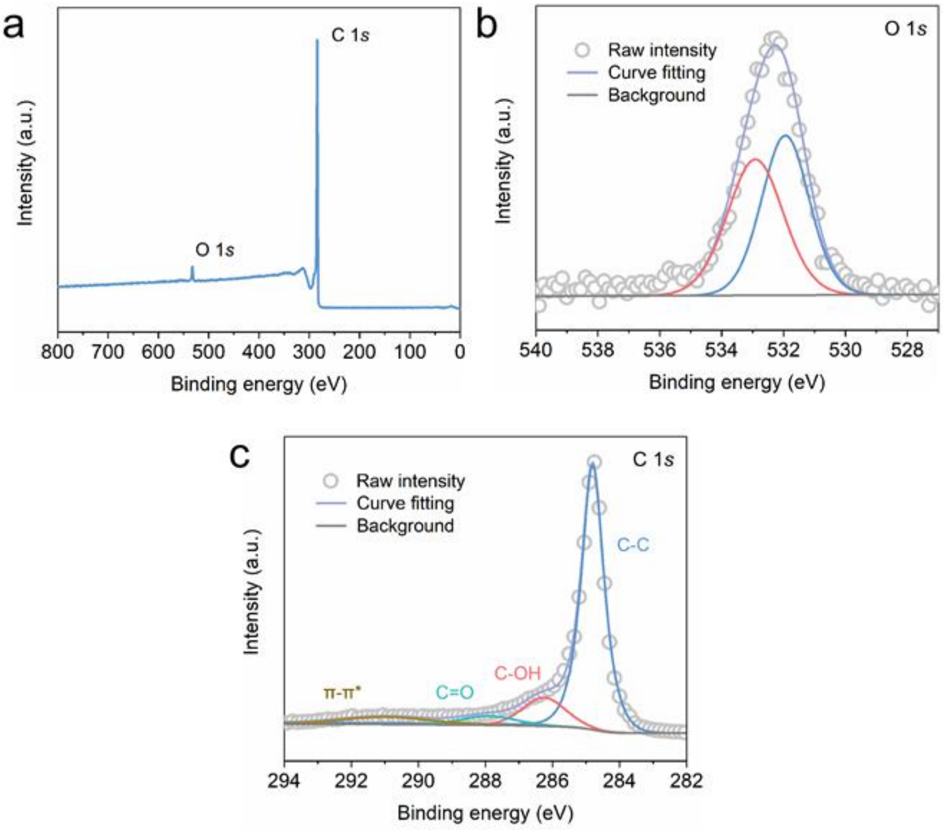

Supplementary Fig. 4 | (a) XPS survey scan of carbon cloth. (b) O 1*s* and (c) C 1*s* spectrum of carbon cloth.

3、The authors loaded Ni nanoparticles onto the surface of TiO_2 by electrodeposition. What is the Ni content on the Ni/TiO_2? How does the author control Ni loading amount?

Response: We appreciate the constructive comment from the reviewer. Inspired by your suggestion to explore the mass of Ni deposited on the TiO_2 during the electrodeposition, we collected the Ni/TiO_2 electrode at different deposition time for ICP-OES analysis (**Supplementary Table 1**). As shown in the following table, with various electrodeposition times increased from 45 s to 160 s for Ni loading, the content on Ni/TiO_2 improved from 0.019 % to 0.050 %. The optimal urea degradation of 91.11 % at the deposition time of 130 s with Ni loading content of 0.045 % (**Supplementary Fig. 5**), thus electrodeposited time was set to 130 s for further studies.

Supplementary Table 1 | Quantitative results for Ni loading content at different deposition time

Electrodeposition time	Loading amount
45 s	0.019 %
75 s	0.031 %
130 s	0.045 %
160 s	0.050 %

4、In the coupling system, why did the author apply the reverse bias of 0.8V during the elution process? The authors should make an explanation.

Response: We thank the reviewer for raising this issue and we are sorry for that in the initially submitted manuscript, we did not show the data about the influence of the reverse bias on uranyl elution. As shown in **Supplementary Fig.33a**, with the reverse bias increased from 0.2 to 0.8 V, the uranyl elution efficiency improved from 35% to 99.34%. When the applied potential exceeded 0.8 V, the elution efficiency slightly increased. Considering the cost and to avoid water oxidation, we employed 0.8 V as the elution potential.

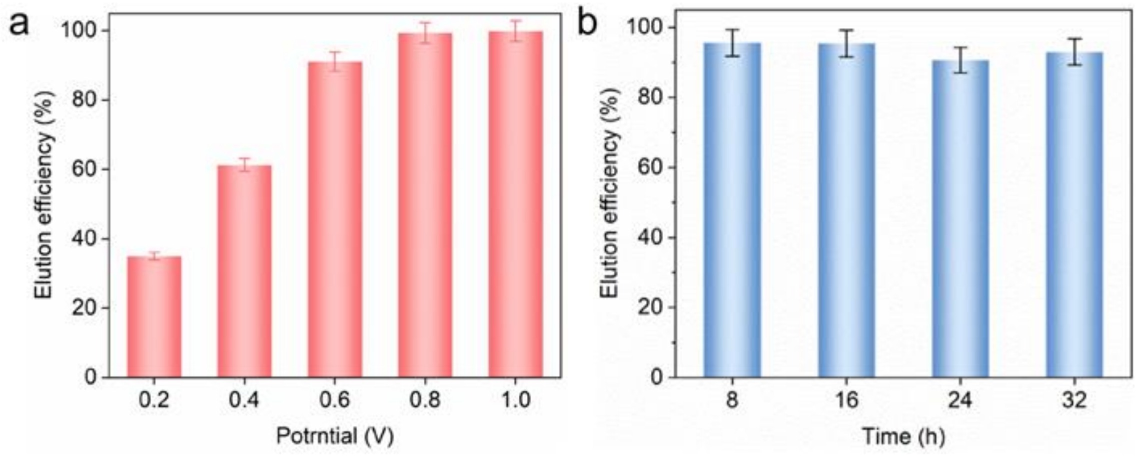

Supplementary Fig. 32 | (a) The electrochemical uranyl elution efficiency on P-CDP@CC at different reverse bias. (b) Desorption cycle performance of P-CDP@CC cathode low-level uranyl-containing radioactive organic wastewater.

5、If the presence of uranyl from a complex wastewater affects the urea degradation in the coupling system? To prove these points, the authors have to add an additional controlled test.

Response: We are grateful to the reviewer for providing the constructive comments. The interference from uranyl is indeed critically important that should be considered, thus we have compared the urea degradation performance in the case with and without 50 ppm uranyl. Obviously from the comparison, in the absence of uranyl, a degradation efficiency of 97.07% was achieved after 4 hours, whereas an obvious suppression was demonstrated in the presence of uranyl (**Supplementary Fig.32**). Such phenomenon may be attributed to the fact that the urea binding sites on TiO_2 would be occupied by uranyl (*Environ. Sci.Technol. 2022, 56, 13327−13337; Water Res.2024, 258, 121817*; *Nat. Sustain. 2025, 8, 682-691*), which inhibits the efficient urea degradation. It's noted that urea degradation is faster in kinetics than uranyl extraction, therefore, despite the inhibited urea degradation in the presence of uranyl, the coupled anodic oxidation and cathodic reduction are matched quite well in this couple system, achieving exceptional urea degradation efficiency of nearly 100 % and uranyl extraction efficiency of 99.08% after 8 hours in complicated wastewater matrix.

urea concentration is high as 12000 ppm (**Supplementary Fig. 30b**). Thereafter, the urea degradation performances in the case with and without 50 ppm uranyl were compared. Obviously from the comparison, in the absence of uranyl, a degradation efficiency of 97.07% was achieved after 4 hours, whereas an obvious suppression was demonstrated in the presence of uranyl (**Supplementary Fig. 31**). Such phenomenon may be attributed to the fact that the urea binding sites on TiO_2 would be occupied by uranyl, which inhibits the efficient urea degradation. It's noted that urea degradation is faster in kinetics than uranyl extraction, therefore, despite the inhibited urea degradation in the presence of uranyl, the coupled anodic oxidation and cathodic reduction are matched quite well in this couple system.

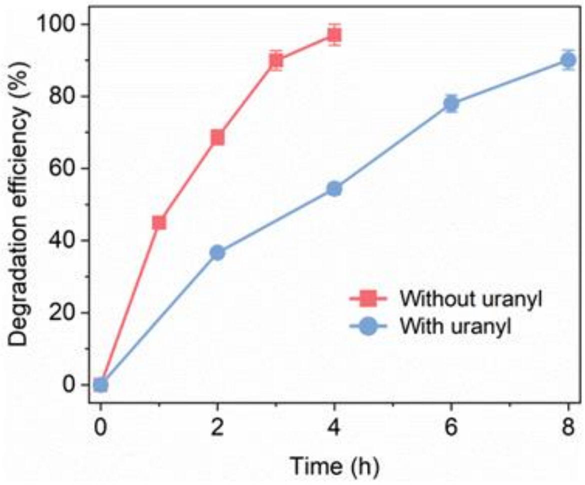

Supplementary Fig. 31 | Urea degradation efficiency of Ni/TiO_2 in the absence and presence of 50 ppm uranyl

6、The authors analyzed uranyl content after reaction in a complicated organic wastewater matrix. If the presence of organic matter would affect the quantitative analysis of uranyl? The authors should check the standard curve and compare the standard curves of uranyl with and without organic matter.

Response: We appreciate the constructive comment from the reviewer. The standard curves of uranyl both in the presence and in the absence of organic matter have been compared (**Supplementary Fig.26**). Notably, the R^2 values for the curves are 0.9996 and 0.9994, respectively, indicating a high degree of correlation and accuracy. Obviously from the comparison, it is confirmed that the quantitative analysis of uranyl remains unaffected by the presence of organic matter.

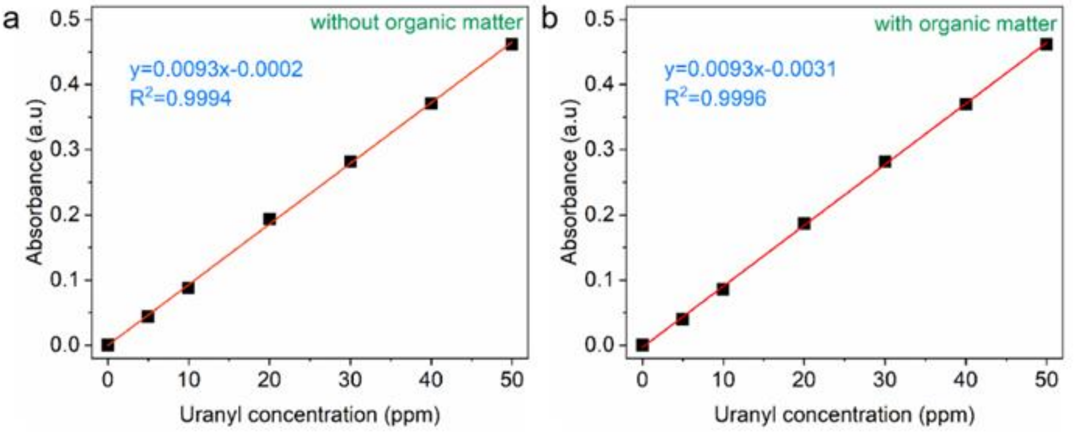

Supplementary Fig. 25 | The standard curves of uranyl concentration-absorbance (a) without organic matter; (b) with organic matter.

7、There are numerous errors of detail in the text, for example, XRD characterizes TiO_2 as anatase, while XPS characterizes as rutile; in Fig. 2f, the authors should clearly state the optimal voltage for further studies; in the description of C_{dl}, the slope of TiO_2 and Ni/TiO_2 doesn't match the Supplementary Fig. 17c. Please double check the whole text.

Response: We appreciate the carefully checking to the text. We have carefully corrected the spelling and clerical errors you pointed out and have thoroughly checked the entire manuscript and corrected the similar errors as marked in red.

8、To clarify that such a strategy is more promising for scale-up application, the authors should further compare the performance and cost of this work with other published works.

Response: We are grateful to the reviewer for providing the constructive comment. As shown in the following Table (**Supplementary Table 7**), we calculated the cost of the coupling system, which was approximately 82 USD for the extraction of 1 kg U and the simultaneous degradation of 1 kg urea, much lower than other electrochemical methods and physical adsorption methods reported in the literature (*J. Cleaner Prod. 2023, 388, 136006; Water Res. 2024, 257, 121683; J. Hazard. Mater. 2024, 464, 133002; Adv. Mater. 2025, 37, e08705; Chem. Eng. J. 2025, 519, 165635; Chem. Eng. J. 2024, 502, 157893; Nat. Commun. 2025, 16, 3574*). Furthermore, in low-level radioactive organic wastewater, the uranyl extraction efficiency and urea degradation efficiency were 99.08% and nearly 100%, respectively, which also exceed those performances from previously reports via uranyl adsorption from wastewater or via electrocatalytic reduction of uranyl.

In summary, our proposed novel bipolar coupling system exhibits higher advantages both in the

performance and the economic feasibility, thus more promising for scale-up application. We have added more discussion and cited the related publications to fully compare the performance and cost with other literatures in the revised manuscript.

U and the degradation of 1 kg urea (**Supplementary Table 6**), much lower than that with other electrochemical methods and physical adsorption methods reported in the literature (**Supplementary Table 7**).

Supplementary Table 7 | A comparison of the performance and cost of the bipolar coupling system with those of the previously reported system.

	Materials	Composition	Performance		Cost	Ref.
			Uranyl extraction efficiency	Organic matter removal efficiency		
Adsorption Method	AL-PEI/GMS	20 mg/L U	95.6 %	/	150 USD/kg	1
	PPN-3	20 mg/L U+ 15 mg/L HA	325.41 mg/g	/	200 USD/kg	2
	MCP-5	20 mg/L U+ 15 mg/L HA	304.31 mg/g	/	2360 USD/kg	3
Electrochemical method	ZrN/copper foam	23.7 mg/L U	82.4 %	/	85.7 USD/kg	4
	G-CCA/PAO	0.838 mg/L U	97.98 %	/	3150 USD/kg	5
	PTFE	0.259 mg/L U	82.6 %	/	747.9 USD/kg	6
	PAECOF-AO @CC	50 mg/L U	93.80%	/	500 USD/kg	7
	Photoanode: Ni/TiO_2 Cathode:P-CDP@CC	50 mg/L U+ 100 mg/L urea	99.08 % (653.99 mg/g)	Nearly 100 %	82 USD/kg	This work

Reviewer #2 (Remarks to the Author):

This study reports a photoelectrochemical (PEC) system based on a Ni nanoparticle-modified TiO_2 photoanode and a P-CDP@CC cathode for simultaneous uranyl ion extraction and urea degradation in a single-compartment cell. The work tackles a relevant environmental issue by integrating resource recovery with pollutant removal, in line with circular economy principles. While the reaction kinetics are commendable, the underlying concept of uranium extraction itself is not novel at all, as it has been previously documented as follows.

1. "Simultaneously reducing UO_2^{2+} and abating organic pollutants by a self-driven photoelectrocatalytic system with urea modified carbon fibre cathode", Chem. Eng. J. Volume 524, 169470

2. "Simultaneous removal and recovery of uranium from aqueous solution using TiO_2 photoelectrochemical reduction method", J. Radioanal. Nuclear Chem., Volume 313, pages 59–67, (2017)

This should be more suitable for a more specialized journal. Nevertheless, the following points require clarification and stronger justification:

Response: We appreciate sincerely all the constructive and critical comments from the reviewer, which inspire us thinking more comprehensively about the strategy and the mechanism. We apologize for the unclear description about the wastewater background.

We focus on the radioactive organic wastewater, which is a typical nuclear wastewater generated from the production of nuclear fuel for fourth-generation high-temperature gas-cooled reactor, containing a certain amount of uranyl (50~100 ppm), high ammonium nitrate (~800 ppm) and high co-existing urea (~12000 ppm), while the pH usually fluctuating near 9.0. Managing the treatment of such low-level radioactive organic wastewater is therefore highly desired, since it is able to alleviate the depletion of conventional uranium resources with simultaneous degradation of organic pollutants. Whereas according to our survey in the website of "Web of science" with the topic of "low-level radioactive organic wastewater" and keywords of "uranium" and "urea", there turned out only one reported study with limited uranyl extraction efficiency (see following screenshotted) perhaps due to the fact that such complicated wastewater matrix would bring in great challenges for those traditional treatment technologies including adsorption, ion exchange, and chemical precipitation.

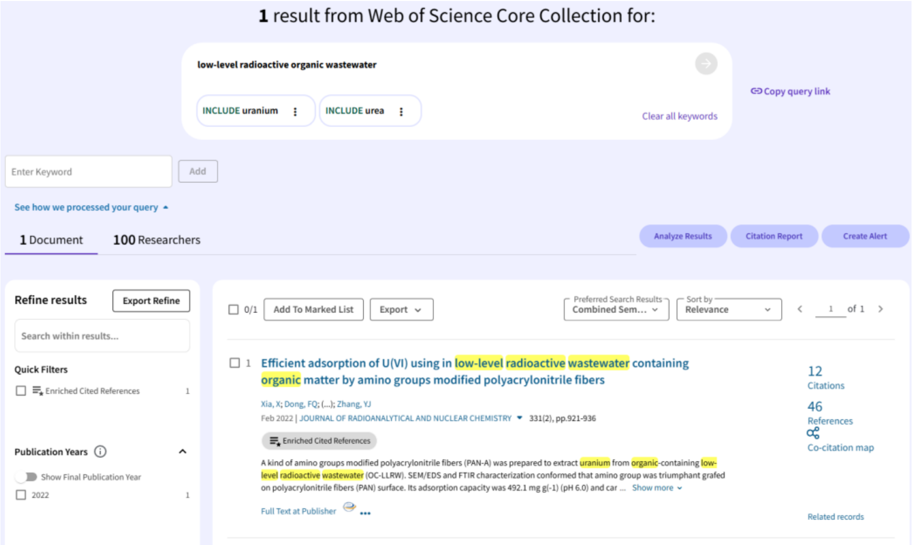

Based on the aforementioned challenge, for the first time our group proposed a cost-efficient photoelectrochemical (PEC) coupling system by coupling urea degradation and uranyl extraction for such radioactive organic wastewater management. Compared with the reported performance, the uranyl extraction efficiency in our work is more than 5-fold increased, with the first time considering the simultaneous treatment of urea.

Thank you very much for providing us the two nice articles, which we have thoroughly read. For Article 1, although the couple system was applied, the specific wastewater background was not presented, and seawater is employed as the application environment in the “application prospects” section. For Article 2, it only mentioned uranyl extraction using the photoelectrochemical method but without applying for any specific wastewater.

In addition, we have made point-by-point response to the comments and supplemented the related data and explanation and revised the manuscript carefully according to the comments. Thank you very much again for your time and endeavor.

1. A mechanistic explanation should be provided for the notably lower extraction efficiency observed at pH = 3.

Response: We are grateful to the reviewer for the constructive comment. The pH not only controls uranyl species in solution, but also affects the protonation of the hydroxyl groups at electrode surface.

According to the literatures (*Nat. Commun. 2024, 15, 9124*; *Nat. Commun. 2025, 16, 2361*), uranyl mainly exists in the form of UO_2^{2+} at pH 3 and pH 4, while further alkaline condition (pH 5-8)

would increase in the content ratio of the positively charged multinuclear hydroxide species of uranyl, such as $(UO_2)_2(OH)_2^{2+}$, $(UO_2)_3(OH)_5^{+}$,and $(UO_2)_4(OH)_7^{+}$, and at pH 8-10 the negatively charged uranyl species such as $(UO_2)_3(OH)_7^{-}$ and $UO_2(OH)_3^{-}$ are the prominent species.

Previous studies (*Nat. Commun. 2024, 15, 453*; *Nat. Sustain. 2022, 5, 71-80*) suggest that at low pH, hydroxyl groups on the P-CDP@CC electrode undergo protonation, leading to electrostatic repulsion between electrode surface and uranyl species. At higher pH, such repulsion effect is largely weakened since electrode surface tends to be electrically neutral charged.

Thus it turned out that with pH increasing, the uranyl extraction performance is increased as shown in **Fig. 2e**.

which is supposed due to the sluggish electron/ion transportation[38]. The pH not only controls uranyl species in solution, but also affects the protonation of the hydroxyl groups at electrode surface, thus the influence of pH on the electrolytic uranyl extraction over P-CDP@CC electrode was further evaluated. As illustrated in **Fig. 2e**, when pH is as low as 3.0, only 13 % of uranyl is extracted, attributing to the protonation of the hydroxyl groups on the P-CDP@CC electrode, leading to electrostatic repulsion between electrode surface and the uranyl cations. At pH 4.0, the extraction sharply improved to 58.13 %, and the optimal extraction of 96.99 % is obtained at pH 5.0. With pH increases to 6 and 9, the extraction remained consistently above 90%, which was supposed due to that the repulsion effect is largely weakened since electrode surface tends to be electrically neutral charged. Hence, pH value of the electrolyte was set to 5 for further studies. Increasing the applied

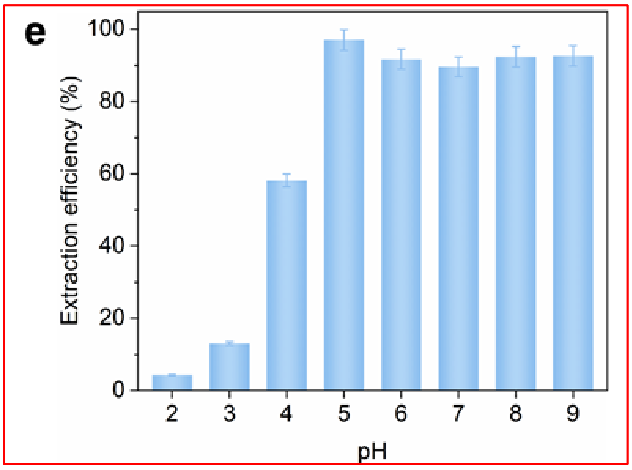

Fig. 2 | e Electrochemical extraction of uranyl on P-CDP@CC electrode under different pH conditions.

2. The treatment performance for both uranyl extraction and urea degradation should be assessed under acidic conditions representative of real uranium-containing effluents, to better demonstrate practical applicability.

Response: Thank you very much for the question. We apologize for the unclear description about the wastewater background and we deeply regret any misunderstanding that may have arisen from this. According to our survey (*J. Radioanal Nucl. Ch. 2022, 331, 921-936; Environ. Pollut. 2024, 344, 123269*), the detailed parameters of the low-level radioactive organic wastewater was shown in **Supplementary Table 5**. It's noted that the pH of this radioactive organic wastewater usually fluctuates near 9.0. Therefore, to better verify the practical applicability in low-level radioactive organic wastewater, we assembled the bipolar coupling system for simultaneous urea degradation and uranyl extraction at pH 9. **Fig. 6b** showed that after 8 hours with a constant voltage of 3 V at pH 9, an exceptional uranyl extraction efficiency of 99.08% and a urea degradation efficiency of nearly 100% were achieved, which means the couple system shows high performance even under real wastewater conditions, providing a feasible strategy for upcycling uranium resources and purifying organic pollutants from complicated wastewater matrix.

Supplementary Table 5 | The detailed constituents of low-level uranyl-containing radioactive organic wastewater.

Constituents	Concentration (ppm)
U(VI)	75.32
Urea	100
NH_4NO_3	800~8000
Ca	15.271
Mg	4.977
Si	2.156
Sr	0.370
Al	0.370
Fe	0.094
pH	9

3. The single-compartment cell design raises a fundamental concern regarding possible re-oxidation of the cathodic product. The manuscript should include a clear mechanistic discussion and supporting evidence showing how this issue is avoided in the present system.

Response: We appreciate the constructive comment from reviewer. Whether the tetravalent uranyl product on the electrode is reoxidized to hexavalent state, for sure, is an important issue to take into account. Notably, the cathodic products were deposited on the P-CDP@CC electrode during the extraction process, without falling into the electrolyte and migrating to the anode for reoxidized, which had been reported by many publications (*Nat. Commun. 2025, 16, 2012; Nat. Water. 2025, 3, 937-948; Water Res. 2024, 258, 121817; Environ. Sci. Technol. 2022, 56, 13327-13337*) although the uranyl in these publications was not from waste matrix as mentioned in our work.

According to your comment, we have checked the used P-CDP@CC electrodes immersed in reaction electrolyte with different resting times through X-ray photoelectron spectroscopy (XPS). Compared

with the fresh used P-CDP@CC electrode, XPS results (**Supplementary Fig.15**) showed no obvious change of uranium (IV) ratio on the surface of the electrode after resting for 2 hours, demonstrating that the cathodic product can be remained quite well, even long-time resting under open cell.

It's also confirmed that only with positive bias during the elution process, the extracted tetravalent uranyl dioxide on P-CDP@CC electrode was found to be oxidized back to soluble U(VI), which is consistent with the results from many literatures, e.g. *Nat. Sustain. 2025, 8, 682-691*; *Nat. Energy. 2017, 2, 17007*; *Chem. Eng. J, 2023, 457, 141221; Chem. Eng. J, 2025, 519, 165635*.

Therefore, we believe that in our electrochemical reduction process, re-oxidation of the cathodic product is negligible.

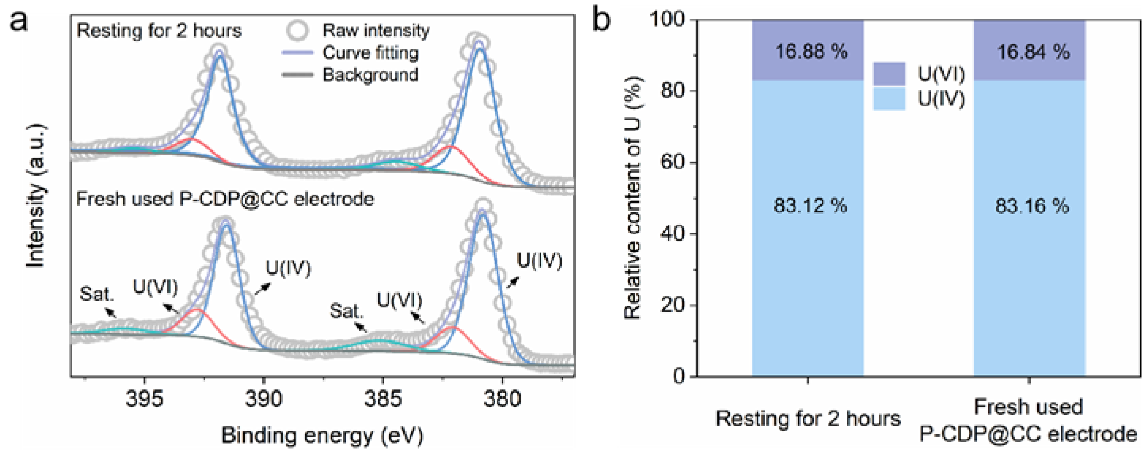

Supplementary Fig. 14 | (a) XPS spectra of U 4*f* for the used P-CDP@CC electrode before and after resting. (b) The content of different valence states of uranyl (U(VI) and U(IV)) for the used P-CDP@CC electrode before and after resting.

4. The considerable gap between nearly complete urea degradation (~100%) and lower TOC/TN removal (~64%) suggests accumulation of intermediate products. Further analysis to identify these intermediates and propose a detailed degradation pathway is needed.

Response: We are grateful to the reviewer for the constructive comment. Concerning the mechanism about urea degradation is indeed critically important that should be considered, thus we have identified intermediates and further explored the degradation pathway through ion chromatography (IC) and the colorimetric method. **Supplementary Fig.24** shows the intermediates comprised nitrate ions (NO_2^-), nitrate (NO_3^-), ammonia (NH_4^+), carbonate (CO_3^{2-}), and cyanate (OCN^-). Specifically, the concentration of NO_3^--N displays a monotonically increasing tendency along with the reaction

time, and the maximum concentration of 8.76 mg/L is obtained at 4 hours. Notably, the concentrations of NO_2^--N and NH_4^+-N undergo an initial increase and lateral decline, indicating that NO_2^- and NH_4^+ as intermediates were accumulated progressively at the initial stage and would further transform into products such as NO_3^-, N_2, and NH_3. In addition, the concentration of OCN^- and CO_3^{2-} in the PEC system showed upswing trends as the reaction proceeded. The accumulation of OCN^- is supposed to be the main reason for the lower TOC/TN removal (~64%).

Furthermore, as depicted in **Supplementary Table 4**, when Na_2SO_3 was used as h^+ scavenger, urea degradation almost ceased, confirming the important role of the photogenerated holes in urea degradation. When TBA was used as a quenching agent for·OH radicals, the degradation efficiency of urea was markedly reduced. These results imply that the urea degradation is jointly participated by photogenerated holes and ·OH radicals. Moreover, according to the literatures (*Nanoscale Adv.,2025,7, 3055; J. Mater. Chem. A,2019,7,6327; Chem. Soc. Rev., 2024,53, 1552*), the presence of active species (·OH and h^+) was also proved playing an important role in the urea degradation via the PEC process.

According to the above analysis, we proposed the probable mechanism for PEC urea degradation on the Ni/TiO_2 photoanode (**Supplementary Fig.25**), which includes both indirect degradation by ·OH radicals and direct degradation by photogenerated holes. For the indirect pathway, ·OH would be generated through water oxidation by the photogenerated holes transferred to the interface of the Ni/TiO2 photoanode. ·OH converts one of the amino groups in urea to a nitroso group and then to a nitro group, leading to the formation of nitroformamide (NH_2CONO_2) (*J. Clean. Prod, 2023, 419, 138200; Environ. Sci. Pollut. Res., 2019, 26, 1044-1053*), which then undergoes hydrolysis to releases NO_2^- and $COONH_2^-$. NO_2^- can be further rapidly transformed into NO_3^- by ·OH. The resulting $COONH_2^-$ can undergo two different oxidation pathways. One is that $COONH_2^-$ is decomposed into NH_4^+, which was further oxidized to N_2 (*Nat. Energy, 2021, 6, 904-912*); the other is that it is decomposed into CO_3^{2-} and NO_3^- by ·OH. For the direct pathway, urea decomposes into ammonia (NH_3) and cyanate ions (OCN^-) with the aid of photogenerated holes (*ChemSusChem, 2025; e202501772*). Subsequently NH_3 is further oxidized to NO_2^- or N_2, while OCN^- is oxidized ultimately to produce NH_3, NO_3^- and CO_3^{2-}.

We have added more discussion about the intermediates and possible pathway of urea degradation in the revised manuscript and supporting information.

Simultaneously, a series of intermediates were identified through ion chromatography (IC) and the colorimetric method. **Supplementary Fig.23** shows the intermediates comprised nitrate ions (NO_2^-),

nitrate (NO_3^-), ammonia (NH_4^+), carbonate (CO_3^{2-}), and cyanate (OCN^-). Specifically, the concentration of NO_3^--N displays a monotonically increasing tendency along with the reaction time, and the maximum concentration of 8.76 mg/L is obtained at 4 hours. Notably, the concentrations of NO_2^--N and NH_4^+-N undergo an initial increase and lateral decline, indicating that NO_2^- and NH_4^+ as intermediates were accumulated progressively at the initial stage and would further transform into products such as NO_3^-, N_2, and NH_3. In addition, the concentration of OCN^- and CO_3^{2-} in the PEC system showed upswing trends as the reaction proceeded. Moreover, compared to photocatalysis (PC)

According to the above analysis, we proposed the probable mechanism for PEC urea degradation on the Ni/TiO_2 photoanode (**Supplementary Fig.24**), which includes both indirect degradation by ·OH radicals and direct degradation by photogenerated holes. For the indirect

pathway, ·OH would be generated through water oxidation by the photogenerated holes transferred to the interface of the Ni/TiO_2 photoanode. ·OH converts one of the amino groups in urea to a nitroso group and then to a nitro group, leading to the formation of nitroformamide (NH_2CONO_2), which then undergoes hydrolysis to releases NO_2^- and $COONH_2^-$[44,45]. NO_2^- can be further rapidly transformed into NO_3^- by ·OH. The resulting $COONH_2^-$ can undergo two different oxidation pathways. One is that $COONH_2^-$ is decomposed into NH_4^+, which was further oxidized to N_2[46]; the other is that it is decomposed into CO_3^{2-} and NO_3^- by ·OH. For the direct pathway, urea decomposes into ammonia (NH_3) and cyanate ions (OCN^-) with the aid of photogenerated holes[47]. Subsequently NH_3 is further oxidized to NO_2^- or N_2[48], while OCN^- is oxidized ultimately to produce NH_3, NO_3^- and CO_3^{2-}.

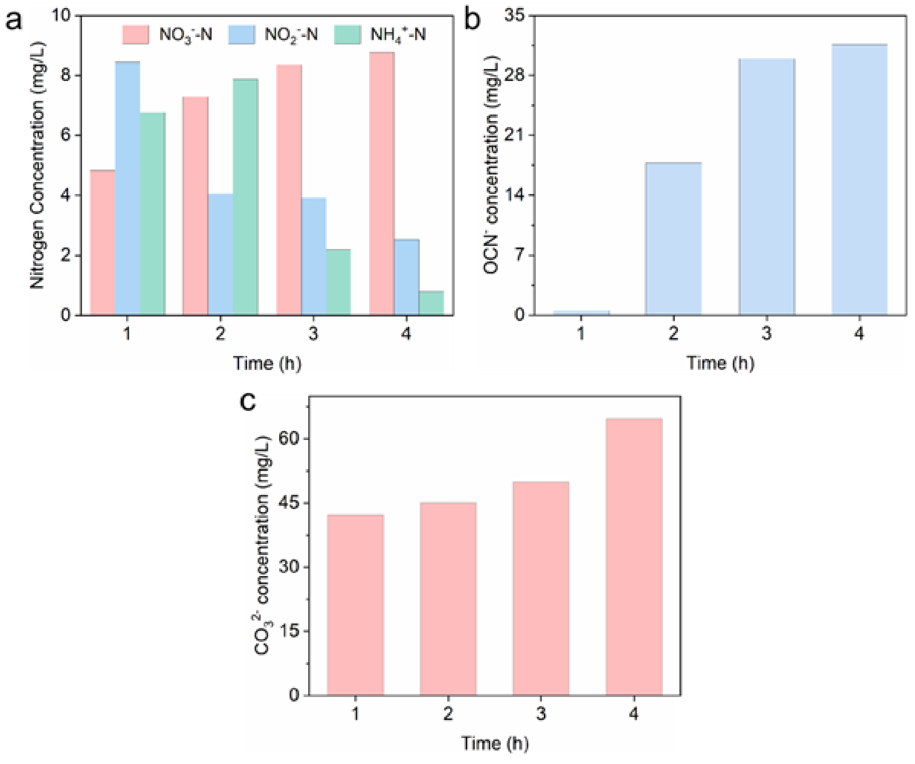

Supplementary Fig. 23 | (a) The concentration of produced nitrogen species for Ni/TiO2. (b) The concentration of produced carbon species for Ni/TiO2.

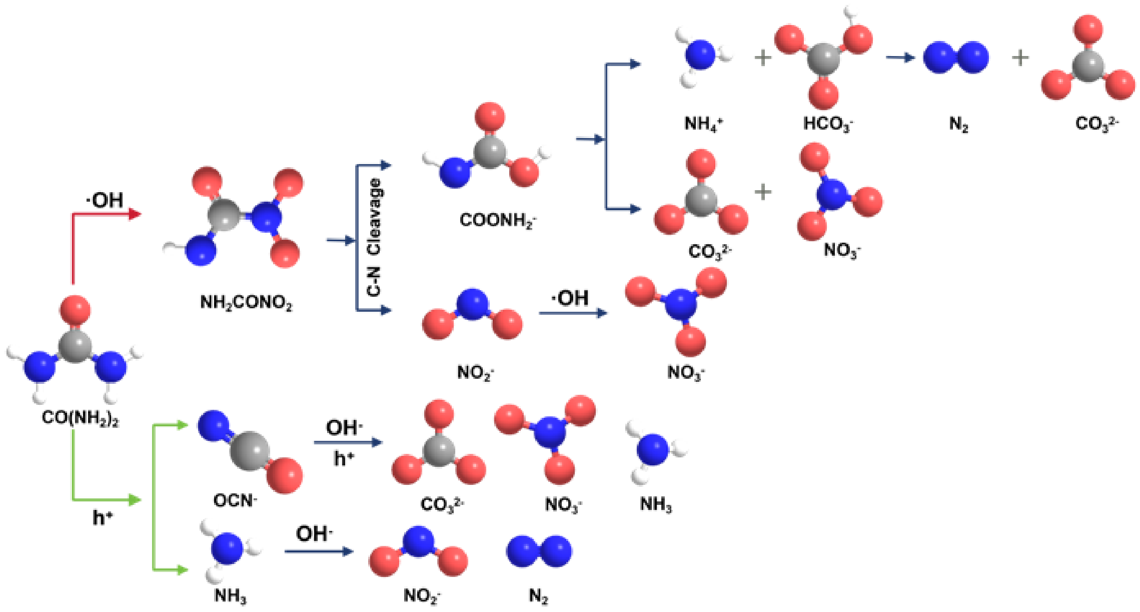

Supplementary Fig. 24 | A diagram of the Ni/TiO2 photoanode reaction mechanism.

5. The rationale for the specific urea concentration used in the coupled system should be explained. In addition, the effect of varying urea concentrations on uranyl removal performance should be

systematically investigated to elucidate the interaction between the two processes.

Response: We appreciate the constructive comment from the reviewer. The interference from urea for uranyl extraction is indeed critically important that should be considered, which inspired us to go straight at this problem. According to the literatures (*AIP Conf. Proc. 2016, 1767, 020019*;), uranyl would complex with urea to form complexes such as $UO_2(NO_3)\cdot 2(NH_2)_2CO$, $UO_2(NO_3)\cdot 4(NH_2)_2CO\cdot H_2O$ and $UO_2(NO_3)\cdot 5(NH_2)_2CO\cdot H_2O$ under highly acidic conditions. Considering the fact that the pH of the concerned radioactive organic wastewater fluctuates after near 9.0, we exclude the existence of such complexes from uranyl and urea.

Moreover, we have evaluated the uranyl extraction kinetics on P-CDP@CC electrode with the urea concentration varied from 10 ppm to 12000 ppm. As shown in **Supplementary Fig.31a**, with the urea concentration varied from 10 ppm to 12000 ppm, slight decrease in the uranyl extraction efficiency was observed in the initial 2 hours, which was ascribed to the competitive urea and uranyl adsorption on P-CDP@CC electrode surface. However, when the reaction time was prolonged to 4 hours and then 8 hours, regardless of the initial concentration of urea, the uranyl extraction efficiency by P-CDP@CC electrode remained consistently above 90 %, indicating that the excessive urea could only interfere the initial uranyl reduction kinetics and with the persistent electric field, the uranyl could be reduced and grown into UO_2 nanoparticle even when urea concentration is high as 12000 ppm (**Supplementary Fig.31b**).

In the coupled system, 100 ppm was adopted as the initial concentration of urea for further studies, based on the following reasons: For the quantitative analysis of urea, we use the colorimetric method to analysis the aqueous sample directly without dilution, so such concentration of urea could minimize measurement errors; For the mechanism analysis, such concentration enables the clear identification of the degradation intermediate.

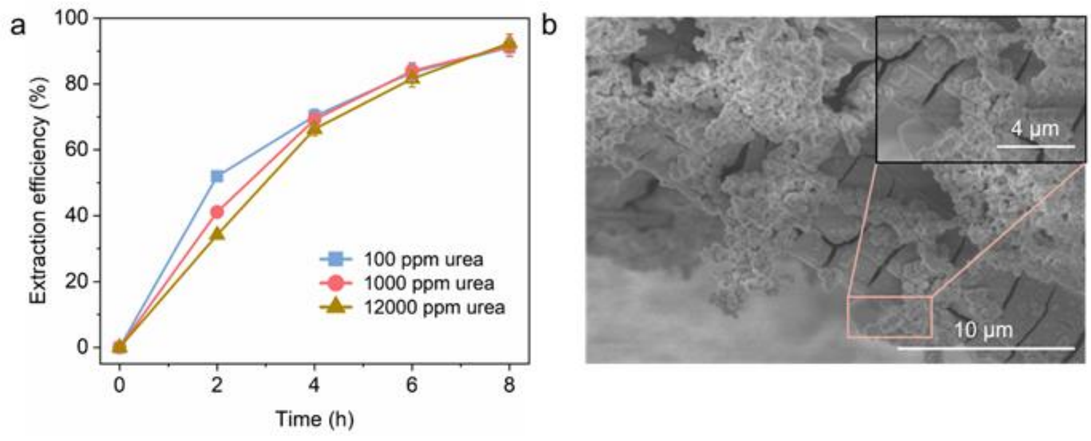

Supplementary Fig. 30 | (a) Uranyl extraction efficiency with the urea concentration varied from 100 ppm to 12000 ppm.(b) SEM image of P-CDP@CC after uranyl extraction with 12000 ppm urea.

After that, the interaction between the two processes of uranyl extraction and urea degradation was further systematically elucidated. It was reported that uranyl would complex with urea to form complexes such as $UO_2(NO_3)\cdot 2(NH_2)_2CO$, $UO_2(NO_3)\cdot 4(NH_2)_2CO\cdot H_2O$ and $UO_2(NO_3)\cdot 5(NH_2)_2CO\cdot H_2O$ under highly acidic conditions[49]. Considering the fact that the pH of the concerned radioactive organic wastewater fluctuates after near 9.0, thus the existence of such complexes from uranyl and urea in coupling system were excluded. Furthermore, with the urea concentration varied from 10 ppm to 12000 ppm, slight decrease in the uranyl extraction efficiency was observed in the initial 2 hours (**Supplementary Fig. 30a**), which was ascribed to the competitive urea and uranyl adsorption on P-CDP@CC electrode surface. However, when the reaction time was prolonged to 4 hours and then 8 hours, regardless of the initial concentration of urea, the uranyl extraction efficiency by P-CDP@CC electrode remained consistently above 90 %, indicating that the excessive urea could only interfere the initial uranyl reduction kinetics and with the persistent electric field, the uranyl could be reduced and grown into UO_2 nanoparticle even when urea concentration is high as 12000 ppm (**Supplementary Fig. 30b**). Thereafter, the urea

6. The potential interference of co-existing ions commonly found in real wastewater—particularly NO_3^- and NH_4^+—on both uranyl extraction and urea degradation has not been systematically evaluated.

Response: We are grateful to the reviewer for the constructive comment. Based on your comment, we have supplemented the influence of NH_4^+ and NO_3^- concentration change in coupling system. Considering the detailed constituents of low-level uranyl-containing radioactive organic wastewater, the initial concentration of NH_4^+ and NO_3^- varied from 0.8 to 8 g/L. Therefore, we supplemented the test with the bipolar PEC system and initial NH_4NO_3 solution varied from 0.8 to 8 g/L. As illustrated in **Supplementary Fig.30**, regardless of the initial concentration of NH_4^+ and NO_3^-, the uranyl extraction efficiency and urea degradation remained consistently above 90 %, indicating that the NH_4^+ and NO_3^- concentrations did not exhibit an obvious influence on the uranyl extraction and urea degradation in coupling system.

photocatalytic processes. Moreover, regardless of the initial high concentration of NH_4^+ and NO_3^-, the uranyl extraction efficiency and urea degradation remained consistently above 90 % (**Supplementary Fig. 29**), indicating that the NH_4^+ and NO_3^- concentrations did not exhibit an obvious influence on the uranyl extraction and urea degradation in coupling system.

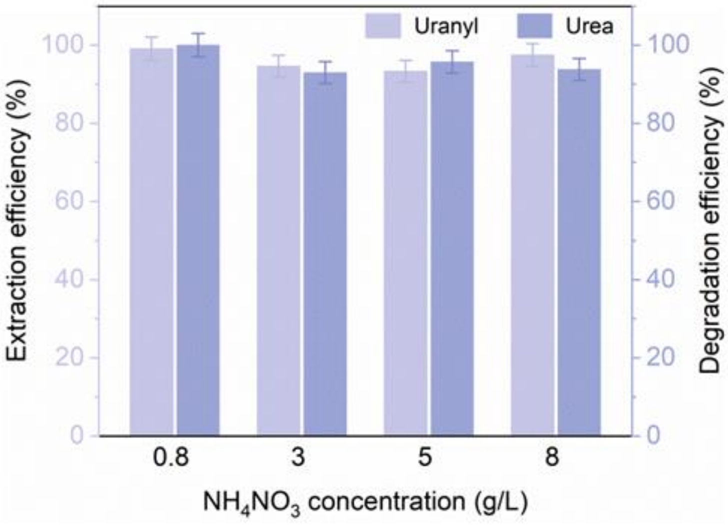

Supplementary Fig. 29 | Uranyl extraction efficiency and urea degradation efficiency in coupling system as the NH_4NO_3 concentrations varied from 0.8 g/L to 8 g/L.

7. The schematic of the "bipolar coupling system" (Fig. 5) is oversimplified. It should be improved to better illustrate the function of the material designs, such as the role of Ni nanoparticles on TiO_2 nanotubes in facilitating charge separation and enhancing urea oxidation kinetics.

Response: We sincerely appreciate the valuable suggestion provided. As suggested, we have now re-drawn this schematic to better illustrate the function of the photoanode and cathode. The related revision has been screenshotted as followed.

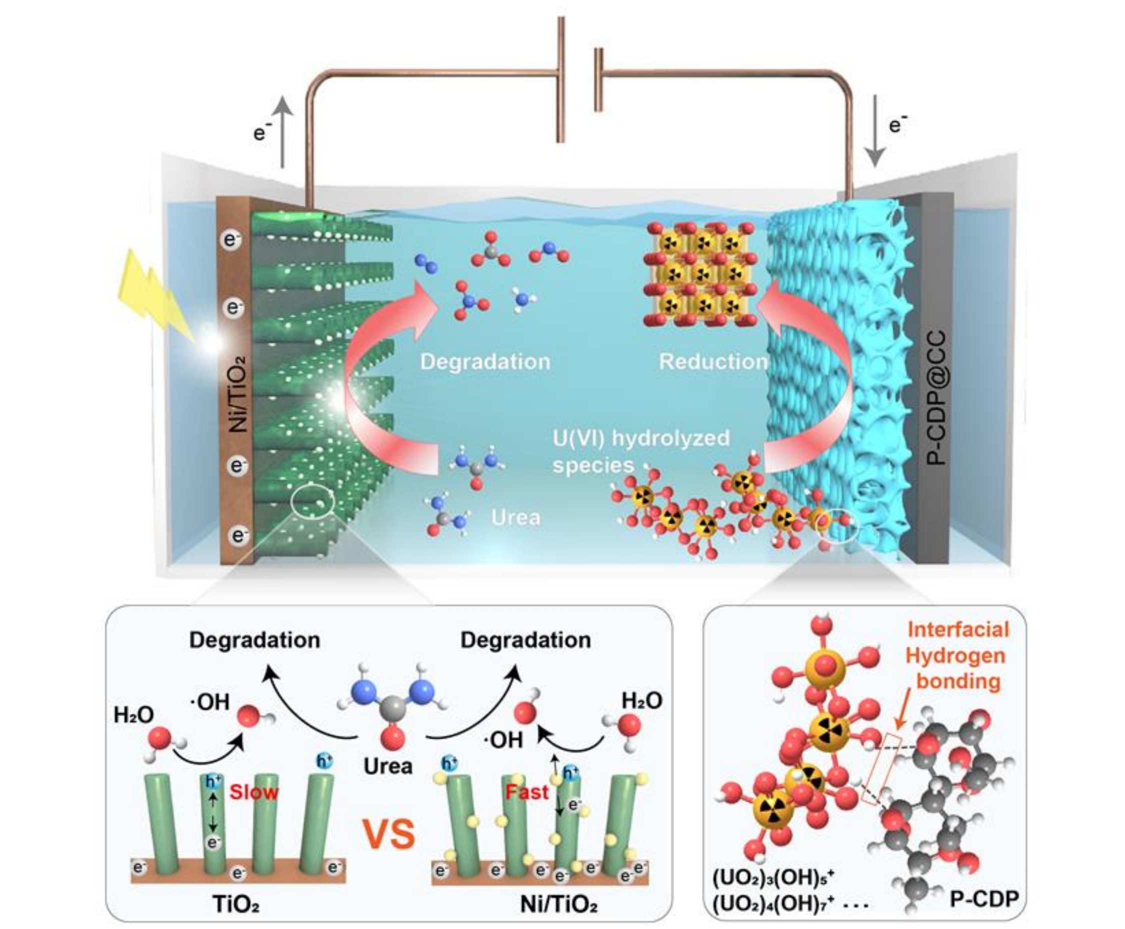

Fig. 5 | Possible reactive pathways of the bipolar coupling system. Ni/TiO_2 photoanode is combined with P-CDP@CC cathode to achieve a simultaneous urea degradation and uranyl extraction.

8. The advantage of the coupled strategy should be underscored by direct performance comparisons with individual technologies, such as standalone adsorption or photocatalytic processes.

Response: We are grateful to the reviewer for providing the constructive comment. Inspired by your suggestion to validate the superiority of the coupled strategy, we have supplemented the performance evaluation with the additional controlled standalone technology, i.e., adsorption and photocatalytic processes. Notably, considerably dampened uranyl extraction of 8.38% and 3.96% with coupled urea degradation of 13.94% and 4.77% were observed via photocatalytic and physical adsorption,

respectively (**Supplementary Fig.29**). Such a distinct disadvantage could be attributed to two aspects: i) the intense competitive adsorption of organic matters against uranyl on P-CDP@CC electrode surface in the absence of electric field, blocking active sites crucial for uranyl extraction, ii) the competitive adsorption of uranyl for urea on the Ni/TiO_2 photoanode, inhibiting the efficient urea degradation (*Electrochim.Acta.2025, 514, 145641*).

By contrast, under the application of an electric field, accelerated migration of uranyl ions was able to alleviate the aforementioned interference effect.

Fig. 6b and **Supplementary Fig. 26-28** demonstrated exceptional uranyl extraction efficiency of 99.08% and urea degradation efficiency of nearly 100% at a current density of 4.5 mA cm^{-2} and cell voltage of 3 V, which is far higher than that of those standalone technologies, i.e., adsorption and photocatalytic processes. Moreover, regardless of the initial high concentration of NH_4^+ and NO_3^-,

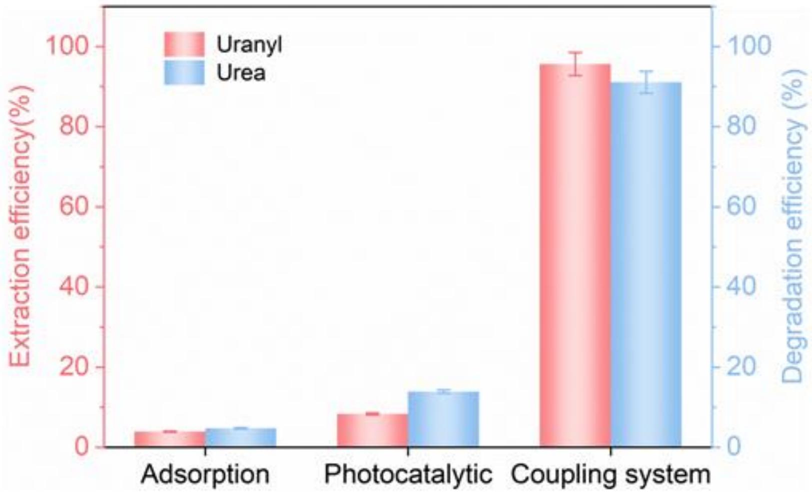

Supplementary Fig. 28 | Comparison of uranyl extraction efficiency with the adsorption, photocatalytic and coupling system.

9. Experimental evidence is required to determine whether the valence state of U changes after dissolution of UO_2(s) in 0.1 M Na_2CO_3 solution, specifically whether eluted uranium is in the +4 or +6 state.

Response: We greatly appreciate your professional comments on our article. Concerning the valence state of U after elution reaction, although in many literature such as *Nat. Commun. 2023, 12, 4149*; *Nat. Energy. 2017, 2, 17007*; *Nat. Water. 2023, 1, 887-898*, it was not clearly described, your comment was of great significance, which inspired us to go straight at this problem. Thus, the

valence state of uranium species in the electrolyte after elution with a reverse bias of 0.8 V was further checked and analyzed using the detailed U 4*f* spectrum. We use commercially available SiO_2 to load the possibly dispersed uranium species in the aqueous solution and dry the U-loaded SiO_2 at vacuum. As shown in **Supplementary Fig.12**, the deconvolution of the U 4*f* spectral peak yielded doublet peaks at U $4f_{7/2}$ (381.1 eV) and U $4f_{5/2}$ (391.8 eV), which corresponded to the U(VI) state. This result confirmed that the immobilized U on the electrode was oxidized into U(VI) during the elution process with reverse bias. We have added the corresponding discussion in the revised manuscript.

627.45 mg g^{-1} were achieved with an initial concentration of 100 ppm. Subsequently, with 0.1 M Na_2CO_3 electrolyte and 0.3 V vs. Ag/AgCl reverse bias, 99.86 % of immobilized U on the electrode can be oxidized into soluble U(VI), thereby achieving uranyl ultra-efficiently recovery (**Supplementary Fig. 10 and Supplementary Fig. 11**). The long-term utilization of such

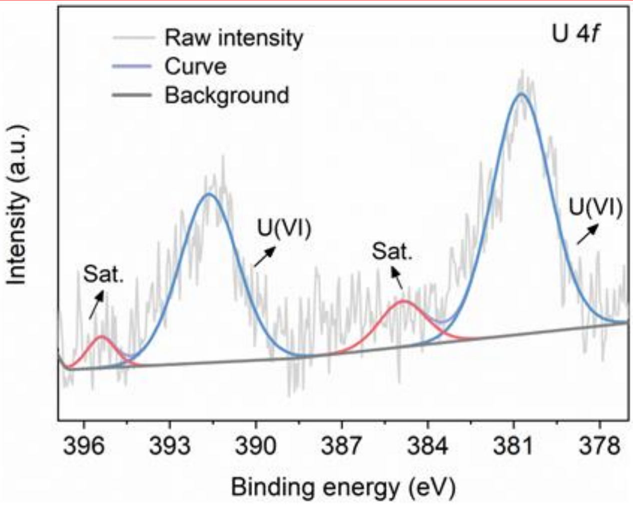

Supplementary Fig. 11 | U 4*f* spectra of P-CDP@CC electrode after elution process.

10. The study lacks a UO_2^{2+} selectivity assay. Selectivity against competing ions is essential in uranium extraction studies and must be included.

Response: We appreciate the constructive comment from the reviewer. As the reviewer pointed out, the interference from competing ions is indeed critically important that should be considered, thus we have tested the uranyl extraction of P-CDP@CC electrode in the presence of several co-existing ions which commonly existed in the wastewater of nuclear production, including Ca^{2+}, Mg^{2+}, Sr^{2+},

Al^{3+}, Fe^{3+}, and SiO_3^{2-} (**Supplementary Table 5**), according to the corresponding concentration reported in the literature (*Environ. Pollut. 2024, 344, 123269*). Notably, ICP-OES analysis showed that in the precipitate after extraction, the content proportion of U reached 99.15%, which was much higher than other interfering ions (**Supplementary Fig.14**), indicating that the P-CDP@CC electrode exhibited high anti-interfering ability to co-existing ions. Such high selectivity for uranium was attributed to the fact that only Fe^{3+} with quite low concentration was present as the variable valent metal in the wastewater system. We have added more discussion and supplemented the analysis method in the revised manuscript.

Supplementary Table 5 | The detailed constituents of low-level uranyl-containing radioactive organic wastewater.

Constituents	Concentration (ppm)
U(VI)	75.32
Urea	100
NH_4NO_3	800~8000
Ca	15.271
Mg	4.977
Si	2.156
Sr	0.370
Al	0.370
Fe	0.094
pH	9

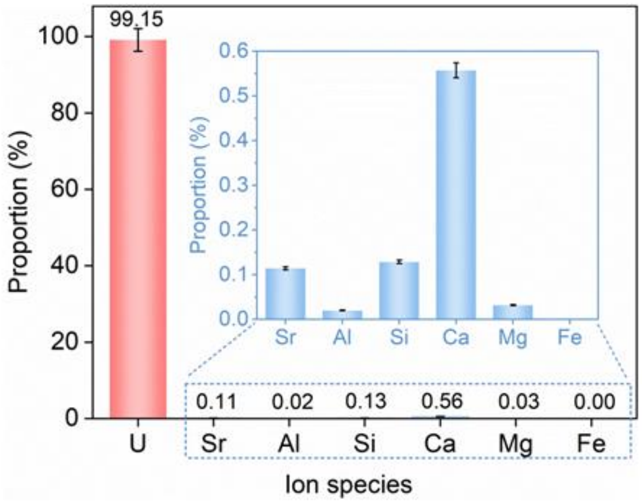

Supplementary Fig. 13 | The proportion of uranyl among the metal species in the precipitate after electronic extraction.

(**Supplementary Fig. 12**). Notably, in the presence of several co-existing ions, the proportion of U reached 99.15% among the metal elements in precipitate after electrochemical extraction, which was much higher than other interfering ions (**Supplementary Fig.13**), indicating that the P-CDP@CC electrode exhibited high anti-interfering ability to co-existing ions.

11. A comparison between the reduction of V and U should be provided, given that vanadium is a key competing cation in uranium extraction.

Response: We appreciate the constructive comment from the reviewer. Indeed, exploring the selectivity of the P-CDP@CC electrode to uranyl was critical for its' practical application in real wastewater, your comment was of great significance, which inspired us to go straight at this problem. However, based on the previous literature (*J. Radioanal.Nucl.Chem.2022, 331,921-936*; *Environ. Pollut. 2024, 344, 123269.*), although multiple interfering ions such as Ca^{2+}, Mg^{2+}, Sr^{2+}, Al^{3+}, Fe^{3+}, and SiO_3^{2-}, are indeed involved in the radioactive organic wastewater generated from the production of nuclear fuel for fourth-generation high-temperature gas-cooled reactor, vanadium ions are not present as the interfering ions. Simultaneously, we have supplemented the selectivity of uranyl over other ions, including Ca^{2+}, Mg^{2+}, Sr^{2+}, Al^{3+}, Fe^{3+}, and SiO_3^{2-}, but the precipitate after electrochemical extraction contained the proportion of other interfering ions were poor.

12. Appropriate correlation of distribution coefficients and an enrichment factor for uranium are currently lacking.

Response: We are grateful to the reviewer for providing the constructive comment. Based on your suggestion, we have supplemented the calculation of distribution coefficient and enrichment factor. The distribution coefficient, K_d, was calculated up to 7.01×10^5 mL/g (a K_d value exceeding 1.0×10^5 mL/g is usually considered as excellent material) (*Adv. Funct. Mater. 2025, e09597; Adv. Funct. Mater. 2025, e22094; Adv. Mater. 2021, 33, 2106621; Sep. Purif. Technol. 2025, 135442*), implying extremely outstanding advantage of the proposed coupled system for uranyl extraction. Subsequently, we also calculated the enrichment factor of the electrochemical system, which was 2249 for U (*Small, 2024, 20, 2302014*).

which agreed well with aforementioned analysis (**Supplementary Fig. 36**). In addition, the corresponding distribution coefficient (K_d) was calculated up to 7.01×10^5 mL/g (a K_d value exceeding 1.0×10^5 mL/g is usually considered as excellent material), demonstrating extremely outstanding advantage of the proposed coupled system for uranyl extraction. Subsequently, we also evaluated the enrichment factor of the electrochemical system, which was 2249 for U.

13. The system's performance should be discussed in the context of real conditions, where multiple uranium species, transition metals, and salts coexist. It is important to evaluate how the material would selectively extract trace U ions from a complex mixture of cations, especially when competing ions are present at much higher concentrations.

Response: We are grateful to the reviewer for providing the constructive comment. According to the reviewer's suggestion, the uranyl extraction experiments were carried out in low-level radioactive organic wastewater to validate its feasibility in realistic applications. This complicated wastewater consisted of high levels of ammonium nitrate (up to 800 ppm), uranyl (75.32 ppm), urea (100 ppm), and coexisting ions (Ca^{2+}, Mg^{2+}, Sr^{2+}, Al^{3+}, Fe^{3+}, and SiO_3^{2-}) (**Supplementary Table 5**), according to the corresponding concentration reported in the literature (*Environ. Pollut. 2024, 344, 123269*). Quantitative analysis showed that U proportion in the precipitate after electrochemical extraction is high as 99.40%, while other co-existing ions proportion below 0.30% (**Supplementary Fig.37**), implying high anti-interference capacity and applicability of P-CDP@CC electrode for complex wastewater. Such high selectivity for uranium was attributed to the fact that except the inert cations, only Fe^{3+} with quite low concentration was present as the variable valent metal in the wastewater

system.

We have added the discussion on the uranyl extraction performance of P-CDP@CC electrode from the complicated wastewater matrix in the revised manuscript.

(WT) analysis (**Fig. 6f**). ICP-OES results showed that U proportion in the precipitate after electrochemical extraction is high as 99.40%, while other co-existing ions proportion below 0.30%, which agreed well with aforementioned analysis (**Supplementary Fig. 36**). In addition, the

Supplementary Table 5 | The detailed constituents of low-level uranyl-containing radioactive organic wastewater.

Constituents	Concentration (ppm)
U(VI)	75.32
Urea	100
NH_4NO_3	800~8000
Ca	15.271
Mg	4.977
Si	2.156
Sr	0.370
Al	0.370
Fe	0.094
pH	9

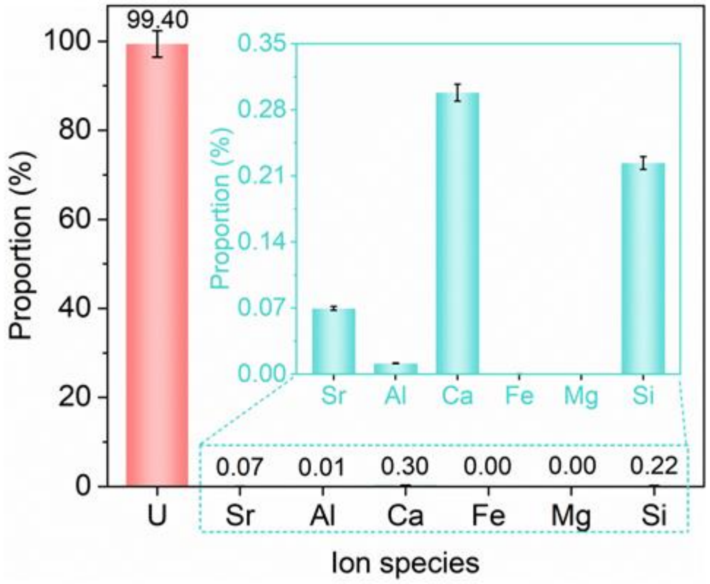

Supplementary Fig. 36 | The proportion of uranyl among the metal species in the precipitate after electronic extraction from complicated wastewater.

14. Unit formatting is inconsistent and must be standardized. Use standardized abbreviations throughout (e.g., "h" for hour, "mL" not "ml", "kg" not "Kg") in the text, figures, and tables.

Response: We appreciate the carefully checking to our manuscript. We have thoroughly checked the entire manuscript and standardized the abbreviations as marked in red.

Reviewer #1 (Remarks to the Author):

The authors have addressed each of my questions one by one, and I currently have no further inquiries.

Response: We are grateful to the reviewer for taking the time to evaluate our work and greatly appreciate the positive comments and support of our work.

Reviewer #2 (Remarks to the Author):

My opinion remains the same. The concept of PEC system that simultaneously oxidizes organic pollutants and reduces uranyl ions has been well demonstrated in the literature. There is little novelty in this study. This should be more suitable to a specialized journal.

Response: We would like to thank you for your professional review work and constructive comments on our manuscript. Thank you very much again for your time and endeavor. In this work, we concentrate on the treatment of low-level radioactive organic wastewater from the production of nuclear fuel for fourth-generation high-temperature gas-cooled reactor, which contains high ammonium nitrate (800~8000 ppm), a certain amount of uranyl (50~100 ppm) and high organic matter (800 ppm of polyvinyl alcohol, 12000 ppm of urea, 8000 ppm of tetrahydrofurfuryl alcohol). The treatment of such low-level radioactive organic wastewater is of great significance, not only alleviating the depletion of conventional uranium resources but also simultaneously purifying co-existing organic pollutants. According to our survey, although plenty of research focused on exploring how to efficiently remove uranyl or the co-existing organic matter from wastewater, developing the uranyl extraction coupled with urea degradation for low-level radioactive organic wastewater treatment in an integrated process is still lacking so far (see following screenshotted), since such complicated wastewater matrix would bring in great challenges to conventional treatment technologies, such as adsorption, ion exchange, and chemical precipitation. Hence, we construct a high-performance photoelectrochemical system for uranyl extraction and the simultaneous degradation of co-existing urea to manage the treatment of such radioactive organic wastewater.

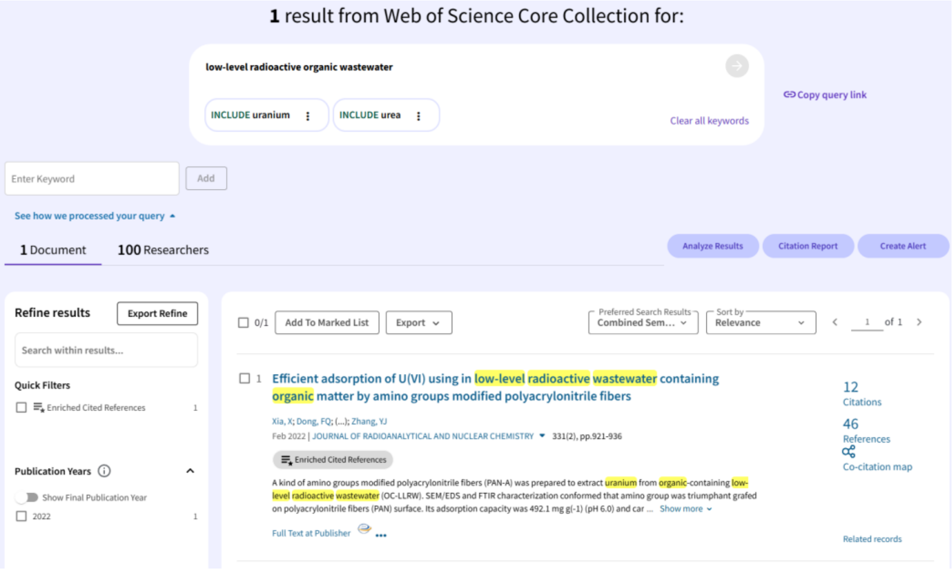
1 result from Web of Science Core Collection for:
low-level radioactive organic wastewater
INCLUDE uranium
INCLUDE urea
Clear all keywords
Copy query link
Enter Keyword
Add
See how we processed your query
1 Document
100 Researchers
Analyze Results
Citation Report
Create Alert
Refine results
Export Refine
Search within results...
Quick Filters
Enriched Cited References 1
Publication Years
Show Final Publication Year
2022 1
0/1
Add To Marked List
Export
Preferred Search Results
Combined Sem...
Sort by
Relevance
1 of 1
1 Efficient adsorption of U(VI) using in low-level radioactive wastewater containing organic matter by amino groups modified polyacrylonitrile fibers
Xia, X; Dong, FQ; (...); Zhang, YJ
Feb 2022 | JOURNAL OF RADIOANALYTICAL AND NUCLEAR CHEMISTRY 331(2), pp.921-936
Enriched Cited References
A kind of amino groups modified polyacrylonitrile fibers (PAN-A) was prepared to extract uranium from organic-containing low-level radioactive wastewater (OC-LLRW). SEM/EDS and FTIR characterization conformed that amino group was triumphant grafed on polyacrylonitrile fibers (PAN) surface. Its adsorption capacity was 492.1 mg g(-1) (pH 6.0) and car ... Show more
Full Text at Publisher
12
Citations
46
References
Co-citation map
Related records
